# Fitbit-Based Interventions for Healthy Lifestyle Outcomes: Systematic Review and Meta-Analysis

**DOI:** 10.2196/23954

**Published:** 2020-10-12

**Authors:** Mickael Ringeval, Gerit Wagner, James Denford, Guy Paré, Spyros Kitsiou

**Affiliations:** 1 École des Sciences de la Gestion Université du Québec à Montréal Montreal, QC Canada; 2 Research Chair in Digital Health HEC Montreal Montreal, QC Canada; 3 Department of Management Faculty of Social Sciences and Humanities Royal Military College of Canada Kingston, ON Canada; 4 Department of Biomedical and Health Information Sciences College of Applied Health Sciences University of Illinois at Chicago Chicago, IL United States

**Keywords:** Fitbit, wearables, healthy lifestyle, meta-analysis, literature review, fuzzy-set qualitative comparative analysis

## Abstract

**Background:**

Unhealthy behaviors, such as physical inactivity, sedentary lifestyle, and unhealthful eating, remain highly prevalent, posing formidable challenges in efforts to improve cardiovascular health. While traditional interventions to promote healthy lifestyles are both costly and effective, wearable trackers, especially Fitbit devices, can provide a low-cost alternative that may effectively help large numbers of individuals become more physically fit and thereby maintain a good health status.

**Objective:**

The objectives of this meta-analysis are (1) to assess the effectiveness of interventions that incorporate a Fitbit device for healthy lifestyle outcomes (eg, steps, moderate-to-vigorous physical activity, and weight) and (2) to identify which additional intervention components or study characteristics are the most effective at improving healthy lifestyle outcomes.

**Methods:**

A systematic review was conducted, searching the following databases from 2007 to 2019: MEDLINE, EMBASE, CINAHL, and CENTRAL (Cochrane). Studies were included if (1) they were randomized controlled trials, (2) the intervention involved the use of a Fitbit device, and (3) the reported outcomes were related to healthy lifestyles. The main outcome measures were related to physical activity, sedentary behavior, and weight. All the studies were assessed for risk of bias using Cochrane criteria. A random-effects meta-analysis was conducted to estimate the treatment effect of interventions that included a Fitbit device compared with a control group. We also conducted subgroup analysis and fuzzy-set qualitative comparative analysis (fsQCA) to further disentangle the effects of intervention components.

**Results:**

Our final sample comprised 41 articles reporting the results of 37 studies. For Fitbit-based interventions, we found a statistically significant increase in daily step count (mean difference [MD] 950.54, 95% CI 475.89-1425.18; *P*<.001) and moderate-to-vigorous physical activity (MD 6.16, 95% CI 2.80-9.51; *P*<.001), a significant decrease in weight (MD −1.48, 95% CI −2.81 to −0.14; *P*=.03), and a nonsignificant decrease in objectively assessed and self-reported sedentary behavior (MD −10.62, 95% CI −35.50 to 14.27; *P*=.40 and standardized MD −0.11, 95% CI −0.48 to 0.26; *P*=.56, respectively). In general, the included studies were at low risk for bias, except for performance bias. Subgroup analysis and fsQCA demonstrated that, in addition to the effects of the Fitbit devices, setting activity goals was the most important intervention component.

**Conclusions:**

The use of Fitbit devices in interventions has the potential to promote healthy lifestyles in terms of physical activity and weight. Fitbit devices may be useful to health professionals for patient monitoring and support.

**Trial Registration:**

PROSPERO International Prospective Register of Systematic Reviews CRD42019145450; https://www.crd.york.ac.uk/prospero/display_record.php?ID=CRD42019145450

## Introduction

Unhealthy behaviors, such as physical inactivity, sedentary lifestyle, and unhealthful eating, remain highly prevalent and pose formidable challenges worldwide [1-4]. These public health problems are associated with mental health problems, cardiovascular diseases, and shorter life expectancies [5-8]. Despite this, only a minority of the population leads healthy lifestyles and meets the general recommendations of 10,000 steps and 20 minutes of moderate-to-vigorous physical activity (MVPA) per day [9-11]. This trend toward physical inactivity affects 23% of the world’s population [12], especially adolescents (81%) and adults (23%), according to the World Health Organization [13]. It also contributes to obesity, with over 650 million people affected worldwide [14,15].

To address the lack of physical activity (PA) and resulting health issues, a substantial amount of research has been dedicated to tracker-based interventions [16-18], which may synergize with the growing use of wearable devices by consumers [19-23]. Among several brands of commercial wearables, Fitbit stands out as one of the most popular commercial wearable activity trackers, with more than 63 million devices sold worldwide in the last 10 years [20] and with an active community [21]. Compared with more traditional PA-related interventions, tracker-based interventions are less resource-intensive and time-consuming, enabling health care providers to cover broader groups of patients [22]. In many cases, these interventions combine components such as individual goal setting, the provision of incentives, social support, and social comparison [11,24-27]. In addition, clinical trials of tracker-based interventions differ in terms of the intervention’s characteristics (eg, time of follow-up and theory-based nature of the intervention) and the populations addressed (eg, pre-existing conditions and age) [16,17,28].

Evidence on the effectiveness of tracker-based interventions is inconclusive [29]. Recent systematic literature reviews and meta-analyses have found that wearable-based interventions have small-to-medium–size effects on PA (ie, steps and MVPA) among adults [16,17,30-32], and there is no evidence of such effects among children and adolescents [18,33]. Furthermore, there is inconclusive evidence that wearable-based interventions are effective at achieving healthier lifestyles through decreases in sedentary behavior [16,34] or through weight loss [28,30,32,35]. The practice of pooling studies on different types of advanced wearable tracking devices with studies on uniaxial pedometers is increasingly being singled out as contributing to the inconclusive nature of the available evidence [36]. Another important explanation for the inconclusive evidence is that current reviews applied only a correlational approach, using meta-analysis tools to analyze the increasing complexity of wearable-based interventions, which typically involved multiple components [16,33]. The growing volume of clinical trials suggests that the effectiveness of tracker-based interventions may depend on complex configurations of interacting and equifinal features [16,31,33]. For instance, such configurations could be the combined provision of tracking devices, social comparison, and gamification in an intervention administered to younger participants, or the combined provision of tracking devices and educational material in a theory-based intervention administered to elderly participants. Current meta-analytic techniques are not suitable for assessing the complex and equifinal effects of complex combinations of intervention components on specific outcomes because the core benefit of the meta-analysis is to reveal the importance of individual variables [37,38]. This review attempts to fill this important gap. It proposes a configurational approach that complements meta-analysis findings by assessing what combination of factors works best.

In short, the purpose of this review was to assess the effects of Fitbit-based interventions, compared with nonwearable control groups, on healthy lifestyle outcomes. A further purpose was to assess the most effective intervention components, beside the Fitbit device, and the study characteristics. We therefore conducted a meta-analysis on the effects of Fitbit-based interventions on a range of healthy lifestyle–related outcomes. We focused on Fitbit devices because they are among the most accurate commercially available wearables [39-43] and are, in some cases, comparable to research-grade monitors [44]. The restriction of this review to Fitbit devices is also due to the fact that this brand is by far the most frequently included in interventional studies found in MEDLINE and ClinicalTrials.gov [45]. This stream of research successfully incorporated Fitbit devices into lifestyle interventions to increase PA, reduce overweight or obesity, and manage chronic diseases such as cancer [46-49]. With this study, we also answer the call to assess the effect of wearables on a broader set of healthy lifestyle–related outcomes [28,31,33,50], including PA-related outcomes, which were the exclusive focus of most previous meta-analyses.

## Methods

### Review Protocol

We conducted and reported this review following the Preferred Reporting Items for Systematic Reviews and Meta-Analyses (PRISMA) guidelines [51,52]. The protocol for this review was registered in the International Prospective Register of Systematic Reviews (PROSPERO; CRD42019145450).

### Search Strategy

The following databases were searched on July 13, 2019: MEDLINE, EMBASE, CINAHL, and Cochrane Controlled Register of Trials (CENTRAL). The search was designed to capture studies involving Fitbit activity tracking devices. No language restrictions were applied. The full search strategy is presented in Multimedia Appendix 1. Electronic searches were supplemented with manual screening of the reference lists of the included articles. We also screened the articles retrieved in prior relevant systematic literature reviews.

### Study Selection

Studies were included if (1) a randomized controlled trial (RCT) design was used, (2) the intervention involved using a Fitbit device to improve PA and/or other health-related outcomes (eg, weight loss), and (3) the study reported outcomes related to healthy lifestyle measures (eg, steps, MVPA, weight, and BMI). Only peer-reviewed journal and conference papers were considered.

Articles were screened in a two-step process. First, all titles and abstracts were examined by one author (MR). Any citations that clearly did not meet the inclusion criteria were excluded. Second, all abstracts and full-text articles were examined independently by two authors (MR and GW). Any disagreements in the selection process were resolved through discussion with a third author (GP or SK).

### Data Extraction

Two authors (MR and GW) independently extracted data from each of the included studies. Discrepancies were resolved through discussion and meetings with a third author (SK). The following data were recorded: author; year; country in which the study was conducted; study design; participant characteristics; sample size; intervention description (eg, intervention duration, model of Fitbit used, intervention components, and theoretical basis); control or comparator group description; primary and secondary outcomes (including method of assessment); and main study results, including relevant subgroup analyses. Within- and between-group quantitative findings (eg, mean differences and significance) were summarized for each study.

### Risk of Bias Assessment

Two authors (MR and SK) assessed each study for risk of bias using the Cochrane Collaboration seven domain-based criteria as follows [53,54]: sequence generation (selection bias), allocation concealment (selection bias), blinding of participants and personnel (performance bias), blinding of outcome assessment (detection bias), incomplete outcome data (attrition bias), selective outcome reporting (reporting bias), and other (other bias). Each criterion was scored as low, unclear, or high risk. Disagreements were resolved through discussion.

### Data Analysis

Because of the variability of the included studies, random-effects meta-analyses [55] were performed on the following most frequently reported outcomes using Review Manager (RevMan) [53]: daily step count, MVPA (min/day), sedentary behavior (min/day), and weight (kg). Data were converted to the same units in order to compare the findings. For instance, weekly step counts were divided by 7, whereas data presented as hours per day were divided by 60 to obtain minutes per day. Studies that included multiple intervention groups (eg, group A: Fitbit alone; group B: Fitbit + text messages) were entered once in the meta-analysis to avoid double counting the control group. We retained the group with the fewest interventional components (eg, Fitbit alone) that matched our initial review objectives, and excluded the other intervention group from the analysis [56]. In studies including a control group that received a delayed intervention, we took into consideration the reported outcomes before the control group received the intervention. For instance, Li et al [57] reported PA changes resulting from a 2-month intervention during which the first half of the intervention was exclusively administered to the intervention group. In this case, we considered the reported outcomes at 1 month. Data presented as mean, standard error (SE), or 95% CI were converted to SD using the RevMan calculator. We analyzed objective and self-reported measures separately because self-reported outcomes have a higher risk of over-estimation [58,59]. Although some studies used different actigraph devices, PA measures (eg, steps and intensity of activity) were reported similarly. In this case, meta-analytic evaluations of the pooled mean difference (MD) in steps/day, min/day of MVPA, min/day of sedentary behavior, and weight (kg) between the intervention and comparison groups for the objective outcome measures were calculated using mean changes or postintervention data, depending on what the authors had reported. When any relevant data were missing, mean or mean changes and corresponding SD were requested from the corresponding author. Authors of studies that presented data in a graphical format were contacted to obtain the exact values. Forest plots for steps were drawn using GraphPad Prism software (GraphPad Software Inc), because the scale in Review Manager has a limit of 1000 points.

In the presence of high statistical heterogeneity in the outcomes reported in the meta-analysis (*I^2^*>50%), we conducted subgroup analysis and fuzzy-set qualitative comparative analysis (fsQCA) to explore potential reasons for this heterogeneity and proposed several explanatory hypotheses in our protocol. We assumed that the treatment effect was influenced by (1) a theoretically grounded treatment, (2) the duration of the treatment, and/or (3) the subject’s health condition. We considered these subgroup analyses because there is some evidence that theory-based interventions are more effective [60-62] and that the effects of wearable activity trackers may not be sustainable over time, favoring short interventions. Individuals with chronic conditions may respond to the treatment differently from healthy people because the treatment allows chronic patients to live better with their health conditions, while healthy individuals may consider it as a tool to prevent health problems. We also conducted post-hoc subgroup analyses between studies reporting postintervention values and those reporting mean changes from baseline values to explore whether there are any significant differences between the two reporting methods that may introduce bias in the principal meta-analyses. In addition, we identified a very small number of studies (n=2) that reported significant differences between groups at baseline, and we conducted sensitivity analyses to assess whether these trials made any difference to the results of the principal meta-analyses. Finally, we assessed publication bias using funnel plot analysis for each outcome included in our meta-analysis. To permit publication bias assessment, funnel plot analysis can be conducted only on outcomes that include 10 or more studies [63].

The fsQCA method can identify complex (ie, nonlinear and nonadditive) causal patterns [37]. It is especially appropriate when dealing with complex interventions [38]. FsQCA considers the necessity and sufficiency of conditions for an outcome. In our case, we included the following two types of conditions: the main intervention components that were present in the included studies and the same study characteristics as in the subgroup analysis (ie, grounded in a theory, length of the intervention, and chronic disease in the subjects). We focused on a range of outcomes (ie, steps, MVPA, sedentary behaviors, and weight) that are important components of a heathy lifestyle [64-66]. FsQCA is an analytical method that allows us to assess which configurations of conditions or factors (ie, intervention components and study characteristics) lead to successful outcomes. Multimedia Appendix 2 provides a detailed explanation of how fsQCA was applied.

## Results

### Study Selection

**Figure 1:**
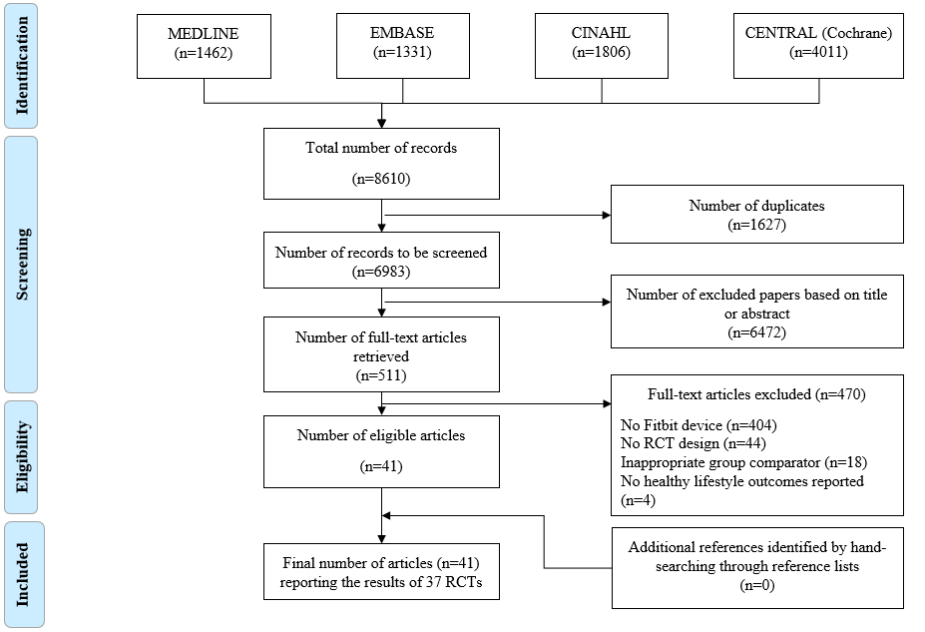
Flow diagram. RCT: randomized controlled trial.

In total, 8610 articles were retrieved using the search strategy. A total of 1627 duplicates were removed, and 6983 records were screened by title and abstract, with 6472 records removed after the application of our selection criteria. The remaining 511 articles were retrieved and assessed for eligibility based on the full text. In total, 41 articles were included, reporting the results of 37 RCTs (Figure 1) [48,49,57,67-104]. All 41 articles are described in detail, based on study design and PICO (population, intervention, comparison, outcome) characteristics (Multimedia Appendix 3).

### Study Characteristics

The studies were conducted in North America (27/37, 73%), Europe (4/37, 11%), Australia (4/37, 11%), and Asia (2/37, 5%). Approximately two thirds (24/37, 65%) of the studies were conducted in the United States.

**Figure 2:**
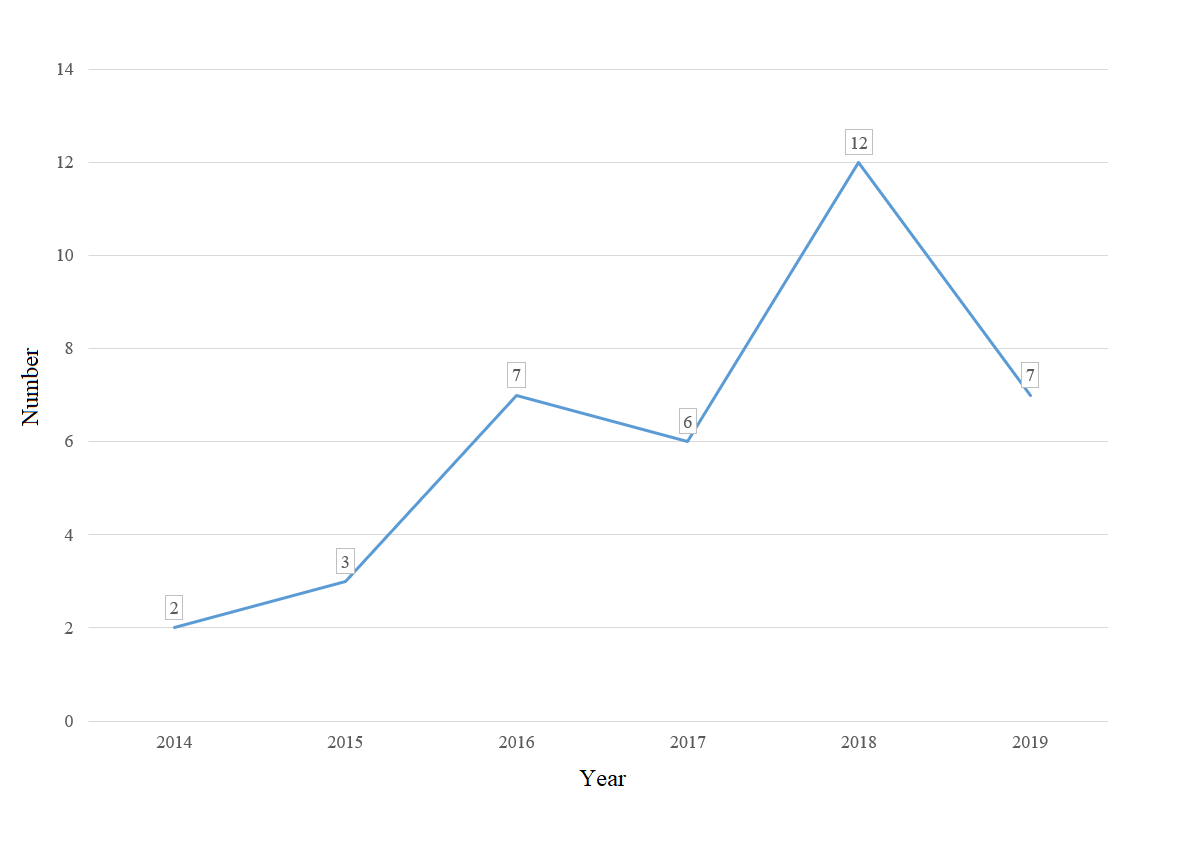
Number of Fitbit-based randomized controlled trials published each year.

The volume of Fitbit-based intervention studies has steadily increased since the first one was published in 2014. The number of articles published per year increased to 12 RCTs in 2018, with most of the studies (25/37, 68%) published in the last 3 years (Figure 2).

Most of the studies (35/37, 95%) were parallel RCTs, while two studies used a factorial design and a cross-over design, respectively [79,101]. Among the parallel RCTs, 27 featured two arms [48,57,67-73,76,78,81,83-85,88-93,95,96,99,102-104], while eight featured multiple intervention arms [49,77,82,86,87,94,97,98]. For instance, Finkelstein et al [82] conducted an RCT with four groups; three groups receiving a Fitbit device and the other receiving interventional components differing in terms of the type of financial incentives.

The follow-up duration ranged from 1 week [79] to 1 year [82,90,95,101], with most studies lasting less than 5 months (20/37, 54%).

To summarize, the majority of the studies were conducted in Western countries, adopted a parallel RCT research design, and lasted less than 5 months.

### Population Characteristics

A total of 3779 participants were included, with a mean of 102 participants per study (median 68) and range from 16 [98] to 800 participants [82] per study.

Virtually all studies focused on individuals over the age of 18 years (36/37, 97%). Only one study included adolescents [93]. Thirteen studies included young adults (age 18-43.9 years) [73,77,79,81-83,86,90,91,94,97,99,102], 17 studies included middle-aged adults (age 44-64.9 years) [48,49,57,67-72,78,84,87-89,96,103,104], and six studies included older adults (age ≥65 years) [76,85,92,95,98,101].

Concerning the main characteristics of the targeted population, most studies (21/37, 57%) reported that their participants had a particular condition or were at risk. This included patients with cardiovascular risks [70,98] and patients having chronic diseases like chronic obstructive pulmonary disease [85] or cardiometabolic diseases [69]. In the remaining studies, participants were selected based on their personal or professional status (eg, employees or students, such as medical students) (8/37, 22%) or their health status (eg, postoperation and cancer survivor) (8/37, 22%). Only one study focused specifically on healthy subjects [81]. Table 1 summarizes the main characteristics of each targeted population.

**Table 1:** Characteristics of the participants.

Main characteristic and specific characteristics		Reference
**Having a condition or being at risk**		
	Overweight/obese	[48,71,83,97,104]
	Sedentary	[68,94,99,101]
	Arthritis	[57,87,89]
	Cardiovascular risks	[70,98]
	Diabetes	[73,88]
	Cardiometabolic diseases	[69]
	Chronic low back pain	[67]
	Chronic obstructive pulmonary disease	[85]
	Prediabetes	[49]
	With low ankle brachial index	[92]
**Personal/professional status**		
	Students	[77,79,86,90,91,102]
	Community-dwelling people	[95]
	Employees	[82]
**Health status**		
	Postoperation/posttreatment	[76,78,96,103]
	Cancer survivor	[72,84,93]
	Healthy	[81]

To summarize, the included studies had sample sizes of less than 100 adult subjects, who mainly had a chronic condition or were at risk of having one.

### Intervention Characteristics

The intervention components were highly heterogeneous (Multimedia Appendix 4). Four studies included at least one interventional arm involving only the use of a Fitbit device [77,79,81,91], while the majority (35/37, 95%) included at least one interventional arm involving a comprehensive program for improving PA and facilitating weight loss. For instance, the components of the intervention in the study by Amorim et al [67] included an information booklet on PA and sedentary behavior, a tailored PA plan, a face-to-face coaching session, 12 phone calls from a health coach, weekly personalized messages to encourage participants to achieve their goals, and a Fitbit device with its web-interfaced IMPACT mobile app. In addition to the wearable device, other intervention components included the use of an app or a website (sometimes different from the app provided by the device manufacturer), goal setting and prescription, messaging, education, counseling and feedback, social support, financial incentives, and the provision of another device (Multimedia Appendix 4). Further details on these intervention components are provided in Multimedia Appendix 5.

As expected, there was a wide variety of Fitbit devices used in the included studies. Most of them (17/37, 46%) used clip-on devices such as Fitbit Zip [76,79,82,83,87,88,90,92,96-98], Fitbit One [48,68,81,84,94], and Fitbit Ultra [85]. Seven studies did not specify which model was used [49,67,69,70,95,101,102]. The remaining studies used wrist-worn devices, such a Fitbit Flex [57,71,73,77,86,89,91,93,103,104] and Fitbit Charge [72,78,99]. Use patterns with these devices were mentioned in 35% (13/37) of the studies. Beside reporting use or wear durations [49,88,103], the studies indicated Fitbit use as the frequency at which the subjects wore the device [48,67,93,95,96] and the number of subjects having Fitbit measurements [73,82,87,90,92]. This information was mainly assessed using device data or it was self-reported.

In summary, most of the interventions did not rely on theory and used Fitbit Zip or Flex tracking devices for interventional purposes along with several other components commonly related to goal setting and education.

### Control Group Characteristics

Most studies included some form of PA or other healthy lifestyle education component [49,67,68,70,72,73,82,84,86,87,90, 99,103]. Other studies involved usual care [48,76,78,83,85,88,93,96-98], financial incentives [68,82], blinded wearables [94,101], or no intervention at all [77,79,81,91,92]. Participants in some control groups were put on a waiting list to receive the same intervention following a delay [57,69,71,89]. Two studies included a control group to which overlapping intervention components were allocated [102,104]. For example, in the RCT published by Vandelanotte et al [104], the comparison group received the same intervention components as the interventional arm, except for the Fitbit Flex.

### Study Outcomes

Taken together, the studies in our sample reported a wide range of outcomes that can be classified into the following several categories: PA-related outcomes (eg, steps, MVPA, and light PA), weight-related outcomes (eg, weight and BMI), sedentary behavior outcomes, dietary intake-related outcomes, oxygen uptake outcomes, sleep-related outcomes, quality of life, self-efficacy, and overall health (Multimedia Appendix 6). While steps were usually reported in steps/day, MVPA was reported in different ways, such as min/day, days/week, metabolic equivalent (MET)-min/week, MET-min/week in 10-minute bouts, etc. This was also the case for sedentary behavior outcomes. These behaviors were mostly reported in min/day [68,89,93,103,104], while few studies reported them as prolonged sedentary 30-minute bouts (%/day) [100] or sedentary activity (<5000 steps per day, %) [87].

Furthermore, the studies featured several ways of measuring the reported outcomes, especially steps, MVPA, and sedentary behavior. For steps, eight studies (22%) used the intervention Fitbit device as a measurement tool [77-79,86,88,90,94,102]. This outcome was also frequently (8/37, 22%) assessed using a research-grade accelerometer (ie, actigraph) [67,68,72,76,82,96,99,103]. Other studies reported measures taken with a SenseWear Mini device [89], a Jawbone Up wearable [87], a Yamax pedometer [73], and a Dynaport MoveMonitor device [85]. One study did not report how the step counts were assessed [91]. MVPA outcomes were mostly measured using a research-grade accelerometer (13/37, 35%) [48,67,68,71,72,76,82,84,93,95,98,99,103], and, less often, they were self-reported (5/37, 14%) [67,79,86,88,104] or relied on the use of a SenseWear Mini device (2/37, 5%) [57,89]. One study used the intervention Fitbit device to measure MVPA [78]. Finally, research-grade accelerometers [68,82,93,98,103] and self-reported measures [82,104] were used to assess sedentary behaviors. Two studies measured sedentary behaviors using a SenseWear Mini device [57,89] and one study used a Jawbone Up wearable [87].

To summarize, the available evidence was primarily based on studies that reported PA outcomes, such as steps and MVPA, mostly measured with an actigraph.

### Risk of Bias

**Figure 3:**
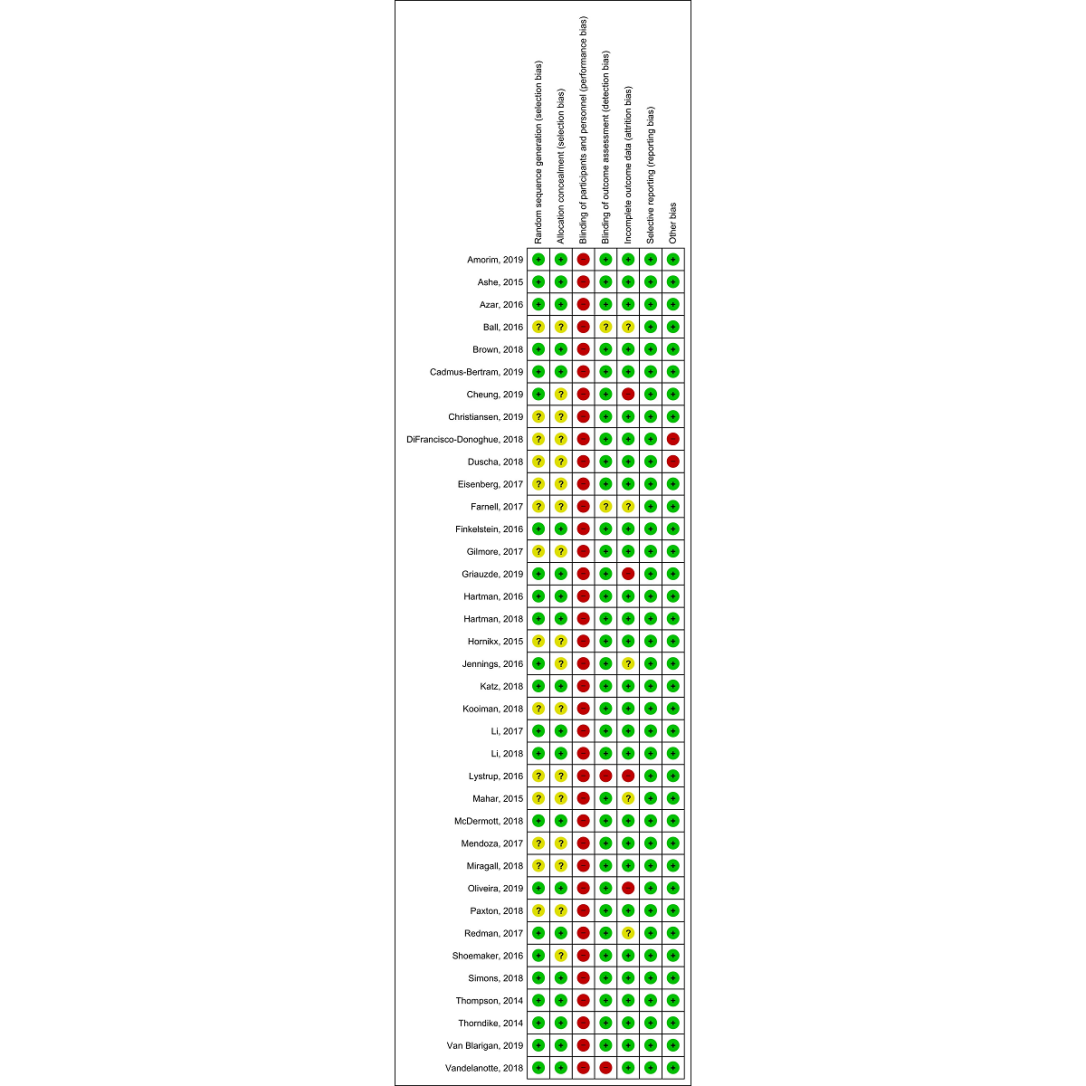
Risk of bias summary for each included study.

Risk-of-bias judgements are presented in Figure 3. Random sequence generation was assessed as being at low risk of bias (23/37, 62%) or unclear (14/37, 38%) in the included studies. Allocation concealment was assessed as being at low risk of bias (20/37, 54.05%) or unclear (17/37, 45.95%) in the included studies. The blinding of participants and personnel was assessed as being at high risk of bias for all the studies because the nature of the intervention and control conditions rendered blinding not feasible. Blinding of outcome assessment was evaluated only in terms of the primary outcome of interest, and was reported as low in cases where it was measured objectively. This was assessed as being at high risk of bias in two studies because of the use of subjective measures [104] and because the authors clearly mentioned that it was an unblinded clinical trial [90]. The management of incomplete outcome data was assessed as being at high risk of bias in four studies [49,73,90,95]. The reasons were high attrition (more than 25%) [95], high imbalance in loss of follow-up between groups [49], limitation of the analysis to subjects who had completed a running event [90], and long periods during the intervention when the activity monitor was not worn [73]. Selective reporting was assessed as being at low risk of bias for all the included studies. Finally, two studies were assessed as having a high risk for other bias because of conflicts of interest declared by the authors [78] and significant differences between the groups at baseline [77]. All the reasons in the risk of bias assessment can be found in Multimedia Appendix 7.

### Meta-Analysis Results

We proceed by presenting the main results of the meta-analysis. Detailed information on all subgroup meta-analyses can be found in Multimedia Appendix 8.

#### Steps

**Figure 4:**
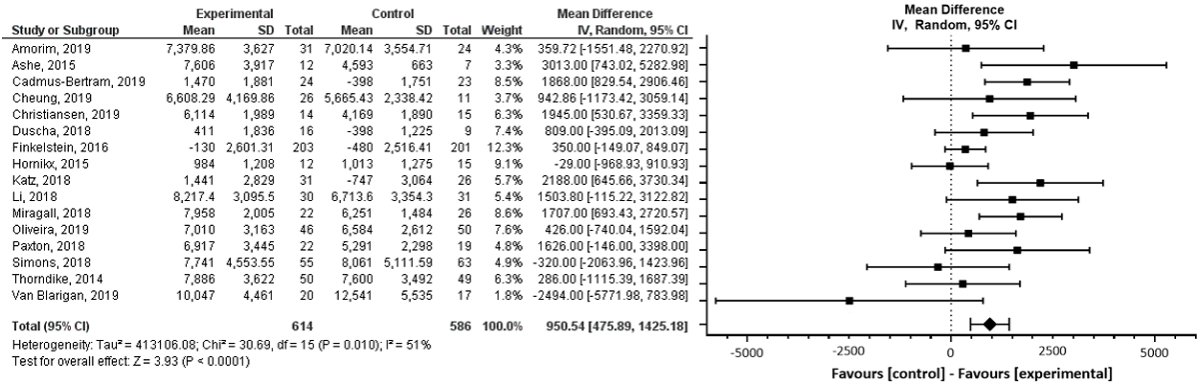
Forest plot of mean difference in steps per day in studies comparing an intervention that included a Fitbit device with a control group that did not utilize such a device.

Of the 37 studies included in the review, 23 reported an outcome related to steps [67,68,72,73,76-79,82,85-92,94-96,99,102,103] and 16 reported this outcome in a way we could use in the meta-analysis [67,68,72,73,76,78,82,85,87,89,94-96,99,102, 103]. On average, Fitbit-based interventions were associated with a statistically significant increase in the number of daily steps when compared with the control groups (MD 950.54, 95% CI 475.89-1425.18; *P*<.001; Figure 4) across most of the studies in the meta-analysis (13/16, 81%). Three studies showed a decrease in the number of steps [85,99,103]. There was high heterogeneity between study results in terms of the magnitude of the effects (*I^2^*=51%).

Subgroup analyses showed that the length of the intervention, the subjects’ health condition, and theory-based interventions did not have significant impacts on the number of steps (*P*=.97, *P*=.32, and *P*=.86, respectively). When we categorized studies by reporting method, we found no evidence of clinically or statistically significant (*P*=.86) differences between studies that reported postintervention data and those that reported mean change from baseline data (Multimedia Appendix 8). A sensitivity analysis excluding the study by Van Blarigan et al [103], in which there was an imbalance in steps between the control and intervention groups at baseline, showed similar results (*P*=.87). Funnel plot analysis showed no evidence of publication bias (Multimedia Appendix 9).

#### MVPA

**Figure 5:**
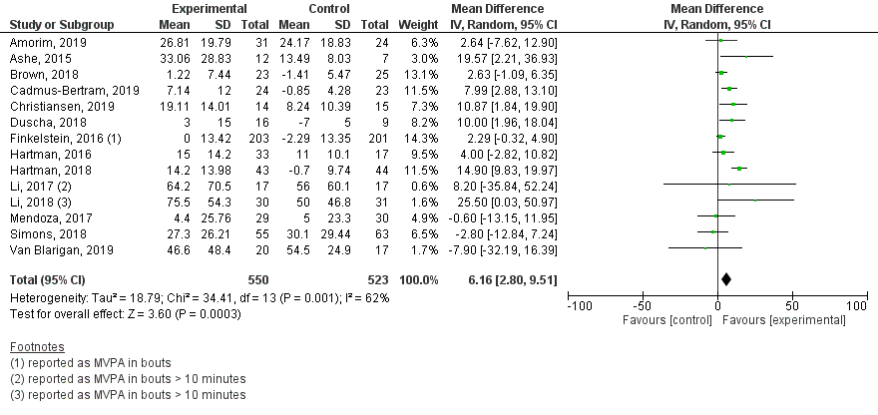
Forest plot of mean difference in moderate-to-vigorous physical activity (MVPA; min/day) in studies comparing an intervention that included a Fitbit device with a control group that did not utilize such a device.

Of the 37 studies included in the review, 21 reported MVPA [48,57,67,68,71-73,76,78,79,82,84,86,88,89,93,95,98,99,103,104] and 14 studies reported this outcome in a way that we could use in the meta-analysis [48,57,67,68,71,72,76,78,82,84,89, 93,99,103]. There was a statistically significant increase in minutes per day spent on MVPA in the Fitbit-based interventions compared with the comparison groups (MD 6.16, 95% CI 2.80-9.51; *P*<.001; Figure 5). The study results featured high heterogeneity (*I^2^*=62%).

The subgroup analyses showed that only theory-based interventions had a significant impact on MVPA (*P*<.001), in contrast to the findings for length of follow-up and subjects’ health condition (*P*=.28 and *P*=.29, respectively). When we categorized studies by reporting method, we found no evidence of clinically or statistically significant (*P*=.93) differences between studies that reported postintervention data and those that reported mean change from baseline data (Multimedia Appendix 8). A sensitivity analysis excluding the study by Van Blarigan et al. [103], in which there was an imbalance in MVPA between the control and intervention groups at baseline, showed no significant (*P*=.92) impact on the overall effect size. There was no evidence of publication bias (Multimedia Appendix 9).

#### Weight

**Figure 6:**
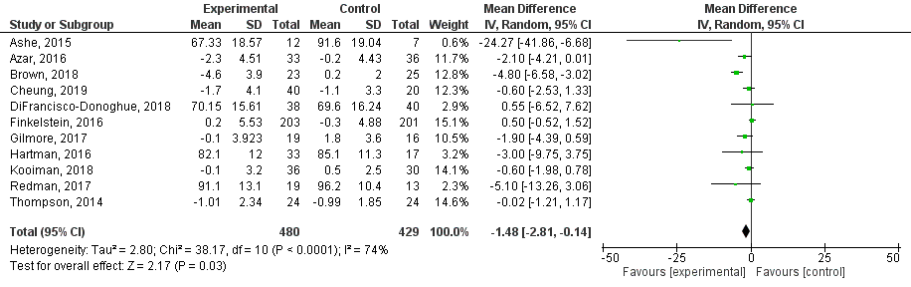
Forest plot of mean difference in weight (kg) in studies comparing an intervention that included a Fitbit device with a control group that did not utilize such a device.

Of the 37 studies included in the review, 15 reported an outcome related to weight [48,68-71,73,77,82-84,88,97,101,102,104] and 11 studies reported this outcome in a way that we could use in the meta-analysis [48,68,69,71,73,77,82,83,88,97,101]. Weight was measured by the research team. A random-effects meta-analysis using MD performed on the 11 studies showed a significant decrease in weight in the Fitbit-based interventions compared with the control groups (MD −1.48, 95% CI −2.81 to −0.14; *P*=.03; Figure 6). Heterogeneity was high (*I^2^*=74%).

The subgroup analyses showed that only subjects’ health condition had a significant impact on weight (*P*=.009), in contrast to the findings for length of follow-up and theory-based interventions (*P*=.26 and *P*=.31, respectively). When we categorized studies by reporting method, we found no evidence of clinically or statistically significant (*P*=.30) differences between studies that reported postintervention data and those that reported mean change from baseline data (Multimedia Appendix 8). A sensitivity analysis excluding the study by Ashe et al. [68], in which there was a weight difference between the control and intervention groups at baseline, showed similar results (*P*=.86). Publication bias was not detected for this outcome (Multimedia Appendix 9).

#### Sedentary Behaviors

**Figure 7:**
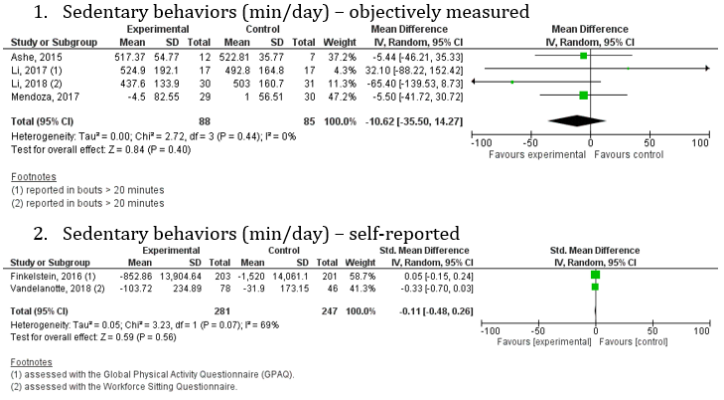
Forest plot of sedentary behaviors (min/day) in studies comparing an intervention that included a Fitbit device with a control group that did not utilize such a device.

Of the 37 studies included in the review, 10 reported an outcome related to sedentary behaviors [57,68,82,87,89,93,98,103,104] and six reported this outcome in a way we could use in the meta-analysis [57,68,82,89,93,104]. Sedentary behavior was measured objectively, except in two studies [82,104] that utilized a self-reported questionnaire to obtain daily sitting time or sedentary behavior. A random-effects meta-analysis was performed on four studies that objectively measured sedentary behavior using MD. The other two were assessed using standardized mean difference (SMD). For objective measures, there was a nonsignificant decrease in sedentary behavior following the intervention compared with the control comparator (MD −10.62, 95% CI −35.50 to 14.27; *P*=.40; Figure 7) across most of the studies in the meta-analysis (3/4, 75%), with a low level of heterogeneity (*I^2^*=0%). For self-reported measures, there was a nonsignificant decrease in sedentary behavior following the intervention compared with the control comparator (SMD −0.11, 95% CI −0.48 to 0.26; *P*=.56; Figure 7), with a high level of heterogeneity (*I^2^*=69%). Given the small sample size, no subgroup analysis could be conducted.

### FsQCA Results

We conducted all our analyses using a frequency cut-off of 2 per relevant configuration and a minimum raw consistency of 0.8, combined with a minimum proportional reduction in consistency (PRI consistency) of 0.6. This is consistent with the report by Rihoux and Ragin [37].

**Figure 8:**
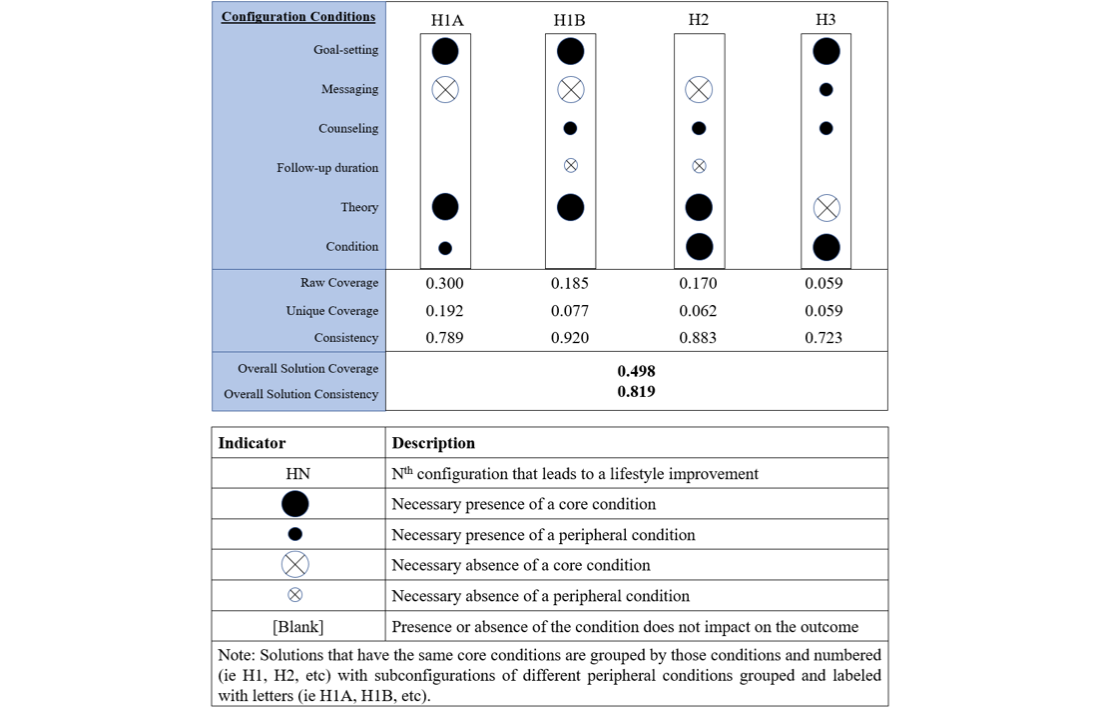
Fuzzy-set qualitative comparative analysis configurations associated with improvements in healthy lifestyle outcomes.

First, we conducted a separate analysis of each condition (intervention components and study characteristics) owing to the lack of cases for joint analyses with all conditions (Multimedia Appendix 2). Configuration analyses by intervention components covered a relatively high number of observed cases (35.14%), whereas the study characteristics did not cover enough cases to analyze them further (no configuration had a high enough raw coverage to conduct an analysis) (Multimedia Appendix 10 and Multimedia Appendix 11). Based on these results and the meta-analysis results, we then combined the following conditions: goal setting, messaging, counseling, length of intervention (named “follow-up duration” below, see details in Multimedia Appendix 2), theory-based interventions, and subjects’ conditions. Figure 8 depicts the fsQCA results using the notation system from Ragin and Fiss [105].

Figure 8 shows two measures that validated the solutions (consistency and coverage). HN indicates the Nth configuration that leads to a lifestyle improvement. Initially, overall solution consistency measures the degree to which all configurations together consistently result in an improvement in healthy lifestyle outcomes. In our case, overall consistency was 0.819, which is above the usually acceptable level of 0.80 [106]. Raw coverage shows empirical relevance and effectiveness of the solution for the outcome, although higher coverage does not necessarily mean theoretical importance [106]. Thus, there are multiple paths to better outcomes for individuals with pre-existing conditions, but the most effective one (albeit for the short- or long-term interventions) centers on theory-based interventions with goal-setting but without messaging (H1A). In fact, in all but one configuration, goal setting was a condition for better outcomes, with or without either messaging or counselling. To differentiate between the numerous outcomes, Table 2 presents these results according to intervention length and subjects’ health condition.

**Table 2:** Configurations leading to better lifestyle outcomes depending on the intervention length and subjects’ health condition.

Intervention length and subjects’ health condition	Follow-up duration (long follow-up duration)^a,b^	~Follow-up duration (short follow-up duration)^b,c^
Condition (subjects with a chronic condition)^a^	H1A H3	H1A H1B H2 H3
~Condition (healthy subjects)^c^	N/A^d^	H1B

^a^Configuration element that needs to be present for an improved lifestyle outcome.

^b^HN: Nth configuration that leads to a lifestyle improvement.

^c^Configuration element that needs to be absent for an improved lifestyle outcome.

^d^Configuration absent in our sample

Table 2 suggests that there are more paths to success for people with pre-existing conditions and none for people without pre-existing conditions who receive long-term interventions. Although it may appear reasonable to assume that the additive effects of more intervention components lead to better outcomes [16], this is not confirmed by our analyses. The configuration with all the intervention components (H3) is not the most effective (it has the lowest raw coverage).

## Discussion

### Principal Findings

Our review summarizes the results of interventions that included a Fitbit wearable device to improve healthy lifestyle outcomes. Our meta-analysis results showed improvements in two PA outcomes, namely, steps and MVPA. Fitbit-based interventions also resulted in weight loss. However, sedentary behaviors did not improve, regardless of whether they were measured objectively or self-reported. These results are in line with prior reviews on wearables that showed no change in sedentary behaviors [16], and an improvement in PA outcomes [16-18,28,30-32,35,36,107] and weight loss [28,32,35,108]. However, the current evidence is mostly representative for adults and older subjects. The lack of studies focused on vulnerable populations, such as youth and adolescents, may be explained by some of the challenges faced when recruiting subjects from these populations and conducting RCTs (eg, securing consent from the legal guardian).

Considering the overall high heterogeneity in our meta-analysis results, we followed well-established guidelines to investigate them further [109]. We did this by applying the following two different methodologies: subgroup analysis and fsQCA. The former allows us to answer specific questions about a particular aspect of the study (eg, length and theory-based approach) and types of intervention components or patient characteristics (eg, age and condition), whereas the latter emphasizes the configuration of factors (eg, intervention components and study characteristics). Subgroup analyses showed significant (*P*<.001) improvements in MVPA among nontheory-based interventions. This contradicts the results of McCullough et al [62], who found that theory-based interventions are more effective. We also observed that weight loss was more significant (*P*=.009) among patients with chronic conditions. Further analyses using fsQCA uncovered additional interesting results. We found that both theory- and nontheory-based interventions contributed, but it would appear that this factor depends on specific conditions in order to lead to effective interventions. In all but one configuration, goal setting was a condition for better outcomes, with or without either messaging or counselling. This is true for lifestyle outcomes and weight outcomes (Multimedia Appendix 12, Multimedia Appendix 13, and Multimedia Appendix 14). Interestingly, neither the presence nor the absence of goal setting improved PA. Instead, the absence of messaging and/or absence of counselling were the most relevant conditions for improving PA. This was not expected, because previous studies have found these two intervention components effective on their own [110-112]. When combining messaging and counselling with other conditions, it appears that they are outweighed by other factors, such as education and subjects’ health conditions. Future research is thus needed to investigate what mitigates the contributions made by messaging and counselling in interventions to improve PA. Of interest is the lack of better outcomes in long-term studies among participants without pre-existing conditions, and the more limited number of paths to success for longer term participants with health conditions (Table 2). This either suggests that there were fewer such studies or that it is difficult for subjects to maintain their focus in order to achieve long-term results. The latter can easily be understood for weight outcomes since early weight loss is rapid and then tends to stall on a plateau for an extended period of time [113].

Moreover, we observed that better results were achieved with a combination of study and intervention components as compared to intervention components alone. In other words, there is complex causality at play, whereby individual and study characteristics are also important criteria to consider when evaluating the effectiveness of an intervention. This is coherent with the idea that technological interventions may not produce similar effects in different individuals [114]. This means that studies that do not adequately consider study characteristics and participant profiles may produce invalid conclusions regarding the effectiveness of an intervention.

Furthermore, we found that goal setting was the most promising intervention component, whereas messaging seemed to be mostly ineffective in complex interventions. These results can be illustrated with two studies from our sample. Amorim et al [67] found that setting goals increased outcomes related to steps and other activities, such as yoga and swimming. In contrast, Cheung et al [73] concluded that “the vast majority (of participants) found that the messages (on PA, nutrition, and general health and motherhood information and education) were helpful, although the reported effects on diet and PA were more modest.” Finally, the length of an intervention does not appear to be relevant, because it was not significant in the subgroup analysis (*P*=.97 for steps, *P*=.28 for MVPA, and *P*=.26 for weight), and the most dominant configuration (H1A) was not affected by this factor.

### Strengths and Limitations

Prior systematic literature reviews are limited by the quality and nature of the studies included. To avoid this, we included only studies featuring a Fitbit device as an interventional component. Despite this, and much like other reviews, the studies in our sample involved very heterogenous interventions, rendering assessments of the effects of Fitbit interventions more difficult. However, a thorough systematic and transparent methodology was followed [115,116], and the use of meta-analysis tools and fsQCA allowed us to interpret the combined effects of Fitbit devices with the other interventional components and subject characteristics. Using these two methodologies enabled us to provide a fine-grained picture of the effectiveness of Fitbit-based interventions. Despite promising findings, applications of QCA in systematic reviews are still relatively new, especially in digital health research [38,117]. We hope that this review will help promote its application in future studies.

The results of our review must be interpreted in light of some limitations. First, even though we included a large range of outcomes, we could not assess the effectiveness of the interventions on each of them. Rather, we limited our analyses to PA outcomes, sedentary behaviors, and weight. While most of the articles in our sample examined well-studied outcomes (eg, steps and MVPA), other studies reported less common ones, such as cognition and dietary intake. Second, we could not assess the effectiveness of the Fitbit device itself on healthy lifestyle outcomes. This was due to (1) the high complexity and variety of the interventions and (2) the number of studies that did not describe the Fitbit artifact. Indeed, the studies in our sample rarely described the wearable and ignored its specific features. As shown by Lyons et al [118] and Mercer et al [119], each device incorporates different behavioral change techniques that are linked to one or several features of the wearable, so providing a description of the features of the device and the associated app (if used) is essential for future research. Consideration of the features of these devices is also important because James et al [120] found that each set of features does not impact health outcomes equally. This study suggests that only the social interaction and data management features of activity trackers help improve well-being outcomes. Finally, we could not assess the effects of the different behavioral change techniques incorporated in the Fitbit devices as proposed in our protocol owing to high heterogeneity and the lack of information reported in the included studies. This gave us the opportunity to apply a new methodology (fsQCA) that is relevant to complex interventions in order to determine the most important conditions for Fitbit-based interventions.

### Conclusions and Future Research

Fitbit devices, included either as the primary component of an intervention or as part of a more comprehensive and complex intervention, have the potential to improve healthy lifestyle behaviors and, in particular, PA. The included studies encompassed mainly adult populations with pre-existing chronic conditions. Although the findings were not significant in all the RCTs, short-term interventions utilizing a Fitbit device generally resulted in improvements in terms of a healthy lifestyle. In addition to these activity trackers, we showed that goal setting is an effective complementary interventional component over the short and long term. Further research would be beneficial to determine the effect of a Fitbit device independent of other interventional components, as would investigations into the cost-effectiveness of Fitbit-based interventions. Given the potential associated with the use of PA trackers, further studies investigating their long-term use would be useful to guide potential clinical applications and future recommendations. Finally, future research could also focus on the effectiveness of such interventions in healthy subjects and consider subjective outcomes, such as psychological health and personal motivation.

## Appendix

Multimedia Appendix 1 Search queries.

Multimedia Appendix 2 Fuzzy-set qualitative comparative analysis methodology details.

Multimedia Appendix 3 PICO table.

Multimedia Appendix 4 Characteristics of the intervention components in the included studies.

Multimedia Appendix 5 Intervention component details in the included studies.

Multimedia Appendix 6 Reported outcomes in the included studies.

Multimedia Appendix 7 Risk of bias assessment results.

Multimedia Appendix 8 Subgroup analysis results.

Multimedia Appendix 9 Funnel plots for publication bias.

Multimedia Appendix 10 Fuzzy-set qualitative comparative analysis truth tables for main results.

Multimedia Appendix 11 Fuzzy-set qualitative comparative analysis configuration charts for main results.

Multimedia Appendix 12 Fuzzy-set qualitative comparative analysis truth tables for secondary analysis.

Multimedia Appendix 13 Fuzzy-set qualitative comparative analysis configuration charts for secondary analysis.

Multimedia Appendix 14 Fuzzy-set qualitative comparative analysis dataset.

## Abbreviations

FsQCA: fuzzy-set qualitative comparative analysis
MD: mean difference
MET: metabolic equivalent
MVPA: moderate-to-vigorous physical activity
PA: physical activity
RCT: randomized controlled trial
SMD: standardized mean difference
