# Supplementary material for: Fitbit-Based Interventions for Healthy Lifestyle Outcomes: Systematic Review and Meta-Analysis
Source: J Med Internet Res. 2020 Oct 12;22(10):e23954. doi: 10.2196/23954 (PMC7589007; doi:10.2196/23954)
Supplement: Multimedia Appendix 1 [file jmir_v22i10e23954_app1.docx]

**CENTRAL Cochrane:**

#1 MeSH descriptor: [Fitness Trackers] explode all trees

#2 fitness track*:ti,ab,kw

#3 activity tracker*:ti,ab,kw

#4 activity monitor:ti,ab,kw

#5 activity self-monitor*:ti,ab,kw

#6 activity sensor:ti,ab,kw

#7 activity sensors:ti,ab,kw

#8 wrist-worn device:ti,ab,kw

#9 wrist-worn devices:ti,ab,kw

#10 wristband accelerometer:ti,ab,kw

#11 Self-track*:ti,ab,kw

#12 MeSH descriptor: [Wearable Electronic Devices] this term only

#13 wearable electronic device*:ti,ab,kw

#14 wearables:ti,ab,kw

#15 wearable track*:ti,ab,kw

#16 wearable technolog*:ti,ab,kw

#17 wearable sensor*:ti,ab,kw

#18 wearable device*:ti,ab,kw

#19 wearable activity:ti,ab,kw

#20 Fitbit:ti,ab,kw

#21 #1 or #2 or #3 or #4 or #5 or #6 or #7 or #8 or #9 or #10 or #11 or #13 or #14 or #15 or #16 or #17 or #18 or #19 or #20 with Publication Year from 2007 to present, in Trials

**CINAHL:**

| S10 | S1 AND S9 |
| --- | --- |
| S9 | S2 OR S3 OR S4 OR S5 OR S6 OR S8 |
| S8 | TX allocat* random* |
| S7 | (MH "Quantitative Studies") |
| S6 | TX random* allocat* |
| S5 | TX randomi* control* trial* |
| S4 | TX ((singl* n1 blind*) or (singl* n1 mask*) ) or TX ( (doubl* n1 blind*) or (doubl* n1 mask*) ) or TX ( (tripl* n1 blind*) or (tripl* n1 mask*) ) or TX ( (trebl* n1 blind*) or (trebl* n1 mask*) ) |
| S3 | TX clinic* n1 trial* |
| S2 | (MH "Clinical Trials+") OR (MH "Crossover Design") OR PT Clinical Trial OR (MH "Random Assignment") |
| S1 | MH "Fitness Trackers" OR MH "Wearable Sensors+" OR TX ( "fitness track*" OR "activity track*" OR "activity monitor*" OR "activity self-monitor*" OR "activity sensor" OR "activity sensors" OR "wrist-worn device" OR "wrist-worn devices" OR "sleep track*" OR "wearable electronic device*" OR wearable* OR "wearable track*" OR "wearable technolog*" OR "wearable sensor*" OR "wearable device*" OR "wearable activity" OR Fitbit) |

**EMBASE:**

#42. #41 AND (2007:py OR 2008:py OR 2009:py OR 2010:py OR 2011:py OR 2012:py OR 2013:py OR 2014:py OR 2015:py OR 2016:py OR 2017:py OR 2018:py OR 2019:py)

#41. #38 AND #40

#40. #39 NOT #37

#39. #23 OR #24 OR #25 OR #26 OR #27 OR #28 OR #29 OR #30 OR #31 OR #32 OR #33 OR #34 OR #35 OR #36

#38. #1 OR #2 OR #3 OR #4 OR #5 OR #6 OR #7 OR #8 OR #9 OR #10 OR #11 OR #12 OR #13 OR #14 OR #15 OR #16 OR #17 OR #18 OR #19 OR #20 OR #21 OR #22

#37. 'animal'/exp NOT ('human'/exp AND 'animal'/exp)

#36. 'single blind procedure'/de

#35. 'crossover procedure'/de

#34. allocate*:ab,ti

#33. assign*:ab,ti

#32. (singl* NEXT/1 blind*):ab,ti

#31. (double* NEXT/1 blind*):ab,ti

#30. crossover*:ab,ti

#29. factorial*:ti,ab

#28. 'placebo'/de

#27. placebo*:ti,ab

#26. 'double blind procedure'/de

#25. random*:ti,ab

#24. 'controlled clinical trial'/de

#23. 'randomized controlled trial'/de

#22. 'fitbit'/exp

#21. 'fitbit':ti,ab

#20. 'wearable activity':ti,ab

#19. 'wearable device*':ti,ab

#18. 'wearable sensor*':ti,ab

#17. 'wearable technolog*':ti,ab

#16. 'wearable track*':ti,ab

#15. 'wearable':ti,ab

#14. 'wearable electronic device*':ti,ab

#13. 'sleep-track*':ti,ab

#12. 'self-track*':ti,ab

#11. 'wrist-worn devices':ti,ab

#10. 'wrist-worn device':ti,ab

#9. 'activity sensors':ti,ab

#8. 'activity sensor':ti,ab

#7. 'activity self-monitor*':ti,ab

#6. 'activity monitor':ti,ab

#5. 'activity track*':ti,ab

#4. 'fitness track*':ti,ab

#3. 'activity tracker'/exp

#2. 'wearable technology'/exp

#1. 'wearable device'/exp

**PubMed:**

1. fitness trackers"[MeSH Terms]
2. fitness track*[Title/Abstract]
3. activity track*[Title/Abstract]
4. activity monitor*[Title/Abstract]
5. activity self-monitor*[Title/Abstract]
6. activity sensor[Title/Abstract]
7. activity sensors[Title/Abstract]
8. wrist-worn device[Title/Abstract]
9. wrist-worn devices[Title/Abstract]
10. Sleep track*[Title/Abstract] OR sleep monitor*[Title/Abstract]
11. wearable electronic devices[MeSH Terms]
12. wearable electronic device*[Title/Abstract]
13. wearables[Title/Abstract]
14. wearable track*[Title/Abstract]
15. wearable technolog*[Title/Abstract]
16. wearable sensor*[Title/Abstract]
17. wearable device*[Title/Abstract]
18. wearable activity[Title/Abstract]
19. Fitbit[Title/Abstract]
20. #1-19/OR
21. randomized controlled trial[Publication Type]
22. controlled clinical trial[Publication Type]
23. randomized[Title/Abstract]
24. placebo[Title/Abstract]
25. clinical trials as topic[MeSH Terms:noexp]
26. randomly[Title/Abstract]
27. trial[Title]
28. #21-27/OR
29. animals[MeSH Terms] NOT (humans[MeSH Terms] AND animals[MeSH Terms]
30. #35 NOT 36
31. #27 AND 37 (Filters: Publication date from 2007/01/01)
