# Supplementary material for: Fitbit-Based Interventions for Healthy Lifestyle Outcomes: Systematic Review and Meta-Analysis
Source: J Med Internet Res. 2020 Oct 12;22(10):e23954. doi: 10.2196/23954 (PMC7589007; doi:10.2196/23954)
Supplement: Multimedia Appendix 2 [file jmir_v22i10e23954_app2.docx]

Qualitative comparative analysis (QCA) is a case-based method that adopts a “system” or “configurational” approach to analysis rather than “variance” or “correlational” approach. Here a case is an intervention that has been assessed as part of a study. In other words, a case corresponds to a set of different factors (e.g., intervention components) that are related to a particular outcome in a study. A study can be represented by several cases, depending on how many outcomes they report.

In QCA, the focus of investigation is not the individual study or a particular factor, but rather the different configurations of intervention components or contextual conditions that, taken together, are responsible for interventions leading to (or not leading to) healthy lifestyle outcomes. In contrast with meta-analysis, QCA is focused on whether the presence or absence of conditions are important to trigger a given outcome. There are two main types of QCA, depending on how the factors are coded. A crisp-set QCA analysis sees factors coded as 1 for present and 0 for absent in each case [1]. In fuzzy-set QCA (used in our review), there is greater flexibility in the categorization, in the sense that a value of 1 indicates full membership of a condition or set, and 0 indicates full non-membership. Values between 0 and 1 then refer to partial membership or partial non-membership (for values between 0.5 and 1 and between 0 and 0.5, respectively) [2]. Assigning such values to conditions within cases is called “calibration” in fsQCA, and it is the first step in its application.

In QCA there are three steps to empirically arrive at this identification of causal conditions. Once the relevant factors in our analyses have been transformed into sets, as described below, the first step involves using these set measures to construct a truth table, which lists all the possible combinations that can influence the outcome of interest. Each row of this table is associated with a specific combination of conditions.

In the second step, the researcher then reduces the number of truth table rows based on two conditions: (i) the minimum number of cases required for a solution to be considered, and (ii) the minimum consistency level of a solution. This step is carried out in order to eliminate marginal configurations and hence focus on those with the highest coverage and consistency. Coverage refers to the proportion of an outcome that is explained by a specific configuration or combination of configurations [3]. Consistency here refers to “the degree to which cases correspond to the set-theoretic relationships expressed in a solution” [4]. In order to estimate consistency when using fuzzy sets, one needs to calculate the proportion of cases that are consistent with the outcome of interest. As suggested by Rihoux and Ragin [5], in our analysis the cut-off was set at 0.8 for raw consistency and at 0.6 for proportional reduction in consistency (PRI consistency). Overall, there were 102 cases (unique combinations of values of conditions and outcomes) for healthy lifestyle outcomes (PA- and weight-related outcomes), comprising 70 cases for PA-outcomes and 32 cases for weight-related outcomes.

In the third step, a Boolean-based algorithm was used to logically reduce the truth table rows to simplified combinations. This algorithm is based on a counterfactual analysis of causal conditions, which allows a categorization of causal conditions into core and peripheral causes. Counterfactual analysis allows us to identify which parts of the observed improvement in outcome is attributable to the impact of the intervention. In our case, this analysis is relevant because few aspects of a combination quickly lead to a very large number of truth table rows.

Because limited diversity is a problem in counterfactual analysis [6], the truth counterfactuals refer to “situations in which a redundant causal condition is added to a set of causal conditions that by themselves already lead to the outcome in question” [4]. In contrast, difficult counterfactuals refer to “situations in which a condition is removed from a set of causal conditions leading to the outcome on the assumption that this condition is redundant” [4]. This distinction between easy and difficult counterfactuals enables us to establish two main types of solutions. The first is a parsimonious solution that includes all simplifying assumptions. The second is an intermediate solution that only includes simplifying assumptions based on easy counterfactuals [4]. It is worth noting that a third solution also exists. This is the most complex one that includes neither easy nor difficult counterfactuals. Such a solution provides minimal insight into causal configurations, due to its lack of simplification and hence aggregation of configurations [4].

As mentioned above, the process of transforming variables into sets requires specifying full membership in a set, full non-membership, and a cross-over point regarding membership. This corresponds to the allocation of the set-memberships of each case in causal condition (i.e., intervention components and study characteristics) and the outcome (i.e., PA- and weight-related outcomes) by transforming the value of each variable for a case into a membership score in a set. First, for each condition we set 0 as the value for components that are not present in the intervention and 1 as the value for components that are present. To assess the effect of intervention’s duration, we considered the exact duration of the intervention, meaning that we calculated its value by taking one year as a reference. For example, a 3-month intervention was given a value of 0.25. It is worth mentioning that the 0.5 value is the point of maximum indifference, so it does not fall into either category (short vs. long follow-up duration). This value of 0.5, which corresponds to a 6-month intervention in our case, was manually set at 0.5001. We conducted a sensitivity analysis by giving the 6-month intervention a value of 0.4999. There was no significant change. We called this factor “follow-up duration.”

Then, for each case, we based membership on the Cohen d values that we calculated for each case. We set as the cut-off values for full-out membership, the crossover point, and full-in membership: a negative Cohen d (i.e., non-beneficial interventions on a particular outcome), 0.2 (small effect of the intervention on a particular outcome), and 0.8 (large effect of the intervention on a particular outcome) [3]. We used these values since a non-beneficial intervention is clearly not in the set of healthy lifestyle outcomes, a large effect is clearly in the set of healthy lifestyle outcomes, and the crossover between negligible and small-effect size was judged to be the point of maximum indifference between whether a condition did or did not have an impact on the outcome. To be coherent with the meaning of negative Cohen d values, we took the reverse value of the Cohen d where this value corresponded to a positive outcome for patients. For instance, if the Cohen d was found to be negative for weight, then this means that the subjects lost weight. This corresponds to a positive outcome for the subjects, so we took the reverse value of this outcome. All of this enables us to conduct analyses using 6 conditions for healthy lifestyle outcomes, because with n conditions, $2^{n}$ should be less than the number of cases. All the analyses were performed using fsQCA software [7].

References

1. Thomas, J., A. O’Mara-Eves, and G. Brunton, *Using qualitative comparative analysis (QCA) in systematic reviews of complex interventions: a worked example.* Systematic Reviews, 2014. **3**(1): p. 67. [doi: 10.1186/2046-4053-3-67], [PMID: 24950727]

2. Lee, S.S., *Using fuzzy-set qualitative comparative analysis.* Epidemiol Health, 2014. **36**: p. e2014038. [doi: 10.4178/epih/e2014038], [PMID: 25666168]

3. Ragin, C.C., *Fuzzy-set social science*. 2000: University of Chicago Press. [ISBN: 0226702774]

4. Fiss, P.C., *Building Better Causal Theories: A Fuzzy Set Approach to Typologies in Organization Research.* Academy of Management Journal, 2011. **54**(2): p. 393-420. [doi: 10.5465/amj.2011.60263120]

5. Rihoux, B. and C.C. Ragin, *Configurational Comparative Methods: Qualitative comparative analysis (QCA) and related techniques*. Vol. 51. 2008, London: Sage. [doi: 10.4135/9781452226569], [ISBN: 9781412942355]

6. Ragin, C.C. and J. Sonnett, *Between Complexity and Parsimony: Limited Diversity, Counterfactual Cases, and Comparative Analysis*, in *Vergleichen in der Politikwissenschaft*, S. Kropp and M. Minkenberg, Editors. 2005, VS Verlag für Sozialwissenschaften: Wiesbaden. p. 180-197. [doi: 10.1007/978-3-322-80441-9_9]

7. Ragin, C.C. 2020. URL: <http://www.socsci.uci.edu/~cragin/fsQCA/software.shtml>
