# Supplementary material for: Fitbit-Based Interventions for Healthy Lifestyle Outcomes: Systematic Review and Meta-Analysis
Source: J Med Internet Res. 2020 Oct 12;22(10):e23954. doi: 10.2196/23954 (PMC7589007; doi:10.2196/23954)
Supplement: Multimedia Appendix 3 [file jmir_v22i10e23954_app3.docx]

| **Author, Year** | **Study Design, Follow-up, Country** | **Population** | **Intervention** | **Comparison** | **Healthy lifestyle-related outcomes** |
| --- | --- | --- | --- | --- | --- |
| Amorim et al. (2019) [1] | Parallel RCT  6 months  Australia | 68 adults who reported chronic LBP persisting for over 12 weeks and discharged from physiotherapy but still symptomatic  (Age: 58.4 ± 13.4; Female: 50%; BMI: 28.0 ± 5.5 kg/m^2^)  Intervention group:  N = 34  LTFU = 3  Age: 59.5 ± 11.9  Female: 44%  BMI: 28.9 ± 6.0 kg/m^2^  Control group:  N = 34  LTFU = 10  Age: 57.1 ± 14.9  Female: 56%  BMI: 27.2 ± 5.1 kg/m^2^ | Information booklet on physical activity and sedentary behavior.  Tailored PA plan.  Face-to-face coaching session.  Twelve phone calls from a health coach.  Personalized messages to encourage participants to achieve their goals (weekly).  Fitbit with its web-interface.  IMPACT app. | Information booklet on physical activity and sedentary behavior. | Secondary outcomes (at 6 months):  Walking (min/week)  Moderate PA (min/week)  Vigorous PA (min/week)  Light PA (min/week)  MVPA (min/week)  Steps (per week)  PA goal attainment (%) |
| Ashe et al (2015) [2] | Parallel RCT  6 months  Canada | 25 healthy, inactive, community-dwelling women, aged 55-70 (Age: 64.1 ± 4.6)  Intervention group:  N = 13  LTFU = 1  Age: 64.8 ±4.6  BMI: 26.9 ±6.8 kg/m^2^  Control group:  N = 12  LTFU = 4  Age: 63.1 ±4.8  BMI: 32.9 ±6.8 kg/m^2^ | Group-based education.  Social support.  Individualized physical activity prescription (called Activity 4-1-1).  Fitbit One. | Education sessions (monthly).  Financial incentives. | Secondary outcomes (at 3 and 6 months):  Steps (per day)  MVPA (min/day)  Sedentary Behavior (min/day, %/day)  Self-rated health  Weight (kg) |
| Azar et al. (2016) [3] | Parallel RCT  6 months  USA | 74 adults with either BMI ≥35 kg/m2 and prediabetes, previous gestational diabetes and/or metabolic syndrome, or BMI ≥30 kg/m2 and type 2 diabetes and/or cardiovascular disease (Age: 59.7 ± 11.2; Female: 59.5%; BMI: 37.1 ± 5.4 kg/m^2^; Race: 5.4% Hispanic, 82.4% Non-Hispanic white, 4.1 Non-Hispanic black, 2.7 Asian/Pacific Islander, 5.4% Other)  Immediate group:  N = 37  LTFU = 5  Age: 59.6 ± 11.9  Female: 59.5%  BMI: 37.0 ± 5.7 kg/m^2^  Race: 5.4% Hispanic, 83.8% Non-Hispanic white, 0 Non-Hispanic black, 2.7 Asian/Pacific Islander, 8.1% Other  Delayed group:  N = 37  LTFU = 5  Age: 59.8 ± 10.5  Female: 59.5%  BMI: 37.3 ± 5.2 kg/m^2^  Race: 5.4% Hispanic, 81.1% Non-Hispanic white, 8.1% Non-Hispanic black, 2.7 Asian/Pacific Islander, 2.7% Other | Technology-training workshop.  Stress management and behavioral lifestyle management group sessions (biweekly).  Online platform and participant portal.  Fitbit.  Withings Smart Scale WS-30  Coach-led virtual small group sessions (weekly).  Coach-led in-person exercise sessions (seven). | Waiting list (same intervention with 3-month delay). | Primary outcomes (at 3, 6 months):  health-related quality of life (mental and physical components)  Secondary outcomes (at 6 months):  BMI (kg/m^2^)  Weight (kg)  Waist circumference (cm)  Diet quality  Physical activity (metabolic  equivalents)  Stress and Mental health |
| Ball et al. (2016) [4] | Parallel RCT  3 months  USA | 89 women with more than one CVD risk factor  (Age: 55.5 ± 9.3; BMI: 34.3 ± 5.2 kg/m^2^)  Intervention group:  N = 30  LTFU = 0  Control group:  N = 28  LTFU = 0 | LEARN program delivered via a podcast with an online interactive message board.  Fitbit. | LEARN program delivered through weekly meetings. | Primary outcomes (at 3 months):  Weight (kg) |
| Brown et al. (2018) [5] | Parallel RCT  15 weeks  USA | 60 patients with prior diagnosis of malignancy, body mass index (BMI) > 25.0 kg/m2, completion of all surgery, chemotherapy, and/or radiation at least 1 month prior to study enrollment and ECOG performance status of 0 or 1 (Age: 52 ± 9; BMI: 31.8 ± 5.4 kg/m^2^)  Intervention group:  N = 30  LTFU = 7  Age: 52 ± 9  Female: 97%  BMI: 32.1 ± 5.6 kg/m^2^  Race: 87% White, 7 % Black, 7% Other  Control group:  N = 30  LTFU = 5  Age: 52 ± 10  Female: 97%  BMI: 31.5 ± 5.3 kg/m^2^  Race: 87% White, 3 % Black, 10 % Other | In-person, group-based program led by a health coach.  Caloric restriction.  Physical activity prescription.  50-min group meetings on healthy eating, portion control, and physical activity (weekly).  Fitbit Flex.  Journal to record food and recreational PA. | Nothing. | Primary outcomes (at 15 weeks):  Body mass (kg)  Secondary outcomes (at 15 weeks):  Fat mass (kg)  Lean Mass (kg)  6-min walk test (m)  MVPA (min/week)  Physical activity (min/week)  Caloric intake (kcal/day)  Fat % (kcal/day)  Carbohydrate (% kcal/day)  Fruits (servings/day)  Vegetables (servings/day)  Fiber (g/day) |
| Cadmus-Bertram et al. (2019) [6] | Parallel RCT  12 weeks  USA | 50 cancer  survivor/support partner dyads with a 28-75 years old cancer survivor diagnosed with Stage I–III colorectal cancer or female breast cancer within the past 5 years, and finished with primary treatment (Age: 54.4 ± 11.2; Female: 96%; BMI: 32.2 ± 7.4 kg/m^2^; Race: 94% Non-Hispanic White, 2% Hispanic, 2% Black, 2% more than one race)  Intervention group:  N = 26 dyads  LTFU = 2 survivors and 3 partners  Age: 52.5 ± 12.2  Female: 100%  BMI: 32.4 ± 6.2 kg/m^2^  Race: 96.2% Non-Hispanic White, 3.8% Hispanic  Control group:  N = 24 dyads  LTFU = 1 survivor and 4 partners  Age: 56.5 ± 9.8  Female: 91.7%  BMI: 33.4 ± 6.5 kg/m^2^  Race: 91.7% Non-Hispanic White, 4.2% Black, 4.2% More than one race | Fitbit Charge HR (some later randomized received a Fitbit Charger 2).  Educational handbook (benefits of physical activity for cancer survivorship, content related to building self-efficacy and setting goals, and information on how to use the Fitbit).  In-person instructional and goal-setting session.  Partner support.  Email-based coaching (at 1, 2, 4, 8 weeks).  EHR integration of Fitbit data. | Educational materials (nutritional recommendations with a brief section relating to physical activity).  Email contact (at 1, 2, 4, 8 weeks). | Primary outcomes (at 12 weeks):  LPA (min/week)  Moderate PA (min/week)  Vigorous PA (min/week)  MVPA (min/week)  MVPA in bouts (min/week)  Steps (per day) |
| Cheung et al. (2019) [7] | Parallel RCT  6 months Australia | 60 adult women suffering from gestational diabetes  Intervention group:  N = 40  LTFU = 21  Age: 34 ± 4  BMI: 28.7 ± 7.6 kg/m^2^  Control group:  N = 20  LTFU = 12  Age: 34 ± 4  BMI: 27.8 ± 4.2 kg/m^2^ | Two 30 min diet counselling sessions.  Fitbit Flex with its mobile app.  Text messages on physical activity, nutrition, and general health and motherhood information and education (3 messages a week from week 3 post-partum).  Messages with adaptative step targets. | Booklet with healthy lifestyle advice. | Primary outcomes (at 6 months):  Diet fat intake <30% of total energy  Sat Fat <10% of total energy  Dietary fiber >15 g/1000 cal  Achieved 150 min/week moderate intensity activity  Achieved mean of 10,000 steps a day  Weight (kg)  Secondary outcomes (at 6 months):  Total daily energy intake (kj)  Change in total daily energy (kj)  Daily fat intake (% total energy)  Daily saturated fat intake (% total energy)  Daily carbohydrate intake (% total energy)  Fiber intake (g/day)  Total weekly activity time (min)  Step count (7 days) |
| Christiansen et al. (2018) [8] | Parallel RCT  6 months  USA | 43 patients undergoing outpatient physical therapy after a unilateral total knee replacement (age = 67.0 ± 7.0, BMI = 31.5 ± 5.9 kg/m^2^, Female: 53.4%)  Intervention group:  N = ?  LTFU = ?  Control group:  N = ?  LTFU = ? | Physical therapy.  Fitbit Zip.  Step goals (individualized weekly)  Phone calls after discharge (monthly). | Physical therapy. | Primary outcomes (when discharge from physical therapy and at 6, 12 months):  Steps (per day)  MVPA (min/week) |
| Christiansen et al. (2018) [9] | Parallel RCT  6 months  USA | Patients seeking outpatient physical therapy after a unilateral total knee replacement, aged 45+  Intervention group:  N = ?  LTFU = ?  Age: 67 ± 7  Female: 37%  BMI: 29.5 ± 2.7 kg/m^2^  Control group:  N = ?  LTFU = ?  Age: 67 ± 7  Female: 64%  BMI: 30.8 ± 5.8 kg/m^2^ | Physical therapy.  Fitbit Zip.  Step goals (individualized weekly)  Phone calls after discharge (monthly). | Physical therapy. | Primary outcomes (when discharge from physical therapy and at 6):  Steps (per day)  MVPA (min/week) |
| Christiansen et al. (2020) [10] | Parallel RCT  6 months and 12 months follow-up  USA | 43 people with a unilateral total knee replacement in outpatient physical therapy (Age: 67 ± 7; Female: 53.4%; BMI: 31.5 ± 5.9 kg/m^2^; White: 91%)  Intervention group:  N = 20  LTFU = 6  Age: 66.9 ± 6.9  Female: 40%  BMI: 31.1 ± 5.6 kg/m^2^  White: 95%  Control group:  N = 23  LTFU = 8  Age: 67.5 ± 7.2  Female: 87%  BMI: 32.0 ± 6.3 kg/m^2^ | Physical therapy.  Fitbit Zip.  Step goals (individualized weekly).  Phone calls to promote PA (monthly). | Physical therapy. | Primary outcomes (when discharge from physical therapy and at 6):  Steps (per day)  MVPA (min/week) |
| DiFrancisco-Donoghue et al. (2018) [11] | Parallel RCT  10 months  USA | 120 first year medical students, aged between 17 and 50 (age: 22.9 ± 2.9)  Fitbit-Plus group:  N = 40  LTFU = 5  Female: 57%  Fitbit-Only group:  N = 40  LTFU = 2  Female: 50%  Control group:  N = 40  LTFU = 0  Female: 52.5% | Fitbit-Plus group:  Fitbit Flex with its website.  Emails offering fitness challenges (weekly).  Goal-setting.  Fitbit-Only group:  Fitbit Flex. | They were asked not to purchase an activity tracker of any kind throughout the study. | Primary outcomes (at 9 months):  Steps (per day) (for the two intervention groups only)  Secondary outcomes (at 9 months):  Weight (kg)  Body fat (%)  Body mass index (kg/m^2^)  Lean body mass (kg)  Fat mass (kg)  Days exercised |
| Duscha et al. (2018) [12] | Parallel RCT  12 weeks  USA | 32 adult patients who completing 36 on-site cardiac rehabilitation sessions  Intervention group:  N = 21  LTFU = 5  Age: 59.9 ± 8.1  Female: 33.3%  BMI: 32.6 ± 8.4 kg/m^2^  Race: 68.8% Non-Hispanic white, 31.2% Black  Control group:  N = 11  LTFU = 2  Age: 66.5 ± 7.2  Female: 87%  BMI: 30.7 ± 6.8 kg/m^2^  Race: 77.8% Non-Hispanic white, 22.2% Black | VIDA health (heath coach selection app).  Personalized coaching.  Fitbit Charge and its mobile app.  Exercise prescription.  Text messages to remind them to practice healthy lifestyle habits. | Standard care. | Primary outcomes (at 9 months):  Peak VO2 (ml/kg/min and L/min)  Steps (per day)  Low Activity (min/day)  Low Activity (min/week)  Moderate-low Activity (min/day)  Moderate-low Activity (min/week)  Moderate-High Activity (min/day)  Moderate-High Activity (min/week)  Total Active (min/day)  Total Active (min/week)  Distance (miles/day)  Distance (miles/week)  Floors (per day)  Floors (per week) |
| Eisenberg et al. (2017) [13] | Factorial RCT  1 week  USA | 157 undergraduates  (Age: 19.62 ± 1.59; Female: 74%; BMI: 23.72 ± 4.32 kg/m^2^; Race: 64.4% White/Caucasian, 4.8% Black/African American, 2.6% Asia, 8.2% Other)  Accelerometer + e-diary group:  N = 40  LTFU = 4  Age: 19.58 ± 1.75  Female: 72.2%  BMI: 23.53 ± 4.94 kg/m^2^  Race: 72.2% White/Caucasian, 13.9% Asia, 13.9% Other  Accelerometer-only group:  N = 39  LTFU = 4  Age: 19.34 ± 1.08  Female: 62.9%  BMI: 23.26 ± 4.11 kg/m^2^  Race: 68.6% White/Caucasian, 11.4% Black/African American, 14.3% Asia, 5.7% Other  E-diary-only group:  N = 37  LTFU = 3  Age: 19.97 ± 1.80  Female: 76.5%  BMI: 22.50 ± 3.52 kg/m^2^  Race: 55.9% White/Caucasian, 8.8% Black/African American, 32.4% Asia, 2.9% Other  Control group:  N = 41  LTFU = 0  Age: 19.59 ± 1.66  Female: 82.9%  BMI: 23.52 ± 3.47 kg/m^2^  Race: 61.0% White/Caucasian, 26.8% Asia, 12.2% Other | Accelerometer + e-diary group:  Fitbit Zip.  Electronic daily dairy on Google Docs word.  Accelerometer-only group:  Fitbit Zip.  E-diary-only group:  Electronic daily dairy on Google Docs word. | Nothing. | Primary outcomes (at 1 week): months):  Steps (per day)  Total PA (METS)  Discrete exercise sessions  Mild PA (min/week)  Moderate PA (min/week)  Vigorous PA (min/week) |
| Falck et al. (2018) [14] | Parallel RCT  6 months  Canada | 61 community dwelling adults with a confirmed diagnosis of knee OA or were 50+ with knee pain lasting more than 28 days in the last year  Immediate group:  N = 30  LTFU = 4  Age: 61.73 ± 9.4  Female: 73.33%  BMI: 29.16 ± 5.46 kg/m^2^  Delayed group:  N = 31  LTFU = 6  Age: 62.61 ± 8.54  Female: 90.32%  BMI: 29.24 ± 4.82 kg/m^2^ | Group session about the benefit of increasing MVPA and reducing sedentary behaviors.  Fitbit Flex.  Individual activity counselling with a physiotherapist (4 biweekly phone calls). | Waiting list (same intervention with 2-month delay). | Secondary outcomes (at 2, 4, 6 months):  MVPA (min/day)  Sedentary behavior (min/day) |
| Farnell et al. (2017) [15] | Parallel RCT  6 weeks  USA | 19 adults who are staff and faculty members (Female: 100%)  Intervention group:  N = 9  LTFU = 0  Control group:  N = 10  LTFU = 0 | Fitbit One. | Nothing. | Primary outcomes (at 6 weeks):  Physical activity score |
| Finkelstein et al. (2016) [16] | Parallel RCT  12 months (6-month trial with a 6-month post-intervention follow-up)  Singapore | 800 employees from 13 companies, 15 worksites (Age: 35; Female 54%; Chinese 69%, Indian 13%, Malay 5%).  Fitbit group:  N=203  LTFU = 17 (intervention phase)  LTFU= 26 (post-intervention phase)  Charity group:  N = 199  LTFU = 21 (intervention phase)  LTFU = 31 (post-intervention phase)  Cash group:  N = 197  LTFU = 7 (intervention phase)  LTFU = 22 (post-intervention phase)  Control group:  N = 201  LTFU = 12 (intervention phase)  LTFU = 24 (post-intervention phase) | All groups  Fitbit Zip and access to its website.  Educational booklets on the benefits and strategies for increasing PA.  Additional intervention components per group are as follows:  Fitbit group:  No additional intervention components.  Charity group:  Charity incentives ($15 if logged between 50,000-70,000 steps/week or $30 if they logged >70,000 steps/week).  Cash group:  Cash incentives ($15 if logged between 50,000-70,000 steps/week or $30 if they logged >70,000 steps/week). | Educational booklets on the benefits and strategies for increasing PA. | Primary outcomes (at 6 and 12 months):  MVPA bout (min/week)  Secondary outcomes (6 and 12 months):  Steps (per day)  Steps (per week)  LPA (min/week)  MVPA (min/week)  Vigorous PA (min/week)  No. of participants meeting PA guideline of >150 minutes of moderate PA per week  No. of participants meeting physical activity guideline of ≥70,000 steps/week  Weight (kg)  Non-exercise testing for cardiorespiratory fitnessVO_2max_ (mL/kg/min)  EQ-5D-5L (quality-of-life score) index |
| Gilmore et al. (2017) [17] | Parallel RCT  16 weeks  USA | 40 adult postpartum women with a BMI between 25 and 40 kg/m^2^ who gave birth less than 8 weeks ago (Age: 26 ± 5.4; BMI: 32 ± 3 kg/m^2^)  E-Moms group:  N = 20  LTFU = 1  Age: 26.0 ± 5.2  BMI: 31.3 ± 3.2 kg/m^2^  Race: 6 White, 12 Black, 1 Asian  WIC Moms group:  N = 20  LTFU = 4  Age: 27.2 ± 6.1  BMI: 32.7 ± 2.8 kg/m^2^  Race: 2 White, 14 Black | Smartloss app and feedbacks on their goal achievement.  BodyTrace scale.  Fitbit Zip.  Step count goal prescription.  Information on diet, physical activity, and behavior modification (weekly).  Personalized treatment advice from the interventionist (delivered remotely through phone conversations, email, and text messages). | Usual care. | Primary outcomes (at 8, 16 weeks):  Weight (kg)  Secondary outcomes (at 8, 16 weeks):  Body fat (%)  Waist circumference (cm)  Hip circumference (cm) |
| Griauzde et al. (2019) [18] | Parallel RCT  12 weeks  USA | 69 prediabetes with a HbA1c level between 5.7% and 6.4%, who are not enrolled in a Diabetes Prevention Programs (Age: 51.7 ± 11.2; Female: 64%)  App-plus group:  N = 22  LTFU = 0  Age: 51.6 ± 11.1  Female: 63.6%  BMI: 33.4 ± 7.8 kg/m^2^  Minority race (other than white): 31.8%  App-only group:  N = 24  LTFU = 7  Age: 52.1 ± 12.0  Female: 62.5%  BMI: 30.7 ± 9.3 kg/m^2^  Minority race: 45.8%  Control group:  N = 23  LTFU = 7  Age: 51.3 ± 11.0  Female: 65.2%  BMI: 33.0 ± 10.4 kg/m^2^  Minority race: 28.6% | App-only group:  Information about prediabetes and evidence-based ways to decrease the progression to T2DM.  List of free mHealth resources for monitoring weight and physical activity.  Mobile app.  App-plus group:  Information about prediabetes and evidence-based ways to decrease the progression to T2DM.  List of free mHealth resources for monitoring weight and physical activity.  Mobile app.  Fitbit scale.  Fitbit.  Tailored messages and health tips. | Information about prediabetes and evidence-based ways to decrease the progression to T2DM.  List of free mHealth resources for monitoring weight and physical activity. | Secondary outcomes (at 12 weeks):  Motivation to prevent type 2 diabetes mellitus |
| Hartman et al. (2016) [19] | Parallel RCT  6 months  USA | 55 overweight (BMI > 27.5) women with  elevated breast cancer risk, aged 40-75 years (Age: 59.5 ± 5.6; BMI: 31.9 ± 3.5)  Intervention group:  N = 36  LTFU = 3  Age: 59.4 ± 5.6  BMI: 32.2 ± 3.4 kg/m^2^  Race: 91.7% White, 19.44% Non-Hispanic  Control group:  N = 18  LTFU = 1  Age: 59.8 ± 5.9  BMI: 31.3 ± 3.7 kg/m^2^  Race: 100% White, 0% Non-Hispanic | PA goal of ≥ 150 min/week of  MVPA.  Calories restriction.  MyFitnessPal app.  Fitbit One.  Twelve standardized coaching calls. | Usual care (dietary guidelines).  Two brief calls. | Primary outcomes (at 6 months):  Weight (kg)  Weight loss (%)  MVPA (min/day)  MVPA (10-min bouts/day) |
| Hartman et al. (2018) [20] | Parallel RCT  12 weeks  USA | 87 sedentary, female breast cancer survivors, aged 21-85 years (Age: 57 ± 10.4; Race: 82% White, 83% non-Hispanic)  Exercise group:  N = 43  LTFU = 1  Age: 58.2 ± 11.37  BMI: 26.7 ± 6.2 kg/m^2^  Race: 83.7% White, 16.3% Non-white  Control group:  N = 44  LTFU = 1  Age: 56.2 ± 9.3  BMI: 27.3 ± 6.4 kg/m^2^  Race: 79.5% White, 22.8% Non-white | 30-minute to 45-minute in-person meeting.  PA goals prescription.  Fitbit One.  2 phone calls from a psychologist.  Emails on generic health topics (every 3 days). | Emails on generic health topics (every 3 days). | Secondary outcomes (at 12 weeks):  MVPA (min/day)  Total activity (min/day)  Meeting 150 min/week (%)  BMI (kg/m^2^) |
| Hornikx et al. (2015) [21] | Parallel RCT  1 month  Belgium | 30 patients with COPD, hospitalized for a COPD exacerbation, aged 40+ years.  Intervention group:  N = 15  LTFU = 3  Age: 66 ± 7  BMI: 25 ± 9 kg/m2  Female: 47%  Control group:  N = 15  LTFU = 0  Age: 68 ± 6  BMI: 29 ± 5 kg/m2  Female: 40% | Fitbit Ultra.  PA counselling (3 times a week).  PA goals. | Usual care (advice about increasing PA). | Primary outcomes (at 2, 4 weeks):  Steps (per day)  Walking time (min/day)  Movement intensity during walking (m/s^2^/day)  Secondary outcomes (at 4 weeks):  Functional exercise capacity (m) |
| Jennings et al. 2016 [22] | Parallel RCT  15 weeks (At week 8, every group received the Fitbit device and the Game Platform).  Singapore | 300 first-year medical students (Age: 18-19; Female: 57.7%)  Intervention group 1:  N = 100  LTFU = 0  Intervention group 2:  N = 100  LTFU = 0  Control group:  N = 100  LTFU = 0 | Intervention group 1:  Educational modules.  Fitbit Flex.  Intervention group 2:  Educational modules.  Fitbit Flex.  Game Platform. | Educational modules. | Primary outcomes (every day, week 1, week 8, week 15):  Steps (per day)  Low PA (min/week)  Moderate PA (min/week)  High PA (min/week)  Sleep time (min/day)  Sleep quality  Stress |
| Katz et al. (2018) [23] | Parallel RCT  21 weeks  USA | 96 physician-diagnosed rheumatoid arthritis patients (Age: 54.8 ± 13.4; Female: 87.5%; BMI: 31.9 ± 8.4 kg/m^2^; Race: 63.5% White, 12.5% African American, 8.5% Asian, 14.6% Other)  Pedometer + step targets group:  N = 34  LTFU = 3  Age: 50.2 ± 14.1  Female: 88.2%  BMI: 33.9 ± 9.9 kg/m^2^  Race: 67.7% White, 11.8% African American, 8.8% Asian, 11.8% Other  Pedometer only group:  N = 34  LTFU = 3  Age: 55.9 ± 12.4  Female: 88.2%  BMI: 32.1 ± 7.2 kg/m^2^  Race: 58.8% White, 17.7% African American, 5.9% Asian, 17.6% Other  Education only group:  N = 28  LTFU = 2  Age: 59.1 ± 12.5  Female: 87.5%  BMI: 29.2 ± 7.3 kg/m^2^  Race: 64.3% White, 7.1% African American, 10.7% Asian, 17.9% Other | Pedometer only group:  Educational booklet.  Discussion on increasing physical activity in daily life.  Fitbit Zip.  Diary (to record step counts).  Pedometer + step targets group:  Educational booklet.  Discussion on increasing physical activity in daily life.  Fitbit Zip.  Diary (to record step counts).  Individualized step goals. | Educational booklet.  Discussion on increasing physical activity in daily life. | Primary outcomes (at 21 weeks):  Steps (per week)  Sedentary activity (< 5000 steps per day, %)  Healthy activity level (≥ 8000 steps per day, %) |
| Kooiman et al. (2018) [24] | Parallel RCT  13 weeks  The Netherlands | 72 adults patients with type 2 diabetes, with HbA1c of 7.5% (58 mmol/mol), engaging in less than 3 hours of intensive exercise per week  (Age: 56 ±11; BMI: 32.9 ±5 kg/m^2^; Female: 47.2%; White: 98.6%)  Intervention group:  N = 40  LTFU = 4  Control group:  N = 32  LTFU = 2 | Usual care.  Fitbit Zip.  Weekly information about physical activity, a healthy diet with sample recipes appropriate for those with diabetes, and videos of strength exercises with an explanation of how to build muscle strength via an online self-tracking program.  To set incremental activity goals.  Tailored feedback messages. | Usual care. | Secondary outcomes:  Steps (per day) (per week and 12 weeks)  Days with more than 30 min of MVPA (12 weeks)  Weight (kg) (12 weeks)  BMI (kg/m^2^) (12 weeks)  Waist circumference (cm) (12 weeks)  Hip circumference (cm) (12 weeks)  Subjective health score (12 weeks)  Intention toward engagement  in exercise (12 weeks)  Attitude toward engagement  in exercise (12 weeks)  Self-efficacy toward engagement  in exercise (12 weeks)  Social norm toward engagement  in exercise (12 weeks) |
| Li et al. (2017) [25] | Parallel RCT  2 months  Canada | 34 patients with knee  osteoarthritis or aged 50+ and experiencing pain in or around knee during 28 days (Age: 55.5 ± 8.6  Female: 82%  BMI: 27.2 ± 4.7 kg/m^2^)  Immediate group:  N = 17  LTFU = 0  Age: 52.3 ± 9.7  Female: 82%  BMI: 29.1 ± 4.5 kg/m^2^  Delayed group:  N = 17  LTFU = 0  Age: 58.7 ± 6.0  Female: 82%  BMI: 25.4 ± 4.2 kg/m^2^ | Group education session about PA.  Telephone coaching by a physical therapist (weekly).  Fitbit Flex and its website. | Waiting list (same intervention with 1-month delay). | Primary outcomes (at 1, 2 months):  MVPA (in bouts per min/day) ≥3 METS  MVPA (in bouts per min/day) ≥4 METS  Secondary outcomes (at 1, 2 months):  Sedentary behavior (bouts in min) |
| Li et al. (2018) [26] | Parallel RCT  6 months  Canada | 61 patients with knee  osteoarthritis or aged 50+ and experiencing pain in or around knee during 28 days (Age: 61.7 ± 8.9; Female: 82%; BMI: 29.2 ± 5.1 kg/m^2^)  Immediate group:  N = 30  LTFU = 1  Age: 61.3 ± 9.4  Female: 73%  BMI: 29.2 ± 5.5 kg/m^2^  Delayed group:  N = 31  LTFU = 4  Age: 62.1 ± 8.5  Female: 90%  BMI: 29.2 ± 4.8 kg/m^2^ | Group education session by a physical therapist.  Fitbit Flex and its website.  Phone calls for activity counselling (4 biweekly). | Waiting list (same intervention with 2-month delay). | Primary outcomes (at 2.4.6 months):  MVPA (≥3 METs)  Secondary outcomes (at 2.4.6 months):  MVPA (≥4 METs)  Sedentary behavior (bouted, min/day)  Steps (per day) |
| Lystrup et al. (2016) [27] | Parallel RCT  12 months  USA | 107 military medical students (Age: 25.4 ±2.1; Female: 37%; BMI: 23.8 ±2.3 kg/m^2^)  Intervention group:  N = 50  LTFU = 6  Age: 24.7 ±2.0  Female: 32%  BMI: 23.4 ±2.4 kg/m^2^  Control group:  N = 57  LTFU = 15  Age: 26.2 ±3.7  Female: 41%  BMI: 24.2 ± 2.5 kg/m^2^ | Brief presentation on PA.  Fitbit Zip. | Brief presentation on PA. | Primary outcomes (12 months):  Physical fitness test (time for 1.5- and 2-mile runs)  Secondary outcomes (per month):  Number of participants achieving a monthly average of >10,000 steps/day  Exercise frequency (at baseline only)  BMI (kg/m^2^) (at baseline only) |
| Mahar et al. (2015) [28] | Parallel RCT  10 weeks  USA | 75 college students  Intervention group:  N = 34  LTFU = ?  Control group:  N = 41  LTFU = ? | Fitbit Flex. | Nothing. | Primary outcomes (at 10 weeks):  Steps (per day)  Physical activity  Self-determined motivation for PA  PA enjoyment |
| McDermott et al. (2018) [29] | Parallel RCT  9 months  USA | 200 patients with an ankle brachial index of 0.90 or less, aged 50+ (Age: 70.2 ± 10.4; Female: 52.5%)  Intervention group:  N = 99  LTFU = 11  Age: 70.1 ± 10.6  Female: 54.5%  BMI: 29.6 ± 5.3 kg/m^2^  Race: 49.5% Black  Control group:  N = 101  LTFU = 7  Age: 70.4 ± 10.1  Female: 50.5%  BMI: 29.9 ± 5.3 kg/m^2^  Race: 50.5% Black | Sessions with the coach or with other participants and the coach (4 weekly).  Phone calls from the coach (weekly for the first two months, biweekly for the next two months, and monthly after 5 months).  Fitbit Zip.  Group phone calls led by the coach (twice per months).  Access to the study website.  Goals setting for walking exercise. | Nothing. | Primary outcomes (at 9 months):  6-minute walk distance (m)  Secondary outcomes (at 9 months):  WIQ score on distance, speed and stair climbing  Physical functioning  Steps (per week)  Distance Walked During Past Week (No. of Blocks)  Walking Exercise Frequency During Past Week (No. of Episodes) |
| Mendoza et al. (2017) [30] | Parallel RCT  10 weeks  USA | 59 14–18 years  old, ≥1-year post cancer therapy adolescents (Age: 16.6 ± 1.5;  Female: 59.3%; Race: 71.2% Non-Hispanic White, 3.4% Non-Hispanic Black, 11.9% Hispanic, 13.6% Other)  Intervention group:  N = 29  LTFU = 0  Age: 16.9 ± 1.5  Female: 58.6%  Race: 65.5% Non-Hispanic White, 6.9% Non-Hispanic Black, 13.8% Hispanic, 13.8% Other  Control group:  N = 30  LTFU = 0  Age: 16.3 ± 1.5  Female: 60%  Race: 76.7% Non-Hispanic White, 0% Non-Hispanic Black, 10% Hispanic, 13.3% Other | Fitbit Flex with associated app.  Facebook group (rewards for PA, forum).  Step goals prescription (weekly).  Text messages for PA (every other day). | Usual care (discussion on the importance of PA). | Secondary outcomes (at 9 months):  MVPA (min/day)  Sedentary Behavior (min/day)  PA enjoyment  Motivation for PA |
| Miragall et al. (2018) [31] | Parallel RCT  3 weeks (intervention), 3 months (follow-up)  Spain | 84 university students practicing less than 30 min of moderate-intensity PA three  times a week, being sedentary or low active, aged between 18 and 40 years old  (Age: 22.18 ±3.71; BMI: 21.71 ±3.16 kg/m^2^; Female: 85.5%).  IMI group:  N = 28  LTFU = 4  IMI+PED group:  N = 28  LTFU = 2  Control group:  N = 28  LTFU = 2 | IMI group:  Information to increase motivation to do PA and set individualized goals.  A blinded pedometer.  IMI+PED group:  Information to increase motivation to do PA and set individualized goals.  Fitbit One with associated website. | Use of a blinded pedometer. | Primary outcomes (at 3 weeks, 3 months):  Steps (per day)  Secondary outcomes (at 3 weeks, 3 months):  PA enjoyment  Stage of PA behavior  Decision-making related to PA  Self-efficacy  Processes of change to practice PA (consciousness raising, dramatic relief, environment reevaluation, self-Reevaluation, Social Liberation, Counterconditioning, Helping Relationships, Reinforcement Management, Self-Liberation, Stimulus Control) |
| Oliveira et al. (2019) [32] | Parallel RCT  6 months (intervention), 6 months (follow-up)  Australia | 131 community-dwelling people who are more than 60 years and still living at home.  Intervention group:  N = 64  LTFU = 4 (at 6 months), 4 (at 12 months)  Age: 71 ± 6  Female: 67%  Control group:  N = 67  LTFU = 1 (at 6 months), 8 (at 12 months)  Age: 72 ± 7  Female: 75% | A face-to-face health coaching session by a physiotherapist.  Mobility-related goals prescription.  Fitbit with associated website.  Tailored advice.  Fall prevention advice brochure.  Telephone health coaching. | Fall prevention advice brochure. | Primary outcomes (at 6, 12 months):  PA participation (counts/min)  Secondary outcomes (at 6, 12 months):  Steps (per day)  Meeting 150 min of MVPA per week (counts)  Quality of life |
| Paxton et al. (2018) [33] | Parallel RCT  12 weeks  USA | 45 50-75 years old individuals who underwent unilateral total knee arthroplasty  Intervention group:  N = 22  LTFU = 0  Age: 63 ± 7  Female: 59.09%  BMI: 29.9 ± 10.7 kg/m^2^  Control group:  N = 23  LTFU = 4  Age: 64 ± 6  Female: 47.83%  BMI: 26.4 ± 8.6 kg/m^2^ | Standard inpatient and home rehabilitation programs.  PA goal prescription (weekly).  Fitbit Zip.  Group meeting on PA guidelines (monthly). | Standard inpatient and home rehabilitation programs.  Phone meetings (weekly). | Secondary outcomes (at 12 weeks):  Timed-up-and-go test (s)  Steps (per day)  6-minute walk test (m)  Four-meter walk test |
| Redman et al. (2017) [34] | Parallel RCT  36 weeks  USA | 54 overweight and obese women aged 18 to 40 years expecting  a singleton pregnancy in their first trimester.  In-person group:  N = 18  LTFU = 0  Age: 29.2 ± 4.8  Race: 28% Black, 61% White, 11% Other  Remote group:  N = 19  LTFU = 0  Age: 29.0 ± 4.2  Race: 11% Black, 84% White, 5% Other  Control group:  N = 17  LTFU = 0  Age: 29.5 ± 5.1  Race: 35% Black, 65% White | In-person group:  Dietary intake and exercise advice.  18 sessions of behavior modification counseling.  Internet-connected bathroom scale.  Pedometer (Omron).  Mobile apps (weight management system).  Individualized calorie intake prescription.  Remote group:  Dietary intake and exercise advice.  18 sessions of behavior modification counseling.  Internet-connected bathroom scale.  Fitbit Zip.  Mobile apps (weight management system).  Individualized calorie intake prescription. | Usual care. | Primary outcomes (over 36 weeks):  Weight (kg) |
| Shoemaker et al. (2016) [35] | Parallel RCT  3 months (intervention), 6 months (follow-up)  USA | 16 patients older than 40 years with diagnosis of heart failure (Age: 66 ± 14; BMI: 29.8 ± 13.6 kg/m^2^)  Exercise/health coaching group:  N = 6  LTFU = 1  Age: 69.5 ± 19.25  BMI: 32.6 ± 11.47 kg/m^2^  Feedback/Encouragement group:  N = 6  LTFU = 1  Age: 62 ± 18.75  BMI: 31.05 ± 22.68 kg/m^2^  Control group:  N = 4  LTFU = 0  Age: 63 ± 22.75  BMI: 29.45 ± 15.72 kg/m^2^ | Exercise/health coaching group:  3 individualized home exercise program.  Health coaching (weekly).  Physical therapy (weekly).  Feedback/Encouragement group:  Fitbit Zip with associated app.  Encouragement and Feedback (weekly). | Usual care. | Primary outcomes (at 3, 9 months):  PA activity (hr/day)  Sedentary activity (%)  Light activity (hr/day)  Moderate Activity (hr/day)  Vigorous Activity (hr/day)  Secondary outcomes (at 3, 9 months):  6-minute walk test (m)  Health status  30-second timed chair rise (repetitions) |
| Simons et al. (2018) [36] | Parallel RCT  9 weeks (intervention), 12 weeks (follow-up)  Belgium | 130 employees between 18 and 30 with lower education and not meeting 150 min of MVPA a week guidelines (Age: 25 ± 3; Female: 51.5%; BMI: 24.5 ± 4.5 kg/m^2^)  Intervention group:  N = 60  LTFU = 5 (at 9 weeks), 2 (at 21 weeks)  Age: 24.8 ± 3.1  Female: 41.7%  BMI: 24.9 ± 4.5 kg/m^2^  Control group:  N = 70  LTFU = 7 (at 9 weeks), 6 (at end of follow-up)  Age: 25.1 ± 3  Female: 60%  BMI: 24.1 ± 4.4 kg/m^2^ | Active Coach app (with personal goals and interventionist feedbacks).  Fitbit Charge.  Messages regarding tips, facts, and their goal. | Generic physical activity information. | Primary outcomes (at 9, 21 weeks):  Light PA (min/day)  Moderate PA (min/day)  Vigorous PA (min/day)  MVPA (min/day)  Total PA (min/day)  Steps (per day)  Occupational PA (min/week)  Active transport (min/week)  Household PA (min/week)  Recreational PA (min/week)  Total PA (min/week)  Social support for PA  Self-efficacy |
| Sloan et al. (2018) [37] | Parallel RCT  12 months (6-month trial with a 6 month post-intervention follow-up)  Singapore | 800 employees from 13 companies, 15 worksites (Age: 35; Female 54%; Chinese 69%, Indian 13%, Malay 5%).  Fitbit group:  N=203  LTFU = 17 (intervention phase)  LTFU= 26 (post-intervention phase)  Charity group:  N = 199  LTFU = 21 (intervention phase)  LTFU = 31 (post-intervention phase)  Cash group:  N = 197  LTFU = 7 (intervention phase)  LTFU = 22 (post-intervention phase)  Control group:  N = 201  LTFU = 12 (intervention phase)  LTFU = 24 (post-intervention phase) | All groups  Fitbit Zip.  Educational booklets on the benefits and strategies for increasing PA.  Weekly participation payment of $4, irrespective of number of steps recorded.  Additional intervention components per group are as follows:  Fitbit group:  No additional intervention components.  Charity group:  Access to the Fitbit website.  Charity incentives ($15 if logged between 50,000-70,000 steps/week or $30 if they logged >70,000 steps/week).  Cash group:  Access to the Fitbit website.  Cash incentives ($15 if logged between 50,000-70,000 steps/week or $30 if they logged >70,000 steps/week). | $4/week for participation in the study | Primary outcomes (at 6, 12 months):  Sedentary time (%/day)  Prolonged sedentary 30 min-bouts (%/day) |
| Thompson et al. 2014 [38] | Crossover RCT  48 weeks (24 weeks of intervention), followed by crossover (24 weeks)  USA | 49 persons aged 65–95, ambulatory but sedentary and overweight.  Intervention group:  N = 25  LTFU = 1  (Age: 79.1 ±8.0; Female: 79%)  Control group:  N = 24  LTFU = 0  (Age: 79.8 ±6.0; Female: 83%) | Fitbit with feedback.  Individual exercise counseling  NIH program Go4Life consisting of 3 phases: “Get READY”: education on exercises promoting endurance, strength, balance, and flexibility; “Get SET”: preparation for behavior change by goal setting and planning, “GO!”: carrying out exercise plans and tracking physical activity.  Phone calls with counsellor (weekly).  Face-to-face counselling (every 2 months). | Fitbit without feedback. | Primary outcomes (6, 12 months):  Physical activity (activity units per day)  Weight (kg)  Waist (cm)  Body fat (%)  Get Up&Go (s) |
| Thorndike et al, 2014 [39] | Parallel RCT  12 weeks (two phases of 6-weeks; phase 1 was a RCT, while phase 2 involved a non-randomized team competition)  USA | 104 medicine residents (Age: 29, range: 23-37; BMI: 24.1 kg/m^2^, range 17.8-35.6; Female 54%, White 66%)  Intervention group:  N = 52  LTFU = 2  Age: 29, range: 23-36  BMI: 23.7 kg/m^2^, range 19.4-34.9  Female: 51%  Race: 66% White  Control group:  N = 52  LTFU = 3  Age: 29, range: 25-37  BMI: 24.6 kg/m^2^, range 17.8-35.6  Female: 56%  Race: 65% White | Fitbit with associated website.  One-hour personal training session (weekly).  Access to a nutrition program.  Special lunches prepared on a weekly basis.  Possibility to schedule two individual appointments with a nutritionist. | Blinded Fitbit with associated website.  One-hour personal training session (weekly).  Access to a nutrition program.  Special lunches prepared on a weekly basis.  Possibility to schedule two individual appointments with a nutritionist. | Primary outcomes:  Steps (median steps/day) (weekly, 3 and 6 months)  Weight (lbs) (at 12 weeks)  Body mass index (kg/m^2^) (at 12 weeks)  Waist (inches) (at 12 weeks) |
| Van Blarigan et al. (2019) [40] | Parallel RCT  12 weeks  USA | 41 individuals with non-metastatic colon or rectal cancer who had completed curative therapy (Age: 54 ± 14; Female: 59%; BMI: 28.4 ± 5.9 kg/m^2^; Race: 73% White, 2% African American/Black, 12% Asian, 12% Other)  Intervention group:  N = 21  LTFU = 0  Age: 56 ± 12  Female: 60%  BMI: 29.7 ± 7.2 kg/m^2^  Race: 70% White, 5% African American/Black, 10% Asian, 15% Other  Control group:  N = 21  LTFU = 2  Age: 54 ± 11  BMI: 27.1 kg/m^2^ ± 4.3 kg/m^2^  Female: 57%  Race: 76% White, 14% Asian, 10% Other | Educational materials about physical activity after cancer.  Fitbit Flex with associated website.  Text messages about prompts for counseling, tips, etc. (daily). | Educational materials about physical activity after cancer. | Secondary outcomes (at 12 weeks):  Moderate-to-vigorous PA (min/day)  Moderate PA (min/day)  Vigorous PA (min/day)  Steps (per day)  Sedentary behavior (min/day) (not reported) |
| Vandelanotte et al. (2018) [41] | Parallel RCT  3 months  Australia | 243 adults with a BMI between 25 and 40 who are engaged in less than 150 min/week of MVPA (Age: 51.5 ± 11.1; Female: 74.9%; BMI: 31.2 kg/m^2^ ± 4.5 kg/m^2^)  Fitbit group:  N = 121  LTFU = 43  Age: 51.6 ± 11.6  Female: 73.6%  BMI: 31.4 ± 4.4 kg/m^2^  Non-Fitbit group:  N = 122  LTFU = 76  Age: 51.5 ± 10.6  Female: 76.2%  BMI: 31.1 kg/m^2^ ± 4.7 kg/m^2^ | TaylorActive website with personalized advice (from the Fitbit device).  Behavior change content across 8 modules of personal physical activity advice.  Access to a Library with generic educational information about physical activity.  Fitbit Flex.  To complete an action plan. | TaylorActive website with personalized advice (from a questionnaire).  Behavior change content across 8 modules of personal physical activity advice.  Access to a Library with generic educational information about physical activity.  To complete an action plan. | Primary outcomes (at 1, 3 months):  Total physical activity (min/week)  MVPA (min/week)  Sitting (min/day)  Body mass index (kg/m^2^) |

References

1. Amorim, A.B., et al., *Integrating Mobile-health, health coaching, and physical activity to reduce the burden of chronic low back pain trial (IMPACT): a pilot randomised controlled trial.* BMC Musculoskelet Disord, 2019. **20**(1): p. 71. [doi: 10.1186/s12891-019-2454-y], [PMID: 30744606]

2. Ashe, M.C., et al., *"Not just another walking program": Everyday Activity Supports You (EASY) model-a randomized pilot study for a parallel randomized controlled trial.* Pilot Feasibility Stud, 2015. **1**: p. 4. [doi: 10.1186/2055-5784-1-4]

3. Azar, K.M.J., et al., *The Electronic CardioMetabolic Program (eCMP) for Patients With Cardiometabolic Risk: A Randomized Controlled Trial.* J Med Internet Res, 2016. **18**(5): p. e134. [doi: 10.2196/jmir.5143], [PMID: 27234480]

4. Ball, C.A., et al., *Impact of Digital Health Methods for Weight Management on Atherosclerotic Cardiovascular Disease Risk in “at-risk” Women.* Canadian Journal of Cardiology, 2016. **32**(4): p. S9-S10. [doi: 10.1016/j.cjca.2016.02.026]

5. Brown, J.C., et al., *Randomized trial of a clinic-based weight loss intervention in cancer survivors.* Journal of Cancer Survivorship, 2018. **12**(2): p. 186-195. [doi: 10.1007/s11764-017-0657-5], [PMID: 29101711]

6. Cadmus-Bertram, L., et al., *Building a physical activity intervention into clinical care for breast and colorectal cancer survivors in Wisconsin: a randomized controlled pilot trial.* J Cancer Surviv, 2019. [doi: 10.1007/s11764-019-00778-6], [PMID: 31264183]

7. Cheung, N.W., et al., *A Pilot Randomised Controlled Trial of a Text Messaging Intervention with Customisation Using Linked Data from Wireless Wearable Activity Monitors to Improve Risk Factors Following Gestational Diabetes.* Nutrients, 2019. **11**(3). [doi: 10.3390/nu11030590], [PMID: 30862052]

8. Christiansen, M., et al., *1-year outcomes from a novel physical therapist-administered physical activity intervention after total knee replacement: A pilot study.* Arthritis and Rheumatology, 2018. **70**: p. 439-440. [doi: 10.1002/art.40700]

9. Christiansen, M.B., et al., *Preliminary findings of a novel physical therapist administered physical activity intervention after total knee replacement.* Osteoarthritis and Cartilage, 2018. **26**: p. S334. [doi: 10.1016/j.joca.2018.02.663]

10. Christiansen, M.B., et al., *The feasibility and preliminary outcomes of a physical therapist-administered physical activity intervention after total knee replacement.* Arthritis Care Res (Hoboken), 2020. [doi: 10.1002/acr.23882], [PMID: 30908867]

11. DiFrancisco-Donoghue, J., et al., *Utilizing wearable technology to increase physical activity in future physicians: A randomized trial.* Preventive Medicine Reports, 2018. **12**: p. 122-127. [doi: 10.1016/j.pmedr.2018.09.004], [PMID: 30234000]

12. Duscha, B.D., et al., *Effects of a 12-week mHealth program on peak VO2 and physical activity patterns after completing cardiac rehabilitation: A randomized controlled trial.* American Heart Journal, 2018. **199**: p. 105-114. [doi: 10.1016/j.ahj.2018.02.001], [PMID: 29754647]

13. Eisenberg, M.H., et al., *The impact of E-diaries and accelerometers on young adults' perceived and objectively assessed physical activity.* Psychology of Sport & Exercise, 2017. **30**: p. 55-63. [doi: 10.1016/j.psychsport.2017.01.008]

14. Falck, R.S., et al., *Can we improve cognitive function among adults with osteoarthritis by increasing moderate-to-vigorous physical activity and reducing sedentary behaviour? Secondary analysis of the MONITOR-OA study.* BMC Musculoskelet Disord, 2018. **19**(1): p. 447. [doi: 10.1186/s12891-018-2369-z], [PMID: 30577819]

15. Farnell, G. and J. Barkley, *The effect of a wearable physical activity monitor (Fitbit One) on physical activity behaviour in women: A pilot study.* Journal of Human Sport and Exercise, 2017. **12**. [doi: 10.14198/jhse.2017.124.09]

16. Finkelstein, E.A., et al., *Effectiveness of activity trackers with and without incentives to increase physical activity (TRIPPA): a randomised controlled trial.* The Lancet Diabetes and Endocrinology, 2016. **4**(12): p. 983-995. [doi: 10.1016/S2213-8587(16)30284-4]

17. Gilmore, L.A., et al., *Personalized Mobile Health Intervention for Health and Weight Loss in Postpartum Women Receiving Women, Infants, and Children Benefit: A Randomized Controlled Pilot Study.* Journal of Women's Health, 2017. **26**(7): p. 719-727. [doi: 10.1089/jwh.2016.5947], [PMID: 28338403]

18. Griauzde, D., et al., *A Mobile Phone-Based Program to Promote Healthy Behaviors Among Adults With Prediabetes Who Declined Participation in Free Diabetes Prevention Programs: Mixed-Methods Pilot Randomized Controlled Trial.* JMIR Mhealth Uhealth, 2019. **7**(1): p. e11267. [doi: 10.2196/11267], [PMID: 30626566]

19. Hartman, S.J., et al., *Technology- and Phone-Based Weight Loss Intervention: Pilot RCT in Women at Elevated Breast Cancer Risk.* Am J Prev Med, 2016. **51**(5): p. 714-721. [doi: 10.1016/j.amepre.2016.06.024], [PMID: 27593420]

20. Hartman, S.J., et al., *Randomized controlled trial of increasing physical activity on objectively measured and self-reported cognitive functioning among breast cancer survivors: The memory & motion study.* Cancer, 2018. **124**(1): p. 192-202. [doi: 10.1002/cncr.30987], [PMID: 28926676]

21. Hornikx, M., et al., *The effects of a physical activity counseling program after an exacerbation in patients with Chronic Obstructive Pulmonary Disease: a randomized controlled pilot study.* BMC Pulm Med, 2015. **15**: p. 136. [doi: 10.1186/s12890-015-0126-8]

22. Jennings, F.H., et al., *Promote Students’ Healthy Behavior Through Sensor and Game: A Randomized Controlled Trial.* Medical Science Educator, 2016. **26**(3): p. 349-355. [doi: 10.1007/s40670-016-0253-8]

23. Katz, P., et al., *Physical Activity to Reduce Fatigue in Rheumatoid Arthritis: A Randomized Controlled Trial.* Arthritis Care Res (Hoboken), 2018. **70**(1): p. 1-10. [doi: 10.1002/acr.23230], [PMID: 28378441]

24. Kooiman, T.J.M., et al., *Self-tracking of Physical Activity in People With Type 2 Diabetes: A Randomized Controlled Trial.* CIN: Computers, Informatics, Nursing, 2018. **36**(7): p. 340-349. [doi: 10.1097/CIN.0000000000000443], [PMID: 29742550]

25. Li, L.C., et al., *A Community-Based Physical Activity Counselling Program for People With Knee Osteoarthritis: Feasibility and Preliminary Efficacy of the Track-OA Study.* JMIR Mhealth Uhealth, 2017. **5**(6): p. e86. [doi: 10.2196/mhealth.7863], [PMID: 28652228]

26. Li, L.C., et al., *Efficacy of a Community-Based Technology-Enabled Physical Activity Counseling Program for People With Knee Osteoarthritis: Proof-of-Concept Study.* J Med Internet Res, 2018. **20**(4): p. e159. [doi: 10.2196/jmir.8514], [PMID: 29712630]

27. Lystrup, R.M., et al., *Pedometry to Prevent Cardiorespiratory Fitness Decline-Is it Effective?* Military medicine, 2016. **181**(10): p. 1235‐1239. [doi: 10.7205/MILMED-D-15-00540], [PMID: 27753558]

28. Mahar, M.T., et al., *Effects of an Intervention using Movement Technology in a University Physical Activity Class: 1902 Board #247 May 28, 330 PM - 500 PM.* Medicine & Science in Sports & Exercise, 2015. **47**(5S): p. 522. [doi: 10.1249/01.mss.0000477869.71815.de]

29. McDermott, M.M., et al., *Effect of a Home-Based Exercise Intervention of Wearable Technology and Telephone Coaching on Walking Performance in Peripheral Artery Disease: The HONOR Randomized Clinical Trial.* Jama, 2018. **319**(16): p. 1665-1676. [doi: 10.1001/jama.2018.3275], [PMID: 29710165]

30. Mendoza, J.A., et al., *A Fitbit and Facebook mHealth intervention for promoting physical activity among adolescent and young adult childhood cancer survivors: A pilot study.* Pediatr Blood Cancer, 2017. **64**(12). [doi: 10.1002/pbc.26660]

31. Miragall, M., et al., *Increasing physical activity through an Internet-based motivational intervention supported by pedometers in a sample of sedentary students: A randomised controlled trial.* Psychology & Health, 2018. **33**(4): p. 465-482. [doi: 10.1080/08870446.2017.1368511], [PMID: 28880576]

32. Oliveira, J.S., et al., *A combined physical activity and fall prevention intervention improved mobility-related goal attainment but not physical activity in older adults: a randomised trial.* Journal of Physiotherapy (Elsevier), 2019. **65**(1): p. 16-22. [doi: 10.1016/j.jphys.2018.11.005], [PMID: 30581138]

33. Paxton, R.J., et al., *A Feasibility Study for Improved Physical Activity After Total Knee Arthroplasty.* J Aging Phys Act, 2018. **26**(1): p. 7-13. [doi: 10.1123/japa.2016-0268], [PMID: 28338406]

34. Redman, L.M., et al., *Effectiveness of SmartMoms, a Novel eHealth Intervention for Management of Gestational Weight Gain: Randomized Controlled Pilot Trial.* JMIR Mhealth Uhealth, 2017. **5**(9): p. e133. [doi: 10.2196/mhealth.8228], [PMID: 28903892]

35. Shoemaker, M.J., et al., *Exercise- and Psychosocial-Based Interventions to Improve Daily Activity in Heart Failure: A Pilot Study.* Home Health Care Management & Practice, 2016. **29**(2): p. 111-120. [doi: 10.1177/1084822316683660]

36. Simons, D., et al., *Effect and Process Evaluation of a Smartphone App to Promote an Active Lifestyle in Lower Educated Working Young Adults: Cluster Randomized Controlled Trial.* JMIR Mhealth Uhealth, 2018. **6**(8): p. e10003. [doi: 10.2196/10003], [PMID: 30143477]

37. Sloan, R.A., et al., *The influence of a consumer-wearable activity tracker on sedentary time and prolonged sedentary bouts: secondary analysis of a randomized controlled trial.* BMC research notes, 2018. **11**(1): p. 189. [doi: 10.1186/s13104-018-3306-9], [PMID: 29566746]

38. Thompson, W.G., et al., *“Go4Life” exercise counseling, accelerometer feedback, and activity levels in older people.* Archives of Gerontology and Geriatrics, 2014. **58**(3): p. 314-319. [doi: 10.1016/j.archger.2014.01.004], [PMID: 24485546]

39. Thorndike, A.N., et al., *Activity monitor intervention to promote physical activity of physicians-in-training: randomized controlled trial.* Plos one, 2014. **9**(6): p. e100251. [doi: 10.1371/journal.pone.0100251], [PMID: 24950218]

40. Van Blarigan, E.L., et al., *Self-monitoring and reminder text messages to increase physical activity in colorectal cancer survivors (Smart Pace): a pilot randomized controlled trial.* BMC Cancer, 2019. **19**(1): p. 218. [doi: 10.1186/s12885-019-5427-5], [PMID: 30866859]

41. Vandelanotte, C., et al., *The Effectiveness of a Web-Based Computer-Tailored Physical Activity Intervention Using Fitbit Activity Trackers: Randomized Trial.* Journal of medical Internet research, 2018. **20**(12): p. e11321. [doi: 10.2196/11321], [PMID: 30563808]
