# Supplementary material for: Fitbit-Based Interventions for Healthy Lifestyle Outcomes: Systematic Review and Meta-Analysis
Source: J Med Internet Res. 2020 Oct 12;22(10):e23954. doi: 10.2196/23954 (PMC7589007; doi:10.2196/23954)
Supplement: Multimedia Appendix 5 [file jmir_v22i10e23954_app5.docx]

Exclusive use of the device’s companion app was reported in 10.81% (4/37) of the included studies [1-4], and the Fitbit website alone was used in 21.62% (8/37) of the studies [5-12]. Only two studies used both the companion app and the associated website in their interventions [13, 14]. The app and the website were used to synchronize with the device and/or to self-monitor one’s behavior throughout the intervention. Some interventions also involved another app (6/37, 16.22%) [3, 5, 15-18] or a website for use by participants (6/37, 16.22%) [8, 19-23]. For instance, Azar, Koliwad [19] hosted program materials on a comprehensive online platform and participant portal paired with a Fitbit device.

The most frequent intervention component in the included studies was related to goal-setting and prescription (22/37, 59.46%). This category pertains to improving particular outcomes (e.g., PA-related) or restricting others (e.g., caloric restriction). While most of these goals pertain to PA [1-5, 11, 13, 15, 18, 21-29], some studies were related to PA improvement and caloric restriction [6, 30], caloric restriction only [17], or mobility goals [31].

Interventional components related to education took the form of information materials (e.g., booklet, online platform) delivered to each individual and focused on lifestyle or existing conditions [5, 8, 11, 13-16, 19, 20, 23, 28, 31-33]. Group sessions were another way to educate participants [6, 9, 10, 24]. Some educational content was offered through a website [21, 34], and one study offered participants the opportunity to schedule an appointment with a nutritionist to acquire knowledge about healthy diets [12]. Overall, most of the educational material was related to general physical activity [5, 6, 8-11, 15, 19, 21, 23, 24, 28, 33, 34], with some content focused on PA after cancer [13, 14] or with osteoarthritis [10]. In addition, the content also included information on diet [6, 12, 15, 21], sedentary behavior [5, 10], the importance of sleep [32], the consequences of chronic stress [32], fall prevention [31], how to improve self-efficacy [13], and guidance on how to slow progression to type 2 diabetes mellitus [16]. One study did not describe the content of its educational intervention component [20]. Taken together, 56.76% (21/37) of the studies included this type of intervention component.

Besides the self-monitoring capabilities and feedback provided by the wearable itself, half of the interventions (20/37, 54.05%) included feedback or counseling delivered by individuals with various academic backgrounds, including trained health or lifestyle coaches [3, 5, 6, 19, 22, 34], and medical professionals, such as dieticians and therapists [1, 2, 9, 10, 26, 31]. Some advice was also provided by the research team [13, 27, 30], and one study used algorithms and a web-based interface to deliver personalized advice related to PA [23]. Two studies did not mention how the counselling and feedback was administered [17, 35], and others used the mobile app to implement and deliver feedback and/or counselling functionality [15, 18, 23]. While most of the feedback was related to PA [2, 3, 5, 9, 10, 13, 18, 19, 22, 23, 26, 27, 30, 34, 35], others focused on general health [5, 6] weight [15], diet [1], and the promotion of lifestyle changes [17].

Researchers have also used messages with differing content as part of the interventions (29.73%). Most often, the use of text messages or emails is related to motivational content that promotes general health [1, 3, 14, 16, 26] or PA [1, 4, 21]. For example, Gilmore, Klempel [15] delivered personalized treatment advice remotely through phone conversations, email, and text messages. Less use was made of messages of encouragement on achieving goals [5, 18] or offering fitness challenges [25].

The other intervention components were related to self-monitoring (8/37, 21.62%), social support (6/27, 16.22%), and an “other” category (4/37, 10.81%). In addition to the self-monitoring with the Fitbit device, some studies used connected scales to monitor weight [15-17, 19]. In others, participants were required to monitor their diet [6, 30] or to write a daily diary on their PA [6, 28, 36]. The social support category covers online and offline group activities that can be delivered on platforms such as Facebook [4], but they can also be delivered at in-person group meetings [19, 22, 29] and through interpersonal behaviors [13, 24]. Further intervention components included financial incentives [8], access to a game platform [32], home rehabilitation programs [29], and access to a nutrition program, as well as weekly personal training sessions [12].

Besides differing in terms of intervention components, less than half of the Fitbit-based interventions (16/37, 43.24%) reported that their intervention was grounded in a theory, model or framework [4-6, 8, 10, 11, 13, 14, 16, 18, 21-24, 30, 36]. A wide range of theories were used to ground the interventions. Authors mostly used the social cognitive theory [6, 10, 21-24, 30] and the self-determination theory [4, 16, 18, 23]. Fewer studies based their intervention on the theory of self-regulation [22] and the transtheoretical model of behavior change [11].

References

1. Cheung, N.W., et al., *A Pilot Randomised Controlled Trial of a Text Messaging Intervention with Customisation Using Linked Data from Wireless Wearable Activity Monitors to Improve Risk Factors Following Gestational Diabetes.* Nutrients, 2019. **11**(3). [doi: 10.3390/nu11030590], [PMID: 30862052]

2. Christiansen, M.B., et al., *The feasibility and preliminary outcomes of a physical therapist-administered physical activity intervention after total knee replacement.* Arthritis Care Res (Hoboken), 2020. [doi: 10.1002/acr.23882], [PMID: 30908867]

3. Duscha, B.D., et al., *Effects of a 12-week mHealth program on peak VO2 and physical activity patterns after completing cardiac rehabilitation: A randomized controlled trial.* American Heart Journal, 2018. **199**: p. 105-114. [doi: 10.1016/j.ahj.2018.02.001], [PMID: 29754647]

4. Mendoza, J.A., et al., *A Fitbit and Facebook mHealth intervention for promoting physical activity among adolescent and young adult childhood cancer survivors: A pilot study.* Pediatr Blood Cancer, 2017. **64**(12). [doi: 10.1002/pbc.26660]

5. Amorim, A.B., et al., *Integrating Mobile-health, health coaching, and physical activity to reduce the burden of chronic low back pain trial (IMPACT): a pilot randomised controlled trial.* BMC Musculoskelet Disord, 2019. **20**(1): p. 71. [doi: 10.1186/s12891-019-2454-y], [PMID: 30744606]

6. Brown, J.C., et al., *Randomized trial of a clinic-based weight loss intervention in cancer survivors.* Journal of Cancer Survivorship, 2018. **12**(2): p. 186-195. [doi: 10.1007/s11764-017-0657-5], [PMID: 29101711]

7. Farnell, G. and J. Barkley, *The effect of a wearable physical activity monitor (Fitbit One) on physical activity behaviour in women: A pilot study.* Journal of Human Sport and Exercise, 2017. **12**. [doi: 10.14198/jhse.2017.124.09]

8. Finkelstein, E.A., et al., *Effectiveness of activity trackers with and without incentives to increase physical activity (TRIPPA): a randomised controlled trial.* The Lancet Diabetes and Endocrinology, 2016. **4**(12): p. 983-995. [doi: 10.1016/S2213-8587(16)30284-4]

9. Li, L.C., et al., *A Community-Based Physical Activity Counselling Program for People With Knee Osteoarthritis: Feasibility and Preliminary Efficacy of the Track-OA Study.* JMIR Mhealth Uhealth, 2017. **5**(6): p. e86. [doi: 10.2196/mhealth.7863], [PMID: 28652228]

10. Li, L.C., et al., *Efficacy of a Community-Based Technology-Enabled Physical Activity Counseling Program for People With Knee Osteoarthritis: Proof-of-Concept Study.* J Med Internet Res, 2018. **20**(4): p. e159. [doi: 10.2196/jmir.8514], [PMID: 29712630]

11. Miragall, M., et al., *Increasing physical activity through an Internet-based motivational intervention supported by pedometers in a sample of sedentary students: A randomised controlled trial.* Psychology & Health, 2018. **33**(4): p. 465-482. [doi: 10.1080/08870446.2017.1368511], [PMID: 28880576]

12. Thorndike, A.N., et al., *Activity monitor intervention to promote physical activity of physicians-in-training: randomized controlled trial.* Plos one, 2014. **9**(6): p. e100251. [doi: 10.1371/journal.pone.0100251], [PMID: 24950218]

13. Cadmus-Bertram, L., et al., *Building a physical activity intervention into clinical care for breast and colorectal cancer survivors in Wisconsin: a randomized controlled pilot trial.* J Cancer Surviv, 2019. [doi: 10.1007/s11764-019-00778-6], [PMID: 31264183]

14. Van Blarigan, E.L., et al., *Self-monitoring and reminder text messages to increase physical activity in colorectal cancer survivors (Smart Pace): a pilot randomized controlled trial.* BMC Cancer, 2019. **19**(1): p. 218. [doi: 10.1186/s12885-019-5427-5], [PMID: 30866859]

15. Gilmore, L.A., et al., *Personalized Mobile Health Intervention for Health and Weight Loss in Postpartum Women Receiving Women, Infants, and Children Benefit: A Randomized Controlled Pilot Study.* Journal of Women's Health, 2017. **26**(7): p. 719-727. [doi: 10.1089/jwh.2016.5947], [PMID: 28338403]

16. Griauzde, D., et al., *A Mobile Phone-Based Program to Promote Healthy Behaviors Among Adults With Prediabetes Who Declined Participation in Free Diabetes Prevention Programs: Mixed-Methods Pilot Randomized Controlled Trial.* JMIR Mhealth Uhealth, 2019. **7**(1): p. e11267. [doi: 10.2196/11267], [PMID: 30626566]

17. Redman, L.M., et al., *Effectiveness of SmartMoms, a Novel eHealth Intervention for Management of Gestational Weight Gain: Randomized Controlled Pilot Trial.* JMIR Mhealth Uhealth, 2017. **5**(9): p. e133. [doi: 10.2196/mhealth.8228], [PMID: 28903892]

18. Simons, D., et al., *Effect and Process Evaluation of a Smartphone App to Promote an Active Lifestyle in Lower Educated Working Young Adults: Cluster Randomized Controlled Trial.* JMIR Mhealth Uhealth, 2018. **6**(8): p. e10003. [doi: 10.2196/10003], [PMID: 30143477]

19. Azar, K.M.J., et al., *The Electronic CardioMetabolic Program (eCMP) for Patients With Cardiometabolic Risk: A Randomized Controlled Trial.* J Med Internet Res, 2016. **18**(5): p. e134. [doi: 10.2196/jmir.5143], [PMID: 27234480]

20. Ball, C.A., et al., *Impact of Digital Health Methods for Weight Management on Atherosclerotic Cardiovascular Disease Risk in “at-risk” Women.* Canadian Journal of Cardiology, 2016. **32**(4): p. S9-S10. [doi: 10.1016/j.cjca.2016.02.026]

21. Kooiman, T.J.M., et al., *Self-tracking of Physical Activity in People With Type 2 Diabetes: A Randomized Controlled Trial.* CIN: Computers, Informatics, Nursing, 2018. **36**(7): p. 340-349. [doi: 10.1097/CIN.0000000000000443], [PMID: 29742550]

22. McDermott, M.M., et al., *Effect of a Home-Based Exercise Intervention of Wearable Technology and Telephone Coaching on Walking Performance in Peripheral Artery Disease: The HONOR Randomized Clinical Trial.* Jama, 2018. **319**(16): p. 1665-1676. [doi: 10.1001/jama.2018.3275], [PMID: 29710165]

23. Vandelanotte, C., et al., *The Effectiveness of a Web-Based Computer-Tailored Physical Activity Intervention Using Fitbit Activity Trackers: Randomized Trial.* Journal of medical Internet research, 2018. **20**(12): p. e11321. [doi: 10.2196/11321], [PMID: 30563808]

24. Ashe, M.C., et al., *"Not just another walking program": Everyday Activity Supports You (EASY) model-a randomized pilot study for a parallel randomized controlled trial.* Pilot Feasibility Stud, 2015. **1**: p. 4. [doi: 10.1186/2055-5784-1-4]

25. DiFrancisco-Donoghue, J., et al., *Utilizing wearable technology to increase physical activity in future physicians: A randomized trial.* Preventive Medicine Reports, 2018. **12**: p. 122-127. [doi: 10.1016/j.pmedr.2018.09.004], [PMID: 30234000

26. Hartman, S.J., et al., *Randomized controlled trial of increasing physical activity on objectively measured and self-reported cognitive functioning among breast cancer survivors: The memory & motion study.* Cancer, 2018. **124**(1): p. 192-202. [doi: 10.1002/cncr.30987], [PMID: 28926676]

27. Hornikx, M., et al., *The effects of a physical activity counseling program after an exacerbation in patients with Chronic Obstructive Pulmonary Disease: a randomized controlled pilot study.* BMC Pulm Med, 2015. **15**: p. 136. [doi: 10.1186/s12890-015-0126-8]

28. Katz, P., et al., *Physical Activity to Reduce Fatigue in Rheumatoid Arthritis: A Randomized Controlled Trial.* Arthritis Care Res (Hoboken), 2018. **70**(1): p. 1-10. [doi: 10.1002/acr.23230], [PMID: 28378441]

29. Paxton, R.J., et al., *A Feasibility Study for Improved Physical Activity After Total Knee Arthroplasty.* J Aging Phys Act, 2018. **26**(1): p. 7-13. [doi: 10.1123/japa.2016-0268], [PMID: 28338406]

30. Hartman, S.J., et al., *Technology- and Phone-Based Weight Loss Intervention: Pilot RCT in Women at Elevated Breast Cancer Risk.* Am J Prev Med, 2016. **51**(5): p. 714-721. [doi: 10.1016/j.amepre.2016.06.024], [PMID: 27593420]

31. Oliveira, J.S., et al., *A combined physical activity and fall prevention intervention improved mobility-related goal attainment but not physical activity in older adults: a randomised trial.* Journal of Physiotherapy (Elsevier), 2019. **65**(1): p. 16-22. [doi: 10.1016/j.jphys.2018.11.005], [PMID: 30581138]

32. Jennings, F.H., et al., *Promote Students’ Healthy Behavior Through Sensor and Game: A Randomized Controlled Trial.* Medical Science Educator, 2016. **26**(3): p. 349-355. [doi: 10.1007/s40670-016-0253-8]

33. Lystrup, R.M., et al., *Pedometry to Prevent Cardiorespiratory Fitness Decline-Is it Effective?* Military medicine, 2016. **181**(10): p. 1235‐1239. [doi: 10.7205/MILMED-D-15-00540], [PMID: 27753558]

34. Thompson, W.G., et al., *“Go4Life” exercise counseling, accelerometer feedback, and activity levels in older people.* Archives of Gerontology and Geriatrics, 2014. **58**(3): p. 314-319. [doi: 10.1016/j.archger.2014.01.004], [PMID: 24485546]

35. Shoemaker, M.J., et al., *Exercise- and Psychosocial-Based Interventions to Improve Daily Activity in Heart Failure: A Pilot Study.* Home Health Care Management & Practice, 2016. **29**(2): p. 111-120. [doi: 10.1177/1084822316683660]

36. Eisenberg, M.H., et al., *The impact of E-diaries and accelerometers on young adults' perceived and objectively assessed physical activity.* Psychology of Sport & Exercise, 2017. **30**: p. 55-63. [doi: 10.1016/j.psychsport.2017.01.008]
