# Supplementary material for: Fitbit-Based Interventions for Healthy Lifestyle Outcomes: Systematic Review and Meta-Analysis
Source: J Med Internet Res. 2020 Oct 12;22(10):e23954. doi: 10.2196/23954 (PMC7589007; doi:10.2196/23954)
Supplement: Multimedia Appendix 6 [file jmir_v22i10e23954_app6.docx]

Outcomes and how they were reported in the included studies

| Clusters of outcomes | Outcomes | References |
| --- | --- | --- |
| **Steps** | Steps (counts per day) | [1-18] |
|  | Steps (counts per week) | [7, 19-22] |
|  | Meeting ≥10 000 steps target (per day) | [20, 23] |
|  | Healthy activity level of ≥ 8000 steps (per day) | [21] |
|  | Meeting ≥70,000 steps target (per week) | [7] |
| **MVPA** | MVPA (min/day) | [1, 16, 18, 24-27] |
|  | MVPA (min/week) | [2, 3, 7, 19, 28, 29] |
|  | Vigorous PA (min/week) | [2, 6, 7, 9, 19] |
|  | Moderate PA (min/week) | [2, 6, 9, 19] |
|  | Meeting ≥150 min of moderate PA (per week) | [7, 14, 20] |
|  | MVPA in bouts (min/week) | [2, 7] |
|  | Moderate PA (min/day) | [16, 18] |
|  | MVPA (in bouts per day) ≥3 METS | [11, 30] |
|  | MVPA (in bouts per day) ≥4 METS | [11, 30] |
|  | Vigorous PA (min/day) | [16, 18] |
|  | Meeting ≥ 30 min of MVPA (per day) | [10] |
|  | Meeting 150 min/week (No.) | [26] |
|  | Moderate Activity (hour/day) | [31] |
|  | Moderate-High activity (min/day) | [5] |
|  | Moderate-High activity (min/week) | [5] |
|  | Moderate-low activity (min/day) | [5] |
|  | Moderate-low activity (min/week) | [5] |
|  | MVPA (10 –min bouts/day) | [25] |
|  | Vigorous activity (hour/day) | [31] |
| **LPA** | Light PA (min/week) | [2, 7, 9, 19] |
|  | Light activity (hour/day) | [31] |
|  | Light PA (min/day) | [16] |
|  | Low Activity (min/day) | [5] |
|  | Low Activity (min/week) | [5] |
|  | Mild PA (min/week) | [6] |
| **Sedentary Behavior** | Sedentary behavior (min/day) | [1, 18, 24, 27] |
|  | Sedentary behavior (%/day) | [1, 32] |
|  | Sedentary behavior (bouts in min) | [11, 30] |
|  | Sedentary activity (%) | [31] |
|  | Sedentary activity (< 5000 steps per day) | [21] |
|  | Sedentary time (min/week) | [7] |
|  | Sitting (min/day) | [29] |
|  | Prolonged sedentary 30 min-bouts (%/day) | [32] |
| **Other PA-related** | Distance covered in 6-min walk test (m) | [15, 22, 28, 31] |
|  | Total active time (min/week) | [5, 16, 29] |
|  | PA enjoyment | [12, 13, 27] |
|  | Total active time (min/day) | [5, 16] |
|  | Time for up-and-go test (s) | [15, 33] |
|  | Distance (miles/day) | [5] |
|  | Distance (miles/week) | [5] |
|  | Distance walked during past week | [22] |
|  | Floors climbed (per day) | [5] |
|  | Floors climbed (per week) | [5] |
|  | Time for four-meter walk test (s) | [15] |
|  | Functional exercise capacity | [8] |
|  | Physical activity | [12] |
|  | Physical activity (metabolic equivalents) | [34] |
|  | Physical activity (min/week) | [28] |
|  | Physical activity score | [35] |
|  | Physical functioning | [22] |
|  | Occupational PA (min/week) | [16] |
|  | PA activity (hour/day) | [31] |
|  | PA goal attainment (%) | [19] |
|  | PA participation (counts/min) | [14] |
|  | Total PA (METS) | [6] |
|  | Total weekly activity time (min) | [20] |
|  | Household PA (min/week) | [16] |
|  | 1.5-Mile Run (min:sec) | [23] |
|  | 2-Mile Run (min:sec) | [23] |
|  | 30-second timed chair rise (repetitions) | [31] |
|  | Active transport (min/week) | [16] |
|  | Activity units per day | [33] |
|  | Days exercised | [4] |
|  | Walking (min/week) | [19] |
|  | Walking exercise frequency during past week | [22] |
|  | Walking time (min/day) | [8] |
|  | WIQ score on distance, speed and stair climbing | [22] |
|  | Recreational PA (min/week) | [16] |
|  | Stage of PA behavior | [13] |
|  | Total activity (min/day) | [26] |
|  | Discrete exercise sessions | [6] |
|  | Movement intensity during walking (m/s^2^/day) | [8] |
|  | Attitude toward engagement in exercise | [10] |
|  | Decision-making related to PA | [13] |
|  | Intention toward engagement in exercise | [13] |
|  | Motivation for PA | [27] |
|  | Processes of change to practice PA | [13] |
|  | Self-determined motivation for PA | [12] |
|  | Social norm toward engagement in exercise | [10] |
|  | Social support for PA | [16] |
| **Weight** | Weight (kg) | [1, 4, 7, 10, 20, 25, 33, 34, 36-38] |
|  | BMI (kg/m^2^) | [4, 10, 17, 26, 29, 34] |
|  | Waist circumference (cm) | [10, 33, 34, 37] |
|  | Body fat (%) | [4, 33, 37] |
|  | Fat mass (kg) | [4, 28] |
|  | Hip circumference (cm) | [10, 37] |
|  | Lean Mass (kg) | [4, 28] |
|  | Body mass (kg) | [28] |
|  | Weight (lbs) | [17] |
|  | Weight loss (%) | [25] |
|  | Waist (inches) | [17] |
| **Intake** | Fiber (g/day) | [20, 28] |
|  | Caloric intake (kcal/day) | [28] |
|  | Carbohydrate (% kcal/day) | [28] |
|  | Change in total daily energy (kj) | [20] |
|  | Daily carbohydrate intake (% total energy) | [20] |
|  | Daily fat intake (% total energy) | [20] |
|  | Daily saturated fat intake (% total energy) | [20] |
|  | Fruits (servings/day) | [28] |
|  | Meeting diet fat intake <30% of total energy (No.) | [20] |
|  | Diet quality | [34] |
|  | Meeting dietary fiber >15 g/1000 cal (No.) | [20] |
|  | Fat % (kcal/day) | [28] |
|  | Total daily energy intake (kj) | [20] |
|  | Meeting sat fat <10% of total energy (No.) | [20] |
|  | Vegetables (servings/day) | [28] |
| **Oxygen Uptake** | Cardiorespiratory fitness VO2max in non-exercising conditions s(mL/kg/min) | [7] |
|  | Peak VO2 (L/min) | [5] |
|  | Peak VO2 (ml/kg/min) | [5] |
| **Sleep** | Sleep quality | [9] |
|  | Sleep time (min/day) | [9] |
| **Cognition one's health** | Health (scale) | [1, 10, 31] |
|  | Motivation to prevent type 2 diabetes mellitus | [39] |
|  | Stress | [9] |
|  | Stress and Mental health | [34] |
| **Self-efficacy** | Self-efficacy | [13, 16] |
|  | Self-efficacy toward engagement in exercise | [10] |
| **Quality of life** | Quality of life | [7, 14] |
|  | Health-related quality of life | [34] |

References

1. Ashe, M.C., et al., *"Not just another walking program": Everyday Activity Supports You (EASY) model-a randomized pilot study for a parallel randomized controlled trial.* Pilot Feasibility Stud, 2015. **1**: p. 4.

2. Cadmus-Bertram, L., et al., *Building a physical activity intervention into clinical care for breast and colorectal cancer survivors in Wisconsin: a randomized controlled pilot trial.* J Cancer Surviv, 2019.

3. Christiansen, M.B., et al., *The feasibility and preliminary outcomes of a physical therapist-administered physical activity intervention after total knee replacement.* Arthritis Care Res (Hoboken), 2019.

4. DiFrancisco-Donoghue, J., et al., *Utilizing wearable technology to increase physical activity in future physicians: A randomized trial.* Preventive Medicine Reports, 2018. **12**: p. 122-127.

5. Duscha, B.D., et al., *Effects of a 12-week mHealth program on peak VO2 and physical activity patterns after completing cardiac rehabilitation: A randomized controlled trial.* American Heart Journal, 2018. **199**: p. 105-114.

6. Eisenberg, M.H., et al., *The impact of E-diaries and accelerometers on young adults' perceived and objectively assessed physical activity.* Psychology of Sport & Exercise, 2017. **30**: p. 55-63.

7. Finkelstein, E.A., et al., *Effectiveness of activity trackers with and without incentives to increase physical activity (TRIPPA): a randomised controlled trial.* The Lancet Diabetes and Endocrinology, 2016. **4**(12): p. 983-995.

8. Hornikx, M., et al., *The effects of a physical activity counseling program after an exacerbation in patients with Chronic Obstructive Pulmonary Disease: a randomized controlled pilot study.* BMC Pulm Med, 2015. **15**: p. 136.

9. Jennings, F.H., et al., *Promote Students’ Healthy Behavior Through Sensor and Game: A Randomized Controlled Trial.* Medical Science Educator, 2016. **26**(3): p. 349-355.

10. Kooiman, T.J.M., et al., *Self-tracking of Physical Activity in People With Type 2 Diabetes: A Randomized Controlled Trial.* CIN: Computers, Informatics, Nursing, 2018. **36**(7): p. 340-349.

11. Li, L.C., et al., *Efficacy of a Community-Based Technology-Enabled Physical Activity Counseling Program for People With Knee Osteoarthritis: Proof-of-Concept Study.* J Med Internet Res, 2018. **20**(4): p. e159.

12. Mahar, M.T., et al., *Effects of an Intervention using Movement Technology in a University Physical Activity Class: 1902 Board #247 May 28, 330 PM - 500 PM.* Medicine & Science in Sports & Exercise, 2015. **47**(5S): p. 522.

13. Miragall, M., et al., *Increasing physical activity through an Internet-based motivational intervention supported by pedometers in a sample of sedentary students: A randomised controlled trial.* Psychology & Health, 2018. **33**(4): p. 465-482.

14. Oliveira, J.S., et al., *A combined physical activity and fall prevention intervention improved mobility-related goal attainment but not physical activity in older adults: a randomised trial.* Journal of Physiotherapy (Elsevier), 2019. **65**(1): p. 16-22.

15. Paxton, R.J., et al., *A Feasibility Study for Improved Physical Activity After Total Knee Arthroplasty.* J Aging Phys Act, 2018. **26**(1): p. 7-13.

16. Simons, D., et al., *Effect and Process Evaluation of a Smartphone App to Promote an Active Lifestyle in Lower Educated Working Young Adults: Cluster Randomized Controlled Trial.* JMIR Mhealth Uhealth, 2018. **6**(8): p. e10003.

17. Thorndike, A.N., et al., *Activity monitor intervention to promote physical activity of physicians-in-training: randomized controlled trial.* Plos one, 2014. **9**(6): p. e100251.

18. Van Blarigan, E.L., et al., *Self-monitoring and reminder text messages to increase physical activity in colorectal cancer survivors (Smart Pace): a pilot randomized controlled trial.* BMC Cancer, 2019. **19**(1): p. 218.

19. Amorim, A.B., et al., *Integrating Mobile-health, health coaching, and physical activity to reduce the burden of chronic low back pain trial (IMPACT): a pilot randomised controlled trial.* BMC Musculoskelet Disord, 2019. **20**(1): p. 71.

20. Cheung, N.W., et al., *A Pilot Randomised Controlled Trial of a Text Messaging Intervention with Customisation Using Linked Data from Wireless Wearable Activity Monitors to Improve Risk Factors Following Gestational Diabetes.* Nutrients, 2019. **11**(3).

21. Katz, P., et al., *Physical Activity to Reduce Fatigue in Rheumatoid Arthritis: A Randomized Controlled Trial.* Arthritis Care Res (Hoboken), 2018. **70**(1): p. 1-10.

22. McDermott, M.M., et al., *Effect of a Home-Based Exercise Intervention of Wearable Technology and Telephone Coaching on Walking Performance in Peripheral Artery Disease: The HONOR Randomized Clinical Trial.* Jama, 2018. **319**(16): p. 1665-1676.

23. Lystrup, R.M., et al., *Pedometry to Prevent Cardiorespiratory Fitness Decline-Is it Effective?* Military medicine, 2016. **181**(10): p. 1235‐1239.

24. Falck, R.S., et al., *Can we improve cognitive function among adults with osteoarthritis by increasing moderate-to-vigorous physical activity and reducing sedentary behaviour? Secondary analysis of the MONITOR-OA study.* BMC Musculoskelet Disord, 2018. **19**(1): p. 447.

25. Hartman, S.J., et al., *Technology- and Phone-Based Weight Loss Intervention: Pilot RCT in Women at Elevated Breast Cancer Risk.* Am J Prev Med, 2016. **51**(5): p. 714-721.

26. Hartman, S.J., et al., *Randomized controlled trial of increasing physical activity on objectively measured and self-reported cognitive functioning among breast cancer survivors: The memory & motion study.* Cancer, 2018. **124**(1): p. 192-202.

27. Mendoza, J.A., et al., *A Fitbit and Facebook mHealth intervention for promoting physical activity among adolescent and young adult childhood cancer survivors: A pilot study.* Pediatr Blood Cancer, 2017. **64**(12).

28. Brown, J.C., et al., *Randomized trial of a clinic-based weight loss intervention in cancer survivors.* Journal of Cancer Survivorship, 2018. **12**(2): p. 186-195.

29. Vandelanotte, C., et al., *The Effectiveness of a Web-Based Computer-Tailored Physical Activity Intervention Using Fitbit Activity Trackers: Randomized Trial.* Journal of medical Internet research, 2018. **20**(12): p. e11321.

30. Li, L.C., et al., *A Community-Based Physical Activity Counselling Program for People With Knee Osteoarthritis: Feasibility and Preliminary Efficacy of the Track-OA Study.* JMIR Mhealth Uhealth, 2017. **5**(6): p. e86.

31. Shoemaker, M.J., et al., *Exercise- and Psychosocial-Based Interventions to Improve Daily Activity in Heart Failure: A Pilot Study.* Home Health Care Management & Practice, 2016. **29**(2): p. 111-120.

32. Sloan, R.A., et al., *The influence of a consumer-wearable activity tracker on sedentary time and prolonged sedentary bouts: secondary analysis of a randomized controlled trial.* BMC research notes, 2018. **11**(1): p. 189.

33. Thompson, W.G., et al., *“Go4Life” exercise counseling, accelerometer feedback, and activity levels in older people.* Archives of Gerontology and Geriatrics, 2014. **58**(3): p. 314-319.

34. Azar, K.M.J., et al., *The Electronic CardioMetabolic Program (eCMP) for Patients With Cardiometabolic Risk: A Randomized Controlled Trial.* J Med Internet Res, 2016. **18**(5): p. e134.

35. Farnell, G. and J. Barkley, *The effect of a wearable physical activity monitor (Fitbit One) on physical activity behaviour in women: A pilot study.* Journal of Human Sport and Exercise, 2017. **12**.

36. Ball, C.A., et al., *Impact of Digital Health Methods for Weight Management on Atherosclerotic Cardiovascular Disease Risk in “at-risk” Women.* Canadian Journal of Cardiology, 2016. **32**(4): p. S9-S10.

37. Gilmore, L.A., et al., *Personalized Mobile Health Intervention for Health and Weight Loss in Postpartum Women Receiving Women, Infants, and Children Benefit: A Randomized Controlled Pilot Study.* Journal of Women's Health, 2017. **26**(7): p. 719-727.

38. Redman, L.M., et al., *Effectiveness of SmartMoms, a Novel eHealth Intervention for Management of Gestational Weight Gain: Randomized Controlled Pilot Trial.* JMIR Mhealth Uhealth, 2017. **5**(9): p. e133.

39. Griauzde, D., et al., *A Mobile Phone-Based Program to Promote Healthy Behaviors Among Adults With Prediabetes Who Declined Participation in Free Diabetes Prevention Programs: Mixed-Methods Pilot Randomized Controlled Trial.* JMIR Mhealth Uhealth, 2019. **7**(1): p. e11267.
