# Supplementary material for: Fitbit-Based Interventions for Healthy Lifestyle Outcomes: Systematic Review and Meta-Analysis
Source: J Med Internet Res. 2020 Oct 12;22(10):e23954. doi: 10.2196/23954 (PMC7589007; doi:10.2196/23954)
Supplement: Multimedia Appendix 7 [file jmir_v22i10e23954_app7.pdf]

Characteristics of studies

Characteristics of included studies

Amorim, 2019

**Methods**	
**Participants**	
**Interventions**	
**Outcomes**	
**Notes**	

Risk of bias table

Bias	Authors' judgement	Support for judgement
Random sequence generation (selection bias)	Low risk	“[…] using a computer-generated random number schedule of 10 permuted blocks of 6 and the final block of 8.” (p. 3)
Allocation concealment (selection bias)	Low risk	“To ensure allocation concealment, randomization to groups was undertaken by a blinded remote investigator (MS) not involved in recruitment […]” (p.3). It is a central allocation.
Blinding of participants and personnel (performance bias)	High risk	Due to the nature of the intervention and control conditions make blinding impossible.
Blinding of outcome assessment (detection bias)	Low risk	“We conducted a pilot randomized controlled trial with blinded outcome assessment.” (p. 2)<br>"Study investigators conducting data collection were blinded to group allocation" (p. 3)
Incomplete outcome data (attrition bias)	Low risk	“Overall, there were 20% of missing data at the 6-month questionnaire follow-up and 16% of missing data across the 6-month weekly surveys.” (p. 7). The reasons for missing data are not related to true outcome (p. 7) but they just mentioned they analyzed data by “intention to treat” (p. 6)
Selective reporting (reporting bias)	Low risk	The study protocol is available and all of the study’s pre-specified (primary and secondary) outcomes that are of interest in the review have been reported in the pre-specified way.
Other bias	Low risk	The study appears to be free of other sources of bias.

Ashe, 2015

**Methods**	
**Participants**	
**Interventions**	
**Outcomes**	
**Notes**	

Risk of bias table

Bias	Authors' judgement	Support for judgement
Random sequence generation (selection bias)	Low risk	"An independent statistical consultant set up the web-based randomization process to assign eligible participants to intervention or control groups by remote allocation, using permuted blocks of size 2 and 4" (p. 3/12)
Allocation concealment (selection bias)	Low risk	"An independent statistical consultant set up the web-based randomization process to assign eligible participants to intervention or control groups by remote allocation, using permuted blocks of size 2 and 4" (p. 3/12)<br>"No one directly involved in the project had access to allocation codes" (p. 3/12)
Blinding of participants and personnel (performance bias)	High risk	Due to the nature of the intervention and control conditions make blinding impossible.
Blinding of outcome assessment (detection bias)	Low risk	"Only those who did not deliver the intervention (measurement team) were blinded to group allocation" (p. 3/12)
Incomplete outcome data (attrition bias)	Low risk	Reasons for missing outcome data unlikely to be related to true outcome: "[...] control participant's injury prevented her from wearing the accelerometer at the final assessment" (p. 6/12)
Selective reporting (reporting bias)	Low risk	The study protocol is available and all of the study’s pre-specified (primary and secondary) outcomes that are of interest in the review have been reported in the pre-specified way.
Other bias	Low risk	The study appears to be free of other sources of bias.

Azar, 2016

**Methods**	
**Participants**	
**Interventions**	
**Outcomes**	
**Notes**	

Risk of bias table

Bias	Authors' judgement	Support for judgement
Random sequence generation (selection bias)	Low risk	“We applied our published dynamic block randomization method […]” (p. 3)
Allocation concealment (selection bias)	Low risk	“The method automatically ensures allocation concealment.” (p. 3)

Blinding of participants and personnel (performance bias)	High risk	Due to the nature of the intervention and control conditions make blinding impossible.
Blinding of outcome assessment (detection bias)	Low risk	“While study group assignment was identifiable to participants and interventionists, blinding was otherwise maintained for data collection, outcome adjudication, and data analysis.” (p. 3)
Incomplete outcome data (attrition bias)	Low risk	No reason for missing data were provided. Missing data for outcome were balanced accross both groups and missing data were not big enough to have a clinically impact on the observed effect size.
Selective reporting (reporting bias)	Low risk	The study protocol is available and all of the study’s pre-specified (primary and secondary) outcomes that are of interest in the review have been reported in the pre-specified way.
Other bias	Low risk	The study appears to be free of other sources of bias.

Ball, 2016

**Methods**	
**Participants**	
**Interventions**	
**Outcomes**	
**Notes**	

Risk of bias table

**Bias**	**Authors' judgement**	**Support for judgement**
Random sequence generation (selection bias)	Unclear risk	Insufficient information about the sequence generation process to permit judgement of ‘Yes’ or ‘No’.
Allocation concealment (selection bias)	Unclear risk	Insufficient information to permit judgement of ‘Yes’ or ‘No’.
Blinding of participants and personnel (performance bias)	High risk	Due to the nature of the intervention and control conditions make blinding impossible.
Blinding of outcome assessment (detection bias)	Unclear risk	Insufficient information to permit judgement of ‘Yes’ or ‘No’.
Incomplete outcome data (attrition bias)	Unclear risk	"Eighty-nine women with 2 CVD risk factors were prospectively randomized"<br>“Fifty-eight women (DH=30, C=28) completed baseline and 3-month testing.”<br>There is missing data but unclear whether missing data were balanced accross the groups.
Selective reporting (reporting bias)	Low risk	The study protocol is not available but it is clear that the published reports include all expected outcomes, including those that were pre-specified.

Other bias	Low risk	The study appears to be free of other sources of bias.

Brown, 2018

**Methods**	
**Participants**	
**Interventions**	
**Outcomes**	
**Notes**	

Risk of bias table

**Bias**	**Authors' judgement**	**Support for judgement**
Random sequence generation (selection bias)	Low risk	"Participants were randomly allocated to 1:1 ratio using computer allocation [...]" (p. 187).
Allocation concealment (selection bias)	Low risk	Participants and investigators enrolling participants could not foresee assignment because of central allocation: " [...] computer allocation to either a weight loss intervention or wait-list control group" (p. 187)
Blinding of participants and personnel (performance bias)	High risk	Due to the nature of the intervention and control conditions make blinding impossible.
Blinding of outcome assessment (detection bias)	Low risk	"Outcome measures were obtained by assessors blinded to treatment assignment." (p. 187)
Incomplete outcome data (attrition bias)	Low risk	"Sensitivity analyses were conducted on all outcomes using baseline observation carried forward imputation and linear mixed-effect regression modeling [24]." (p. 189)
Selective reporting (reporting bias)	Low risk	The study protocol is not available but it is clear that the published reports include all expected outcomes, including those that were pre-specified.
Other bias	Low risk	The study appears to be free of other sources of bias.

Cadmus-Bertram, 2019

**Methods**	
**Participants**	
**Interventions**	
**Outcomes**	
**Notes**	

Risk of bias table

Bias	Authors' judgement	Support for judgement
Random sequence generation (selection bias)	Low risk	“After completion of baseline measures, a computerized randomization scheme in REDCap [35] randomly assigned each dyad with equal probability to either the intervention group or the comparison group.” (p. 3)
Allocation concealment (selection bias)	Low risk	Participants and investigators enrolling participants could not foresee assignment because of central allocation (REDCap). (p.3)
Blinding of participants and personnel (performance bias)	High risk	Due to the nature of the intervention and control conditions make blinding impossible.
Blinding of outcome assessment (detection bias)	Low risk	Blinding of outcomes assessment was not mentioned but the outcome measurement is not likely to be influenced by lack of blinding because outcomes were objectively measured.
Incomplete outcome data (attrition bias)	Low risk	No reason for missing data were provided. Missing data for outcome were balanced accross both groups and missing data were not big enough to have a clinically impact on the observed effect size.
Selective reporting (reporting bias)	Low risk	The study protocol is available and all of the study’s pre-specified (primary and secondary) outcomes that are of interest in the review have been reported in the pre-specified way.
Other bias	Low risk	The study appears to be free of other sources of bias.

Cheung, 2019

**Methods**	
**Participants**	
**Interventions**	
**Outcomes**	
**Notes**	

Risk of bias table

Bias	Authors' judgement	Support for judgement
Random sequence generation (selection bias)	Low risk	“Randomization was undertaken by computer random number generation, using a permuted bloc size of 4.” (p. 3)
Allocation concealment (selection bias)	Unclear risk	Insufficient information to permit judgement of ‘Yes’ or ‘No’.
Blinding of participants and personnel (performance bias)	High risk	Due to the nature of the intervention and control conditions make blinding impossible.

Blinding of outcome assessment (detection bias)	Low risk	Blinding of outcomes assessment was not mentioned but the outcome measurement is not likely to be influenced by lack of blinding because outcomes were objectively measured.
Incomplete outcome data (attrition bias)	High risk	"During the course of the intervention, 27 intervention subjects had at least one problem related to the activity monitor. There were 4 occasions when subjects lost their activity monitors, 18 occasions where the wristband or clip was lost or damaged, 6 when the charger was lost, and 26 where the activity monitor required a factory settings or password reset. Although all lost or damaged items were replaced, these mishaps resulted in disruption to the study and periods where the activity monitor was not worn" (p. 9)
Selective reporting (reporting bias)	Low risk	The study protocol is available and all of the study’s pre-specified (primary and secondary) outcomes that are of interest in the review have been reported in the pre-specified way.
Other bias	Low risk	The study appears to be free of other sources of bias.

Christiansen, 2019

**Methods**	
**Participants**	
**Interventions**	
**Outcomes**	
**Notes**	

Risk of bias table

**Bias**	**Authors' judgement**	**Support for judgement**
Random sequence generation (selection bias)	Unclear risk	Insufficient information about the sequence generation process to permit judgement of ‘Yes’ or ‘No’.
Allocation concealment (selection bias)	Unclear risk	“A research assistant randomized each participant using a manila envelope with note cards labeled “A” for intervention and “B” for control.” (p. 5)<br>Unclear if envelopes were sequentially numbered, opaque, and sealed.
Blinding of participants and personnel (performance bias)	High risk	Due to the nature of the intervention and control conditions make blinding impossible.
Blinding of outcome assessment (detection bias)	Low risk	"the research assistant analyzing the PA data was masked to group assignment" (p.5)
Incomplete outcome data (attrition bias)	Low risk	Missing outcome data balanced in numbers across intervention groups, with similar reasons for missing data across groups.

Selective reporting (reporting bias)	Low risk	The study protocol is available and all of the study's pre-specified (primary and secondary) outcomes that are of interest in the review have been reported in the pre-specified way.
Other bias	Low risk	The study appears to be free of other sources of bias.

DiFrancisco-Donoghue, 2018

**Methods**	
**Participants**	
**Interventions**	
**Outcomes**	
**Notes**	

Risk of bias table

**Bias**	**Authors' judgement**	**Support for judgement**
Random sequence generation (selection bias)	Unclear risk	Insufficient information about the sequence generation process to permit judgement of 'Yes' or 'No'.
Allocation concealment (selection bias)	Unclear risk	Insufficient information to permit judgement of 'Yes' or 'No'.
Blinding of participants and personnel (performance bias)	High risk	Due to the nature of the intervention and control conditions make blinding impossible.
Blinding of outcome assessment (detection bias)	Low risk	Blinding of outcomes assessment was not mentioned but the outcome measurement is not likely to be influenced by lack of blinding because outcomes were objectively measured.
Incomplete outcome data (attrition bias)	Low risk	Missing outcome data balanced in numbers across intervention groups, with similar reasons for missing data.
Selective reporting (reporting bias)	Low risk	The study protocol is available and all of the study's pre-specified (primary and secondary) outcomes that are of interest in the review have been reported in the pre-specified way.
Other bias	High risk	Significant differences between groups at baseline.

Duscha, 2018

**Methods**	
**Participants**	
**Interventions**	
**Outcomes**	
**Notes**	

Risk of bias table

Bias	Authors' judgement	Support for judgement
Random sequence generation (selection bias)	Unclear risk	Insufficient information about the sequence generation process to permit judgement of 'Yes' or 'No'.
Allocation concealment (selection bias)	Unclear risk	Insufficient information to permit judgement of 'Yes' or 'No'.
Blinding of participants and personnel (performance bias)	High risk	Due to the nature of the intervention and control conditions make blinding impossible.
Blinding of outcome assessment (detection bias)	Low risk	Blinding of outcomes assessment was not mentioned but the outcome measurement is not likely to be influenced by lack of blinding because outcomes were objectively measured.
Incomplete outcome data (attrition bias)	Low risk	Missing outcome data balanced in numbers across intervention groups, with similar reasons for missing data across groups (p. 106)
Selective reporting (reporting bias)	Low risk	The study protocol is available and all of the study's pre-specified (primary and secondary) outcomes that are of interest in the review have been reported in the pre-specified way.
Other bias	High risk	Two authors were working for Vida Health, the firm producing the Health coaching

Eisenberg, 2017

**Methods**	
**Participants**	
**Interventions**	
**Outcomes**	
**Notes**	

Risk of bias table

Bias	Authors' judgement	Support for judgement
Random sequence generation (selection bias)	Unclear risk	Insufficient information about the sequence generation process to permit judgement of 'Yes' or 'No'.
Allocation concealment (selection bias)	Unclear risk	Insufficient information to permit judgement of 'Yes' or 'No'.
Blinding of participants and personnel (performance bias)	High risk	Due to the nature of the intervention and control conditions make blinding impossible.
Blinding of outcome assessment (detection bias)	Low risk	Blinding of outcomes assessment was not mentioned but the outcome measurement is not likely to be influenced by lack of blinding because outcomes were objectively measured.

Incomplete outcome data (attrition bias)	Low risk	Attrition is balanced across groups and not enough to induce clinically relevant bias in observed effect sizes.
Selective reporting (reporting bias)	Low risk	The study protocol is not available but it is clear that the published reports include all expected outcomes.
Other bias	Low risk	The study appears to be free of other sources of bias.

Farnell, 2017

**Methods**	
**Participants**	
**Interventions**	
**Outcomes**	
**Notes**	

Risk of bias table

Bias	Authors' judgement	Support for judgement
Random sequence generation (selection bias)	Unclear risk	Insufficient information about the sequence generation process to permit judgement of ‘Yes’ or ‘No’.
Allocation concealment (selection bias)	Unclear risk	Insufficient information to permit judgement of ‘Yes’ or ‘No’.
Blinding of participants and personnel (performance bias)	High risk	Due to the nature of the intervention and control conditions make blinding impossible.
Blinding of outcome assessment (detection bias)	Unclear risk	Insufficient information to permit judgement of ‘Yes’ or ‘No’.
Incomplete outcome data (attrition bias)	Unclear risk	Insufficient reporting of attrition/exclusions to permit judgement of ‘Yes’ or ‘No’.
Selective reporting (reporting bias)	Low risk	The study protocol is not available but it is clear that the published reports include all expected outcomes, including those that were pre-specified.
Other bias	Low risk	The study appears to be free of other sources of bias.

Finkelstein, 2016

**Methods**	
**Participants**	
**Interventions**	
**Outcomes**	
**Notes**	

Risk of bias table

Bias	Authors' judgement	Support for judgement
Random sequence generation (selection bias)	Low risk	“Randomisation of groups of a particular size was done in blocks of size four to ensure approximate balance between study groups for participants of each group size. We randomly assigned groups (with 1–4 members) to one of the four study groups in a 1:1:1:1 ratio, using a computer generated assignment schedule prepared by the statistician”. (p. 985)
Allocation concealment (selection bias)	Low risk	"Randomisation envelopes containing a slip of paper indicating the study group were prepared by research staff not involved in random allocation" (p. 985).<br>“The envelopes were arranged in sequential order for each stratum (ie, groups of 1–4 members) […]” (p. 985).
Blinding of participants and personnel (performance bias)	High risk	“Because of the nature of the intervention, the participants and study coordinator could not be blinded to the study group assignment”. (p. 985)
Blinding of outcome assessment (detection bias)	Low risk	“However, team members involved in assessing outcomes in participants and data analysts were blinded to group assignment”.
Incomplete outcome data (attrition bias)	Low risk	Missing data have been imputed using appropriate methods: “All statistical analyses were based on estimates pooled across 20 multiple imputed datasets with each complete dataset based on 50 iterations of predictive mean matching imputation of each dependent variable’s missing values conditional on all other variables. Each of the 20 complete datasets was formed by looping across variables for 50 iterations. Within each iteration, each variable’s non-missing values were regressed on all other variables via stepwise variable selection using the Akaike information criterion, then noisy predictions for the variable’s missing components were generated from the regression model, and matched to their nearest nonmissing entry” (pp. 988-989).
Selective reporting (reporting bias)	Low risk	The study protocol is available and all of the study’s pre-specified primary outcomes have been reported in the pre-specified way.
Other bias	Low risk	The study appears to be free of other sources of bias.

Gilmore, 2017

**Methods**	
**Participants**	
**Interventions**	
**Outcomes**	
**Notes**	

Risk of bias table

Bias	Authors' judgement	Support for judgement
Random sequence generation (selection bias)	Unclear risk	Insufficient information about the sequence generation process to permit judgement of 'Yes' or 'No'.
Allocation concealment (selection bias)	Unclear risk	Insufficient information to permit judgement of 'Yes' or 'No'.
Blinding of participants and personnel (performance bias)	High risk	Due to the nature of the intervention and control conditions make blinding impossible.
Blinding of outcome assessment (detection bias)	Low risk	Blinding of outcomes assessment was not mentioned but the outcome measurement is not likely to be influenced by lack of blinding because outcomes were objectively measured.
Incomplete outcome data (attrition bias)	Low risk	Attrition was not enough to have a clinically relevant impact on the intervention effect estimate.
Selective reporting (reporting bias)	Low risk	The study protocol is available and all of the study's pre-specified (primary and secondary) outcomes that are of interest in the review have been reported in the pre-specified way.
Other bias	Low risk	The study appears to be free of other sources of bias.

Griauzde, 2019

**Methods**	
**Participants**	
**Interventions**	
**Outcomes**	
**Notes**	

Risk of bias table

Bias	Authors' judgement	Support for judgement
Random sequence generation (selection bias)	Low risk	"[…] were assigned to the 3 study groups using 1:1:1 central computerized randomization. The allocation sequence was generated using Stata 14" (p. 4)
Allocation concealment (selection bias)	Low risk	"A Web-based tool, the University of Michigan computerized randomization system (Treatment Assignment Tool-UM, TATUM), was used to allow for blinded treatment allocation." (p. 4)
Blinding of participants and personnel (performance bias)	High risk	Due to the nature of the intervention and control conditions make blinding impossible.

Blinding of outcome assessment (detection bias)	Low risk	Primary outcome of interest (feasibility and acceptability) were measured objectively and secondary quantitave measures were self-reported via a survey link an email address of the subjects.
Incomplete outcome data (attrition bias)	High risk	High imbalance between "App-plus" and the other two groups in lost to follow-up.<br>Plausible effect size among missing outcomes enough to induce clinically relevant bias in observed effect size.
Selective reporting (reporting bias)	Low risk	The study protocol is available and all of the study's pre-specified (primary and secondary) outcomes that are of interest in the review have been reported in the pre-specified way
Other bias	Low risk	The study appears to be free of other sources of bias.

Hartman, 2016

**Methods**	
**Participants**	
**Interventions**	
**Outcomes**	
**Notes**	

Risk of bias table

**Bias**	**Authors' judgement**	**Support for judgement**
Random sequence generation (selection bias)	Low risk	"A web-based application was used to randomly assign each participant with 2:1 probability to either the technology plus phone-based intervention group or to the usual care group." (p. 716)
Allocation concealment (selection bias)	Low risk	"Staff members who randomized participants were blinded to the allocation sequence. Participants were notified of their group assignment via phone and received intervention materials by postal mail." (p. 716)
Blinding of participants and personnel (performance bias)	High risk	Due to the nature of the intervention and control conditions make blinding impossible.
Blinding of outcome assessment (detection bias)	Low risk	Blinding of outcomes assessment was not mentioned but the outcome measurement is not likely to be influenced by lack of blinding because outcomes were objectively measured.
Incomplete outcome data (attrition bias)	Low risk	"Missing data were assumed missing at random and were accounted for in the longitudinal random effects models by using a likelihood-based estimation method, which uses all available data and does not ignore subjects with missing data. In analysis models that used only a single time point, a complete cases analysis was carried out, with those missing data dropped from the analysis."

Selective reporting (reporting bias)	Low risk	The study protocol is not available but it is clear that the published reports include all expected outcomes, including those that were pre-specified.
Other bias	Low risk	The study appears to be free of other sources of bias.

Hartman, 2018

**Methods**	
**Participants**	
**Interventions**	
**Outcomes**	
**Notes**	

Risk of bias table

Bias	Authors' judgement	Support for judgement
Random sequence generation (selection bias)	Low risk	“Randomization was stratified according to whether or not the women had received chemotherapy using a permuted block-randomization scheme with random-sized blocks of 6 or 8. A computerized randomization scheme was created by the Moores Cancer Center Biostatistics Shared Resource.” (p. 193)
Allocation concealment (selection bias)	Low risk	“Randomization was stratified according to whether or not the women had received chemotherapy using a permuted block-randomization scheme with random-sized blocks of 6 or 8. A computerized randomization scheme was created by the Moores Cancer Center Biostatistics Shared Resource.” (p. 193)
Blinding of participants and personnel (performance bias)	High risk	Due to the nature of the intervention and control conditions make blinding impossible.
Blinding of outcome assessment (detection bias)	Low risk	Blinding of outcomes assessment was not mentioned but the outcome measurement is not likely to be influenced by lack of blinding because outcomes were objectively measured.
Incomplete outcome data (attrition bias)	Low risk	“All analyses were performed using an intent-to-treat principal, with missing data assumed missing at random and accounted for in the longitudinal random-effects models by using a likelihood-based estimation method, which uses all available data and does not omit individuals with partially missing data.” (p. 194)
Selective reporting (reporting bias)	Low risk	The study protocol is available and all of the study’s pre-specified outcomes that are of interest in the review have been reported in the pre-specified way.
Other bias	Low risk	The study appears to be free of other sources of bias.

Hornikx, 2015

**Methods**	
**Participants**	
**Interventions**	
**Outcomes**	
**Notes**	

Risk of bias table

Bias	Authors' judgement	Support for judgement
Random sequence generation (selection bias)	Unclear risk	Insufficient information about the sequence generation process to permit judgement of ‘Low risk’ or ‘High risk’.
Allocation concealment (selection bias)	Unclear risk	Insufficient information to permit judgement of ‘Low risk’ or ‘High risk’.
Blinding of participants and personnel (performance bias)	High risk	Due to the nature of the intervention and control conditions make blinding impossible.
Blinding of outcome assessment (detection bias)	Low risk	Blinding of outcomes assessment was not mentioned but the outcome measurement is not likely to be influenced by lack of blinding because the primary outcome was objectively measured.
Incomplete outcome data (attrition bias)	Low risk	Plausible effect size (difference in means or standardized difference in means) among missing outcomes not enough to have a clinically relevant impact on observed effect size.
Selective reporting (reporting bias)	Low risk	The study protocol is not available but it is clear that the published reports include all expected outcomes, including those that were pre-specified
Other bias	Low risk	The study appears to be free of other sources of bias.

Jennings, 2016

**Methods**	
**Participants**	
**Interventions**	
**Outcomes**	
**Notes**	

Risk of bias table

Bias	Authors' judgement	Support for judgement
Random sequence generation (selection bias)	Low risk	“Randomization was done by a computer program.” (p. 350)
Allocation concealment (selection bias)	Unclear risk	Insufficient information to permit judgement of ‘Yes’ or ‘No’.
Blinding of participants and personnel (performance bias)	High risk	Due to the nature of the intervention and control conditions make blinding impossible.
Blinding of outcome assessment (detection bias)	Low risk	Blinding of outcomes assessment was not mentioned but the outcome measurement is not likely to be influenced by lack of blinding because outcomes were objectively measured.
Incomplete outcome data (attrition bias)	Unclear risk	Insufficient reporting of attrition/exclusions to permit judgement of ‘Yes’ or ‘No’.
Selective reporting (reporting bias)	Low risk	The study protocol is not available but it is clear that the published reports include all expected outcomes, including those that were pre-specified
Other bias	Low risk	The study appears to be free of other sources of bias.

Katz, 2018

**Methods**	
**Participants**	
**Interventions**	
**Outcomes**	
**Notes**	

Risk of bias table

Bias	Authors' judgement	Support for judgement
Random sequence generation (selection bias)	Low risk	“For randomization, sealed envelopes were prepared with group assignments in blocks of random size.” (p. 2)
Allocation concealment (selection bias)	Low risk	“Envelopes were numbered and opened sequentially at the time of the second visit.” (p. 2)
Blinding of participants and personnel (performance bias)	High risk	Due to the nature of the intervention and control conditions make blinding impossible.
Blinding of outcome assessment (detection bias)	Low risk	The outcome measurement is not likely to be influenced by lack of blinding because the primary outcome was objectively measured.
Incomplete outcome data (attrition bias)	Low risk	Plausible effect size (difference in means or standardized difference in means) among missing outcomes not enough to have a clinically relevant impact on observed effect size.

Selective reporting (reporting bias)	Low risk	The study protocol is available and all of the study's pre-specified (primary and secondary) outcomes that are of interest in the review have been reported in the pre-specified way.
Other bias	Low risk	The study appears to be free of other sources of bias.

Kooiman, 2018

**Methods**	
**Participants**	
**Interventions**	
**Outcomes**	
**Notes**	

Risk of bias table

**Bias**	**Authors' judgement**	**Support for judgement**
Random sequence generation (selection bias)	Unclear risk	Insufficient information about the sequence generation process to permit judgement of 'Yes' or 'No'. (block size were not sufficiently detailed).
Allocation concealment (selection bias)	Unclear risk	Insufficient information to permit judgement of 'Yes' or 'No'. (block size were not sufficiently detailed).
Blinding of participants and personnel (performance bias)	High risk	Due to the nature of the intervention and control conditions make blinding impossible.
Blinding of outcome assessment (detection bias)	Low risk	Blinding of outcomes assessment was not mentioned but the outcome measurement is not likely to be influenced by lack of blinding because primary outcomes were objectively measured.
Incomplete outcome data (attrition bias)	Low risk	"Within the intervention group, a mixed models analysis was used to analyze the change of physical activity over time, measured as average steps per day from week 0 (baseline) until week 12. The advantage of mixed models is that this method can handle missing data; for example, a participant with a missing week of average steps per day data would still be included in the analysis." (p. 344).
Selective reporting (reporting bias)	Low risk	The study protocol is available and all of the study's pre-specified (primary and secondary) outcomes that are of interest in the review have been reported in the pre-specified way.
Other bias	Low risk	The study appears to be free of other sources of bias.

Li, 2017

**Methods**	
**Participants**	
**Interventions**	
**Outcomes**	
**Notes**	

Risk of bias table

Bias	Authors' judgement	Support for judgement
Random sequence generation (selection bias)	Low risk	“Random numbers were generated in variable block sizes for the random allocation.” (p.3)
Allocation concealment (selection bias)	Low risk	“Random numbers were generated in variable block sizes for the random allocation.” (p.3)
Blinding of participants and personnel (performance bias)	High risk	Due to the nature of the intervention and control conditions make blinding impossible.
Blinding of outcome assessment (detection bias)	Low risk	Blinding of outcomes assessment was not mentioned but the outcome measurement is not likely to be influenced by lack of blinding because primary outcomes were objectively measured.
Incomplete outcome data (attrition bias)	Low risk	“We assessed the impact of missing data on the estimated effects of group assignment using imputation methods as described in van Buuren [45]. Specifically, we generated 10 imputed values using alternative random variates derived in a linear regression model, which included group, sex, baseline age, and baseline body mass index as predictors. We repeated the analyses using the 10 imputed values, and compared the conclusions and estimates against the main analysis, which assumed that data were missing at random.” (p. 5)
Selective reporting (reporting bias)	Low risk	The study protocol is available and all of the study’s pre-specified (primary and secondary) outcomes that are of interest in the review have been reported in the pre-specified way.
Other bias	Low risk	The study appears to be free of other sources of bias.

Li, 2018

**Methods**	
**Participants**	
**Interventions**	
**Outcomes**	

**Notes**	

Risk of bias table

Bias	Authors' judgement	Support for judgement
Random sequence generation (selection bias)	Low risk	“We performed randomization using computer-generated random numbers in variable block sizes”. (p. 3)
Allocation concealment (selection bias)	Low risk	“We performed randomization using computer-generated random numbers in variable block sizes”. (p. 3)
Blinding of participants and personnel (performance bias)	High risk	Due to the nature of the intervention and control conditions make blinding impossible.
Blinding of outcome assessment (detection bias)	Low risk	“An intention-to-treat analysis was performed by a biostatistician who was blinded to the group assignment.” (p. 4).
Incomplete outcome data (attrition bias)	Low risk	Plausible effect size (difference in means or standardized difference in means) among missing outcomes not enough to have a clinically relevant impact on observed effect size.
Selective reporting (reporting bias)	Low risk	The study protocol is available and all of the study’s pre-specified (primary and secondary) outcomes that are of interest in the review have been reported in the pre-specified way.
Other bias	Low risk	The study appears to be free of other sources of bias.

Lystrup, 2016

**Methods**	
**Participants**	
**Interventions**	
**Outcomes**	
**Notes**	

Risk of bias table

Bias	Authors' judgement	Support for judgement
Random sequence generation (selection bias)	Unclear risk	Insufficient information about the sequence generation process to permit judgement of ‘Yes’ or ‘No’.
Allocation concealment (selection bias)	Unclear risk	Insufficient information to permit judgement of ‘Yes’ or ‘No’.
Blinding of participants and personnel (performance bias)	High risk	Due to the nature of the intervention and control conditions make blinding impossible.
Blinding of outcome assessment (detection bias)	High risk	"This was a prospective, unblinded clinical trial of pedometry." (p. 1235)

Incomplete outcome data (attrition bias)	High risk	"For this study, we limited analysis to the 42 subjects in the control group and 44 subjects in the intervention group who completed running events." (p. 1236).
Selective reporting (reporting bias)	Low risk	The study protocol is not available but it is clear that the published reports include all expected outcomes, including those that were pre-specified.
Other bias	Low risk	The study appears to be free of other sources of bias.

Mahar, 2015

**Methods**	
**Participants**	
**Interventions**	
**Outcomes**	
**Notes**	

Risk of bias table

**Bias**	**Authors' judgement**	**Support for judgement**
Random sequence generation (selection bias)	Unclear risk	Insufficient information about the sequence generation process to permit judgement of ‘Yes’ or ‘No’.
Allocation concealment (selection bias)	Unclear risk	Insufficient information to permit judgement of ‘Yes’ or ‘No’.
Blinding of participants and personnel (performance bias)	High risk	Due to the nature of the intervention and control conditions make blinding impossible.
Blinding of outcome assessment (detection bias)	Low risk	Blinding of outcomes assessment was not mentioned but the outcome measurement is not likely to be influenced by lack of blinding because primary outcomes were objectively measured.
Incomplete outcome data (attrition bias)	Unclear risk	Insufficient reporting of attrition/exclusions to permit judgement of ‘Yes’ or ‘No’.
Selective reporting (reporting bias)	Low risk	The study protocol is not available but it is clear that the published reports include all expected outcomes, including those that were pre-specified
Other bias	Low risk	The study appears to be free of other sources of bias.

McDermott, 2018

**Methods**	
**Participants**	
**Interventions**	
**Outcomes**	

**Notes**	

Risk of bias table

Bias	Authors' judgement	Support for judgement
Random sequence generation (selection bias)	Low risk	“Participants were randomized to 1 of 2 groups using a computer-Generated randomization list. Randomization was stratified by study site and used block randomization and randomly selected block sizes of 4, 6, or 8.” (p. 1667)
Allocation concealment (selection bias)	Low risk	“Participants were randomized to 1 of 2 groups using a computer-Generated randomization list. Randomization was stratified by study site and used block randomization and randomly selected block sizes of 4, 6, or 8.” (p. 1667)
Blinding of participants and personnel (performance bias)	High risk	Due to the nature of the intervention and control conditions make blinding impossible.
Blinding of outcome assessment (detection bias)	Low risk	“All prespecified outcomes were obtained by staff blinded to group assignment at baseline and follow-up” (p. 1667)
Incomplete outcome data (attrition bias)	Low risk	“Analyses for changes in outcomes between baseline and 4½-month follow-up and between baseline and 9-month follow-up were performed using multiple imputation for missing data and SAS Proc MI (SAS Institute Inc) with 80 imputed data sets” (p. 1668)<br>In the footnote of table 3 (p. 1671), they reported further details.
Selective reporting (reporting bias)	Low risk	The study protocol is available and all of the study’s pre-specified outcomes that are of interest in the review have been reported in the pre-specified way.
Other bias	Low risk	The study appears to be free of other sources of bias.

Mendoza, 2017

**Methods**	
**Participants**	
**Interventions**	
**Outcomes**	
**Notes**	

Risk of bias table

Bias	Authors' judgement	Support for judgement
Random sequence generation (selection bias)	Unclear risk	Insufficient information about the sequence generation process to permit judgement of ‘Yes’ or ‘No’.

Allocation concealment (selection bias)	Unclear risk	Insufficient information to permit judgement of ‘Yes’ or ‘No’.
Blinding of participants and personnel (performance bias)	High risk	Due to the nature of the intervention and control conditions make blinding impossible.
Blinding of outcome assessment (detection bias)	Low risk	"We conducted a two-arm, unblinded, RCT [...]" (p. 2/9).
Incomplete outcome data (attrition bias)	Low risk	No missing outcome data.
Selective reporting (reporting bias)	Low risk	The study protocol is available and all of the study’s pre-specified (primary and secondary) outcomes that are of interest in the review have been reported in the pre-specified way.
Other bias	Low risk	The study appears to be free of other sources of bias.

Miragall, 2018

**Methods**	
**Participants**	
**Interventions**	
**Outcomes**	
**Notes**	

Risk of bias table

**Bias**	**Authors' judgement**	**Support for judgement**
Random sequence generation (selection bias)	Unclear risk	Insufficient information about the sequence generation process to permit judgement of ‘Low risk’ or ‘High risk’.
Allocation concealment (selection bias)	Unclear risk	Insufficient information to permit judgement of ‘Low risk’ or ‘High risk’
Blinding of participants and personnel (performance bias)	High risk	Due to the nature of the intervention and control conditions make blinding impossible.
Blinding of outcome assessment (detection bias)	Low risk	Blinding of outcomes assessment was not mentioned but the outcome measurement is not likely to be influenced by lack of blinding because primary outcomes were objectively measured.
Incomplete outcome data (attrition bias)	Low risk	Plausible effect size among missing outcomes not enough to have a clinically relevant impact on observed effect size.
Selective reporting (reporting bias)	Low risk	The study protocol is available (NCT02838550) and all of the study’s pre-specified (primary and secondary) outcomes that are of interest in the review have been reported in the pre-specified way.
Other bias	Low risk	The study appears to be free of other sources of bias.

Oliveira, 2019

**Methods**	
**Participants**	
**Interventions**	
**Outcomes**	
**Notes**	

Risk of bias table

Bias	Authors' judgement	Support for judgement
Random sequence generation (selection bias)	Low risk	“Participants were randomly assigned (1:1) to either the intervention or control group, by an investigator not involved in recruitment (CS), using a computer-generated random number schedule with randomly permuted block sizes of two and six”. (p. 17)
Allocation concealment (selection bias)	Low risk	“Participants were randomly assigned (1:1) to either the intervention or control group, by an investigator not involved in recruitment (CS), using a computer-generated random number schedule with randomly permuted block sizes of two and six”. (p. 17)
Blinding of participants and personnel (performance bias)	High risk	Due to the nature of the intervention and control conditions make blinding impossible.
Blinding of outcome assessment (detection bias)	Low risk	“Data from the primary outcomes (mobility-related goal attainment and objectively measured physical activity) and secondary outcomes were collected by a research assistant who was blinded to group assignment”. (p. 17)
Incomplete outcome data (attrition bias)	High risk	High attrition (more than 25%).<br>Plausible effect size among missing outcomes enough to induce clinically relevant bias in observed effect size.
Selective reporting (reporting bias)	Low risk	The study protocol is available and all of the study’s pre-specified (primary and secondary) outcomes that are of interest in the review have been reported in the pre-specified way.
Other bias	Low risk	The study appears to be free of other sources of bias.

Paxton, 2018

**Methods**	
**Participants**	
**Interventions**	
**Outcomes**	
**Notes**	

Risk of bias table

Bias	Authors' judgement	Support for judgement
Random sequence generation (selection bias)	Unclear risk	Insufficient information about the sequence generation process to permit judgement of 'Yes' or 'No'.
Allocation concealment (selection bias)	Unclear risk	Insufficient information to permit judgement of 'Low risk' or 'High risk'.
Blinding of participants and personnel (performance bias)	High risk	Due to the nature of the intervention and control conditions make blinding impossible.
Blinding of outcome assessment (detection bias)	Low risk	Blinding of outcomes assessment was not mentioned but the outcome measurement is not likely to be influenced by lack of blinding because outcomes were objectively measured.
Incomplete outcome data (attrition bias)	Low risk	Missing outcome data balanced in numbers across intervention groups, with similar reasons for missing data across groups.
Selective reporting (reporting bias)	Low risk	The study protocol is available and all of the study's pre-specified (primary and secondary) outcomes that are of interest in the review have been reported in the pre-specified way.
Other bias	Low risk	The study appears to be free of other sources of bias.

Redman, 2017

**Methods**	
**Participants**	
**Interventions**	
**Outcomes**	
**Notes**	

Risk of bias table

Bias	Authors' judgement	Support for judgement
Random sequence generation (selection bias)	Low risk	"The block randomization schedule and sealed numbered randomization envelopes were prepared by the biostatistician" (p. 2)
Allocation concealment (selection bias)	Low risk	"The block randomization schedule and sealed numbered randomization envelopes were prepared by the biostatistician" (p. 2)
Blinding of participants and personnel (performance bias)	High risk	Due to the nature of the intervention and control conditions make blinding impossible.
Blinding of outcome assessment (detection bias)	Low risk	"Clinic assessments were performed by certified staff who were blinded to group assignment". (p. 2)

Incomplete outcome data (attrition bias)	Unclear risk	Insufficient reporting of attrition/exclusions to permit judgement of ‘Yes’ or ‘No’.
Selective reporting (reporting bias)	Low risk	The study protocol is available and all of the study’s pre-specified (primary and secondary) outcomes that are of interest in the review have been reported in the pre-specified way.
Other bias	Low risk	The study appears to be free of other sources of bias.

Shoemaker, 2016

**Methods**	
**Participants**	
**Interventions**	
**Outcomes**	
**Notes**	

Risk of bias table

**Bias**	**Authors' judgement**	**Support for judgement**
Random sequence generation (selection bias)	Low risk	“Subjects were randomized with a random number generator […]” (p. 112)
Allocation concealment (selection bias)	Unclear risk	Insufficient information to permit judgement of "Low risk" or "High risk".
Blinding of participants and personnel (performance bias)	High risk	Due to the nature of the intervention and control conditions make blinding impossible.
Blinding of outcome assessment (detection bias)	Low risk	“Members of the investigation team who were blinded to group assignment conducted the follow-up measurements” (p. 112)
Incomplete outcome data (attrition bias)	Low risk	Plausible effect size among missing outcomes not enough to have a clinically relevant impact on observed effect size.
Selective reporting (reporting bias)	Low risk	The study protocol is available and all of the study’s pre-specified (primary and secondary) outcomes that are of interest in the review have been reported in the pre-specified way.
Other bias	Low risk	The study appears to be free of other sources of bias.

Simons, 2018

**Methods**	
**Participants**	
**Interventions**	
**Outcomes**	

**Notes**	

Risk of bias table

Bias	Authors' judgement	Support for judgement
Random sequence generation (selection bias)	Low risk	“Allocation was based on clusters (workplaces), which were randomly assigned following block randomization (restricted randomization) to the intervention or the control group.Block sizes varied randomly (2, 4, or 6), and for each block of clusters, half (1, 2,or 3) would be allocated to each arm of the study (intervention or control group)” (p. 3).
Allocation concealment (selection bias)	Low risk	“Allocation was based on clusters (workplaces), which were randomly assigned following block randomization (restricted randomization) to the intervention or the control group.Block sizes varied randomly (2, 4, or 6), and for each block of clusters, half (1, 2,or 3) would be allocated to each arm of the study (intervention or control group)” (p. 3).
Blinding of participants and personnel (performance bias)	High risk	Due to the nature of the intervention and control conditions make blinding impossible.
Blinding of outcome assessment (detection bias)	Low risk	Blinding of outcomes assessment was not mentioned but the outcome measurement is not likely to be influenced by lack of blinding because outcomes were objectively measured.
Incomplete outcome data (attrition bias)	Low risk	Missing outcome data balanced in numbers across intervention groups, with similar reasons for missing data across groups.
Selective reporting (reporting bias)	Low risk	The study protocol is available and all of the study’s pre-specified (primary and secondary) outcomes that are of interest in the review have been reported in the pre-specified way.
Other bias	Low risk	The study appears to be free of other sources of bias.

Thompson, 2014

**Methods**	
**Participants**	
**Interventions**	
**Outcomes**	
**Notes**	

Risk of bias table

Bias	Authors' judgement	Support for judgement
Random sequence generation (selection bias)	Low risk	“Randomization was done using a table of random numbers and sealed opaque envelopes”. (p. 315)
Allocation concealment (selection bias)	Low risk	“Randomization was done using a table of random numbers and sealed opaque envelopes”. (p. 315)
Blinding of participants and personnel (performance bias)	High risk	Due to the nature of the intervention and control conditions make blinding impossible.
Blinding of outcome assessment (detection bias)	Low risk	Blinding of outcomes assessment was not mentioned but the outcome measurement is not likely to be influenced by lack of blinding because outcomes were objectively measured.
Incomplete outcome data (attrition bias)	Low risk	Missing outcome data balanced in numbers across intervention groups, with similar reasons for missing data across groups.
Selective reporting (reporting bias)	Low risk	The study protocol is available and all of the study’s pre-specified (primary and secondary) outcomes that are of interest in the review have been reported in the pre-specified way.
Other bias	Low risk	The study appears to be free of other sources of bias.

Thorndike, 2014

**Methods**	
**Participants**	
**Interventions**	
**Outcomes**	
**Notes**	

Risk of bias table

Bias	Authors' judgement	Support for judgement
Random sequence generation (selection bias)	Low risk	“Prior to Phase 1, all participants were simultaneously randomized to the intervention or control arm, stratifying by year of training. A random number was assigned to each subject using the built-in random number function in Microsoft Access”. (p. 3)
Allocation concealment (selection bias)	Low risk	"Prior to Phase 1, all participants were simultaneously randomized to the intervention or control arm, stratifying by year of training. A random number was assigned to each subject using the built-in random number function in Microsoft Access. A list was created in which subjects were grouped by year of training and then sorted by the random number within each year.The study statistician generated the random allocation sequence." (p.3)

Blinding of participants and personnel (performance bias)	High risk	Due to the nature of the intervention and control conditions make blinding impossible.
Blinding of outcome assessment (detection bias)	Low risk	Blinding of outcomes assessment was not mentioned but the outcome measurement is not likely to be influenced by lack of blinding because outcomes were objectively measured.
Incomplete outcome data (attrition bias)	Low risk	Missing outcome data balanced in numbers across intervention groups, with similar reasons for missing data across groups.
Selective reporting (reporting bias)	Low risk	The study protocol is available and all of the study's pre-specified (primary and secondary) outcomes that are of interest in the review have been reported in the pre-specified way.
Other bias	Low risk	The study appears to be free of other sources of bias.

Van Blarigan, 2019

**Methods**	
**Participants**	
**Interventions**	
**Outcomes**	
**Notes**	

Risk of bias table

**Bias**	**Authors' judgement**	**Support for judgement**
Random sequence generation (selection bias)	Low risk	"Participants were randomized 1:1 to intervention or control using a computer-generated randomization scheme developed by a study biostatistician (LZ) who was blinded to group assignment" (p. 2/9)
Allocation concealment (selection bias)	Low risk	"This scheme was uploaded to REDCap, and a clinical research coordinator utilized the randomization tool in REDCap to obtain the group assignment for each participant." (p. 2)
Blinding of participants and personnel (performance bias)	High risk	Due to the nature of the intervention and control conditions make blinding impossible.
Blinding of outcome assessment (detection bias)	Low risk	Blinding of outcomes assessment was not mentioned but the outcome measurement is not likely to be influenced by lack of blinding because outcomes were objectively measured.
Incomplete outcome data (attrition bias)	Low risk	"Follow-up (based on completion and return of the accelerometer at 12-weeks) was 95% complete in the intervention arm and 90% complete in the control arm. The two patients who did not complete the 12-week accelerometer assessment in the control arm withdrew from the study (one due to cancer recurrence and the other due to obtaining her

		own Fitbit during the study period)" (p. 5/9)
Selective reporting (reporting bias)	Low risk	The study protocol is available and all of the study's pre-specified (primary and secondary) outcomes that are of interest in the review have been reported in the pre-specified way.
Other bias	Low risk	The study appears to be free of other sources of bias.

Vandelanotte, 2018

**Methods**	
**Participants**	
**Interventions**	
**Outcomes**	
**Notes**	

Risk of bias table

**Bias**	**Authors' judgement**	**Support for judgement**
Random sequence generation (selection bias)	Low risk	"[…] participants were randomized into 1 of the 2 groups in a ratio of 1:1 using a random list generator […]" (pp. 2-3)
Allocation concealment (selection bias)	Low risk	Allocation was done autormatically by a web-based system. "After completing the Web-based screening tool, eligible participants completed Web-based baseline surveys (see Measures section below). After completing baseline assessments, participants were randomized into 1 of the 2 groups in a ratio of 1:1 using a random list generator and provided with access to the TaylorActive intervention (see Intervention section below)." (pp. 2-3)
Blinding of participants and personnel (performance bias)	High risk	Due to the nature of the intervention and control conditions make blinding impossible.
Blinding of outcome assessment (detection bias)	High risk	No blinding or incomplete blinding, and the outcome or outcome measurement is likely to be influenced by lack of blinding.
Incomplete outcome data (attrition bias)	Low risk	"In total, 3 more separate linear mixed model analyses were conducted to test a group by time interaction effects on moderate-to-vigorous physical activity, sitting time, and BMI. All linear mixed model analyses applied restricted maximum likelihood estimation to reduce risk of bias from missing data" (p. 4)
Selective reporting (reporting bias)	Low risk	The study protocol is available and all of the study's pre-specified (primary and secondary) outcomes that are of interest in the review have been reported in the pre-specified way.
Other bias	Low risk	The study appears to be free of other sources of bias.

Footnotes

Summary of findings tables

Additional tables
