# Supplementary material for: Fitbit-Based Interventions for Healthy Lifestyle Outcomes: Systematic Review and Meta-Analysis
Source: J Med Internet Res. 2020 Oct 12;22(10):e23954. doi: 10.2196/23954 (PMC7589007; doi:10.2196/23954)

1. Steps
   1. Theory-based analysis


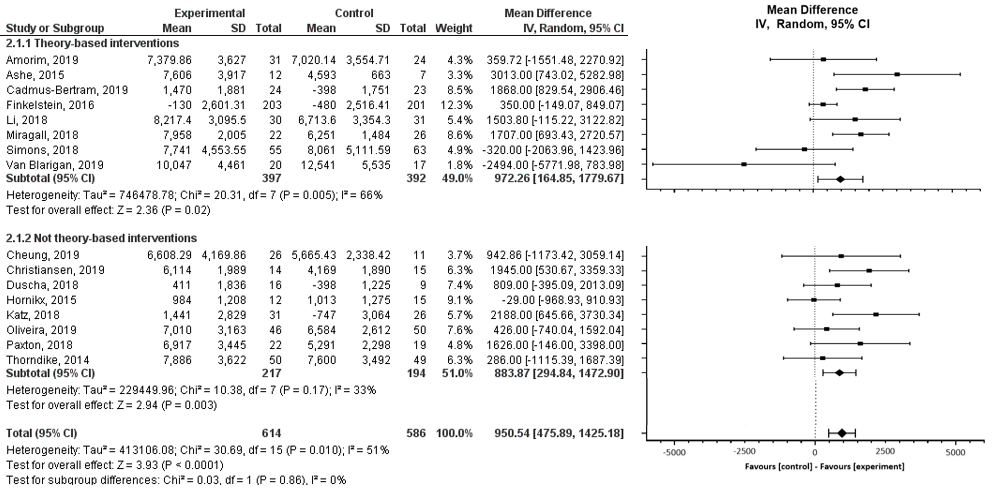


- 1. Condition-based analysis


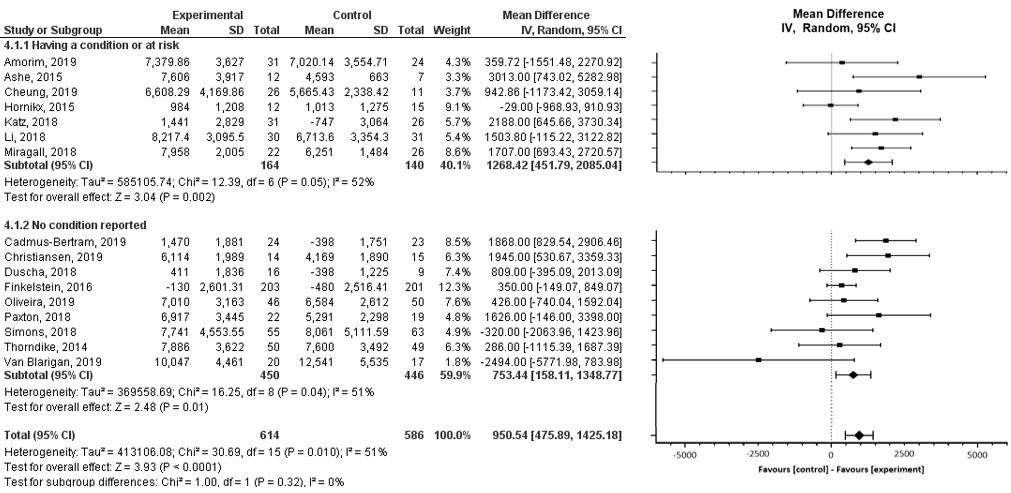


- 1. Length-based analysis


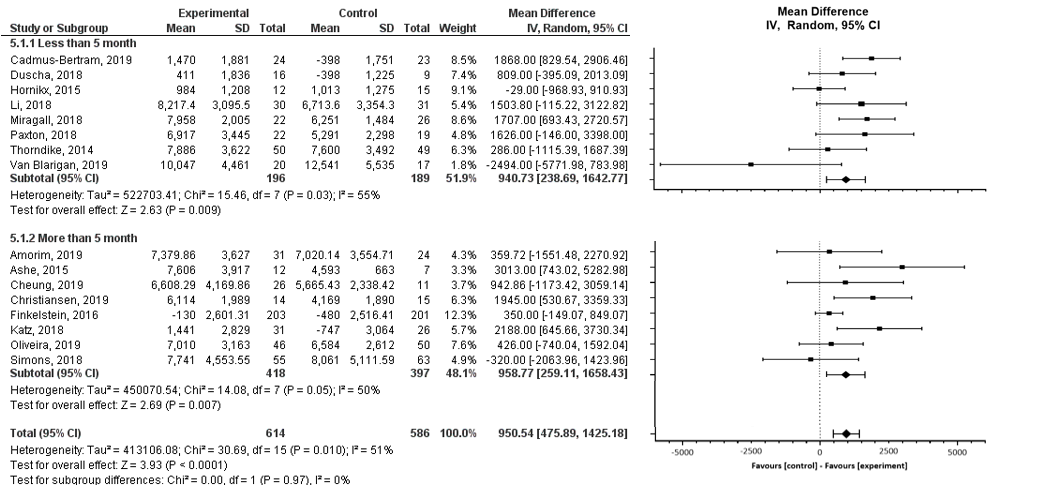


- 1. Post-intervention data vs mean change data


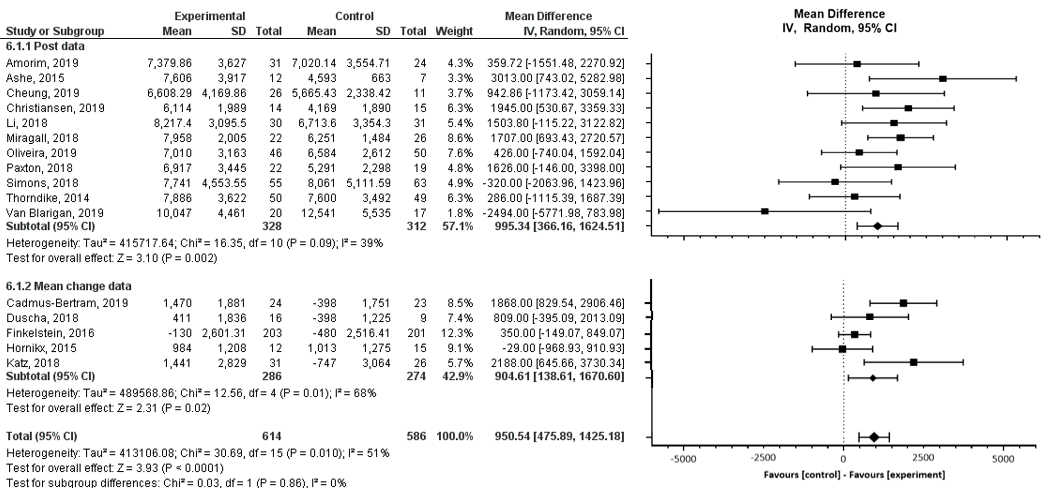


1. MVPA
   1. Theory-based analysis


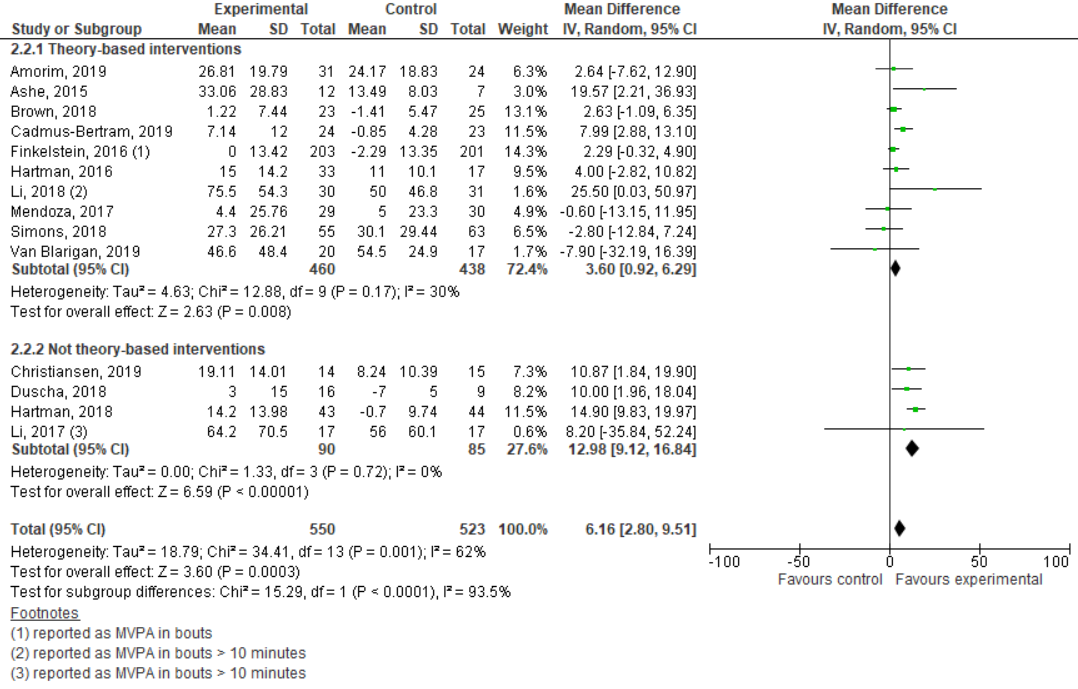


- 1. Condition-based analysis


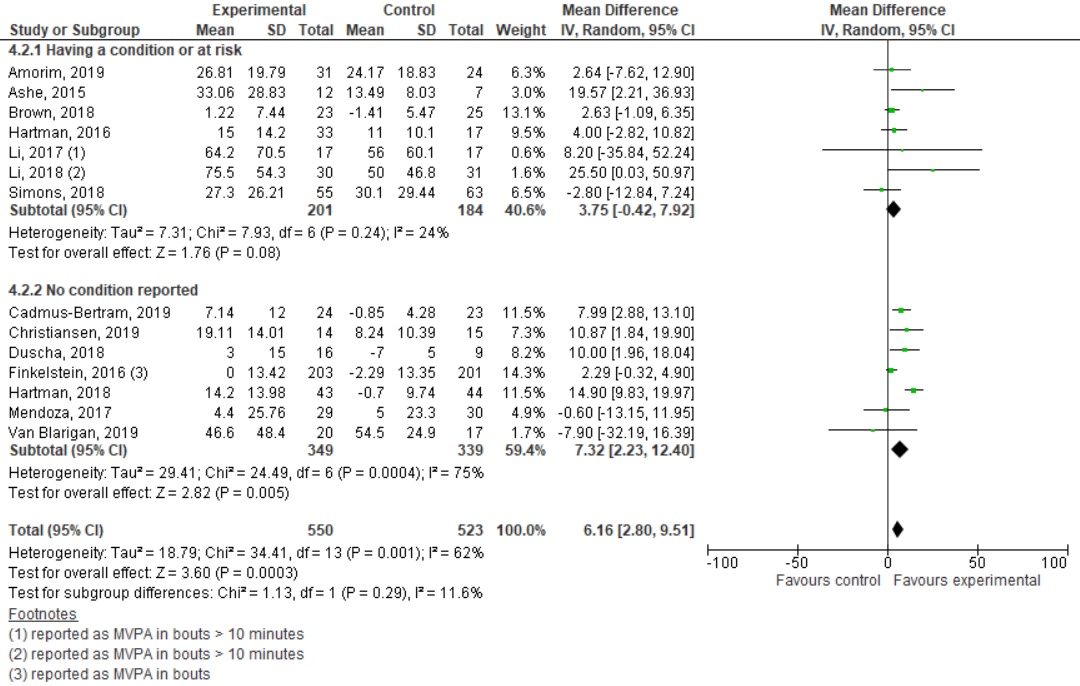


- 1. Length-based analysis


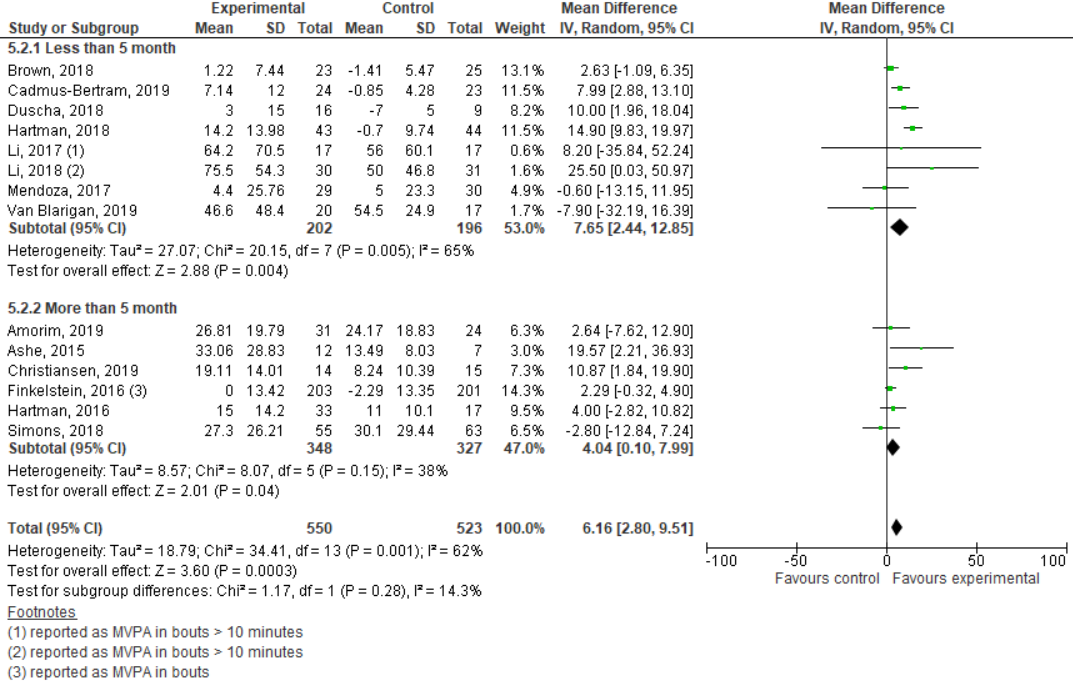


- 1. Post-intervention data vs mean change data


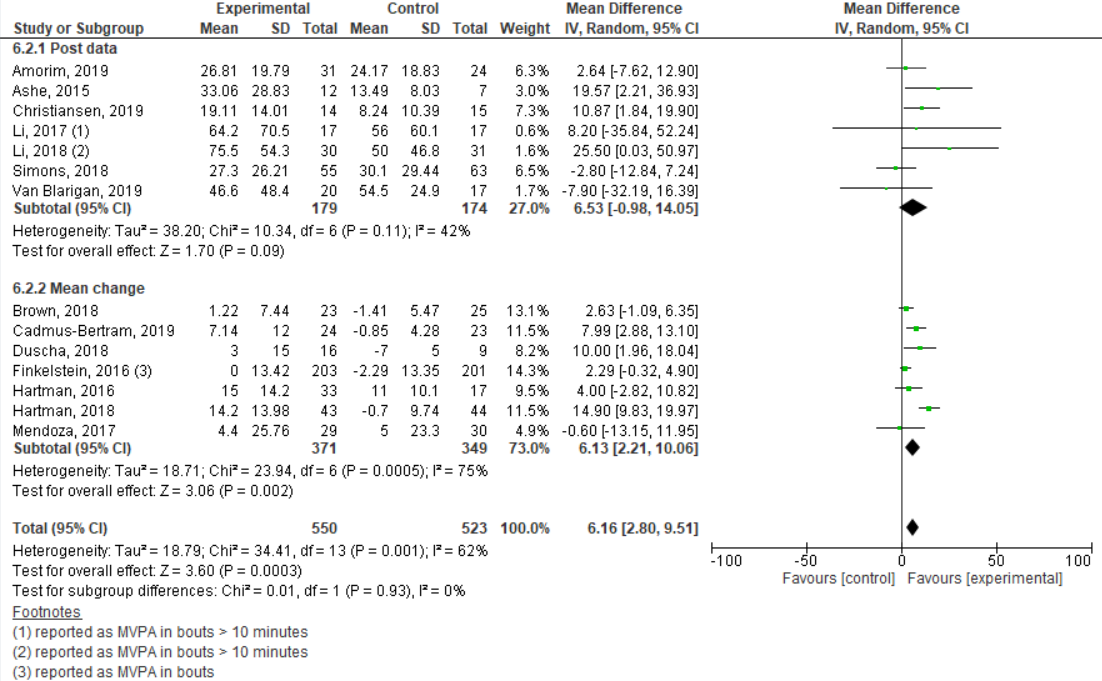


1. Weight
   1. Theory-based analysis


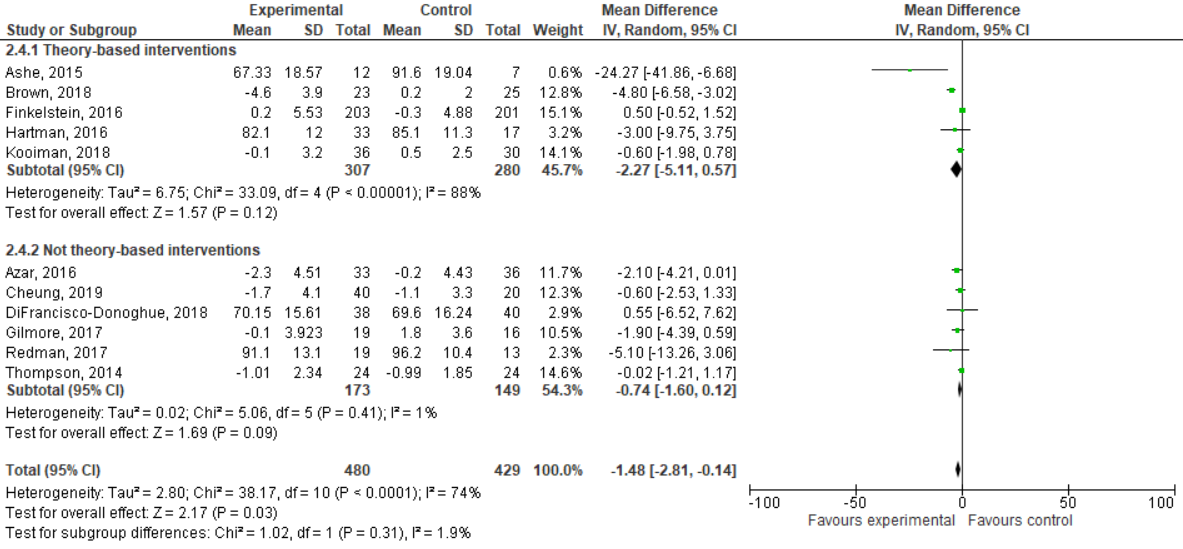


- 1. Condition-based analysis


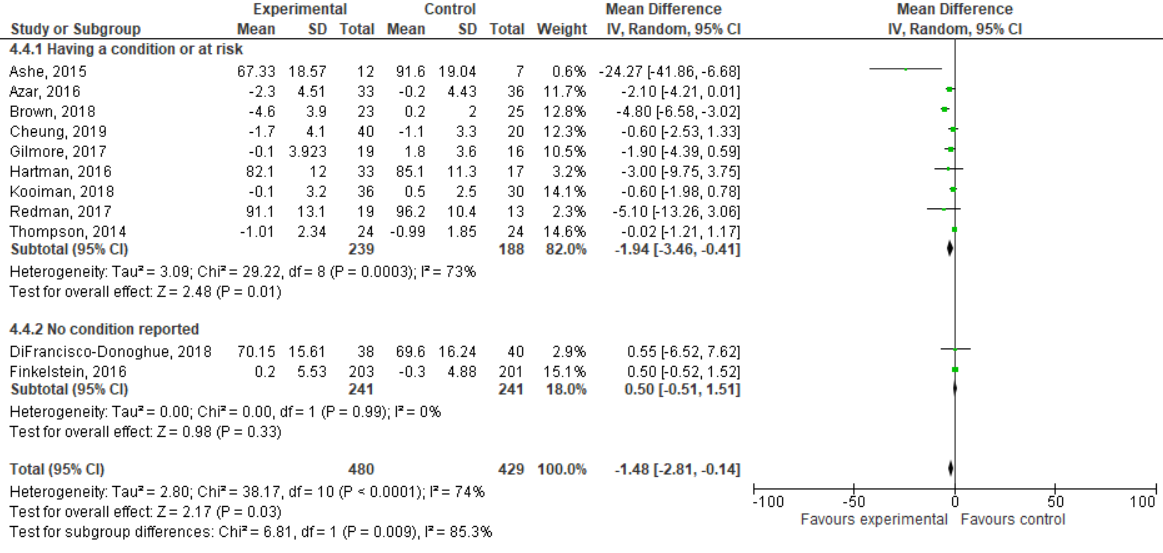


- 1. Length-based analysis


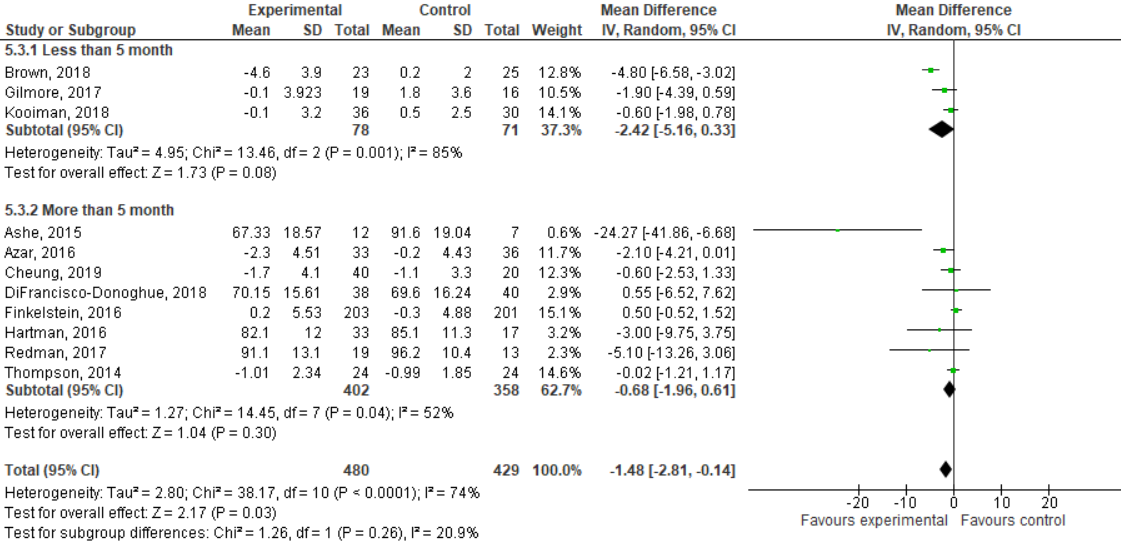


- 1. Post-intervention data vs mean change data


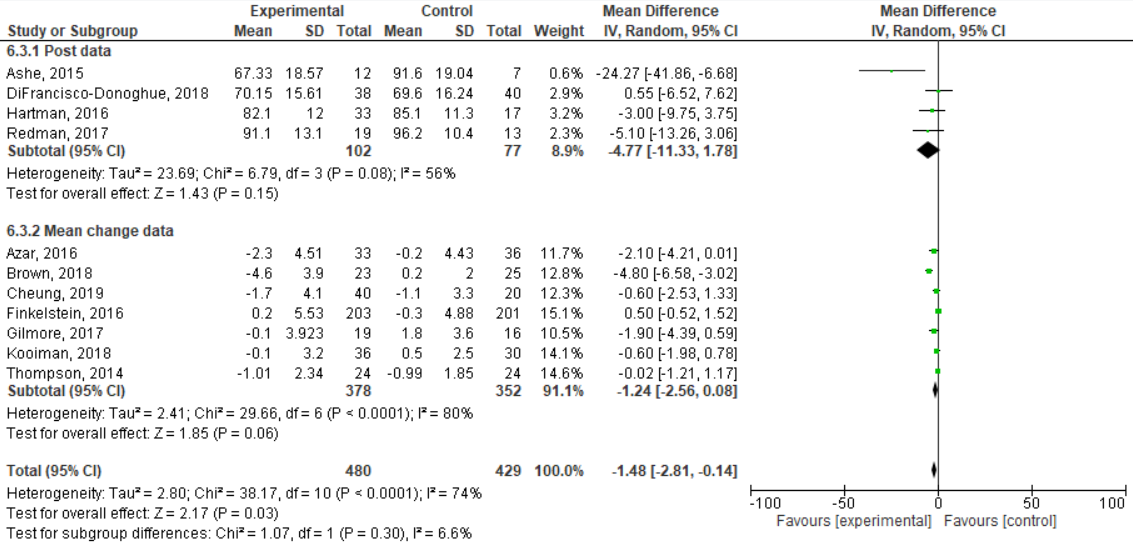

Supplement: Multimedia Appendix 8 [file jmir_v22i10e23954_app8.docx]
