# Supplementary material for: Fitbit-Based Interventions for Healthy Lifestyle Outcomes: Systematic Review and Meta-Analysis
Source: J Med Internet Res. 2020 Oct 12;22(10):e23954. doi: 10.2196/23954 (PMC7589007; doi:10.2196/23954)

1. Steps


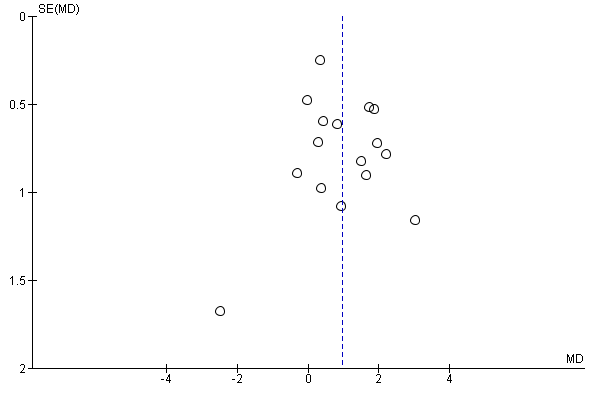


NB: We reduced the scale of the measure by dividing means and SD by 1,000 so we can draw the funnel plot with Review Manager.

1. MVPA


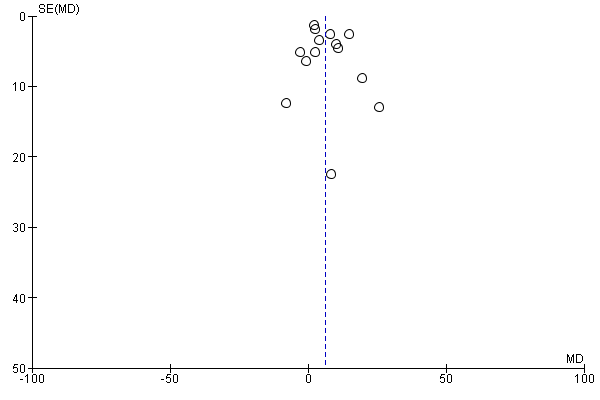


1. Weight


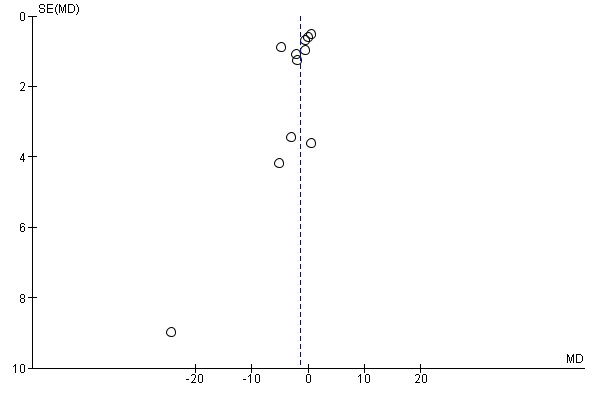

Supplement: Multimedia Appendix 9 [file jmir_v22i10e23954_app9.docx]
