# Supplementary material for: Fitbit-Based Interventions for Healthy Lifestyle Outcomes: Systematic Review and Meta-Analysis
Source: J Med Internet Res. 2020 Oct 12;22(10):e23954. doi: 10.2196/23954 (PMC7589007; doi:10.2196/23954)
Supplement: Multimedia Appendix 10 [file jmir_v22i10e23954_app10.docx]

Table 1. Truth table for intervention components only – outcome set positively

| **Goal-setting** | **Messaging** | **Education** | **Counseling** | **Social** | **Self-monitoring** | **Observations** | **Outcome** | **Raw consist.** | **PRI consist.** |
| --- | --- | --- | --- | --- | --- | --- | --- | --- | --- |
| 1 | 1 | 1 | 1 | 0 | 1 | 2 | 1 | 0.970 | 0.969 |
| 1 | 0 | 1 | 1 | 1 | 0 | 5 | 1 | 0.948 | 0.945 |
| 1 | 0 | 1 | 1 | 0 | 1 | 4 | 1 | 0.918 | 0.910 |
| 1 | 0 | 1 | 0 | 1 | 0 | 8 | 1 | 0.841 | 0.825 |
| 1 | 0 | 0 | 1 | 0 | 0 | 5 | 0 | 0.642 | 0.626 |
| 1 | 0 | 0 | 1 | 0 | 1 | 4 | 0 | 0.618 | 0.381 |
| 0 | 0 | 1 | 1 | 1 | 1 | 3 | 0 | 0.550 | 0.384 |
| 1 | 1 | 0 | 1 | 0 | 0 | 10 | 0 | 0.393 | 0.299 |
| 1 | 0 | 1 | 1 | 0 | 0 | 5 | 0 | 0.392 | 0.174 |
| 1 | 1 | 1 | 0 | 0 | 0 | 5 | 0 | 0.378 | 0.175 |
| 1 | 1 | 0 | 0 | 0 | 0 | 5 | 0 | 0.372 | 0.043 |
| 0 | 0 | 0 | 1 | 0 | 0 | 2 | 0 | 0.355 | 0.199 |
| 0 | 0 | 1 | 1 | 0 | 0 | 10 | 0 | 0.341 | 0.245 |
| 0 | 0 | 0 | 0 | 0 | 1 | 2 | 0 | 0.310 | 0.148 |
| 0 | 0 | 0 | 0 | 0 | 0 | 8 | 0 | 0.300 | 0.263 |
| 0 | 0 | 1 | 0 | 0 | 0 | 10 | 0 | 0.269 | 0.054 |
| 1 | 1 | 1 | 1 | 0 | 0 | 4 | 0 | 0.173 | 0.000 |
| 1 | 1 | 0 | 0 | 1 | 0 | 2 | 0 | 0.100 | 0.000 |
| 0 | 1 | 1 | 0 | 0 | 0 | 4 | 0 | 0.025 | 0.000 |

Table 2. Truth table for study and individual characteristics only – outcome set positively

| **Theory** | **Condition** | **Follow-up duration** | **Observations** | **Outcome** | **Raw consist.** | **PRI consist.** |
| --- | --- | --- | --- | --- | --- | --- |
| 1 | 1 | 1 | 12 | 0 | 0.716 | 0.518 |
| 1 | 1 | 0 | 24 | 0 | 0.712 | 0.612 |
| 0 | 1 | 1 | 11 | 0 | 0.630 | 0.454 |
| 0 | 0 | 0 | 7 | 0 | 0.605 | 0.443 |
| 1 | 0 | 0 | 15 | 0 | 0.540 | 0.502 |
| 0 | 1 | 0 | 8 | 0 | 0.510 | 0.355 |
| 0 | 0 | 1 | 25 | 0 | 0.416 | 0.182 |

No configuration has adequate consistency.

Table 3. Truth table for the main configuration – outcome set positively

| **Goal-setting** | **Messaging** | **Counseling** | **Theory** | **Condition** | **Follow-up duration** | **Observations** | **Outcome** | **Raw consist.** | **PRI consist.** |
| --- | --- | --- | --- | --- | --- | --- | --- | --- | --- |
| 1 | 0 | 1 | 1 | 0 | 0 | 5 | 1 | 0.997 | 0.997 |
| 1 | 0 | 1 | 1 | 1 | 1 | 3 | 1 | 0.979 | 0.945 |
| 1 | 1 | 1 | 0 | 1 | 0 | 2 | 1 | 0.960 | 0.937 |
| 1 | 0 | 0 | 1 | 1 | 0 | 4 | 1 | 0.950 | 0.943 |
| 1 | 1 | 1 | 0 | 1 | 1 | 2 | 1 | 0.933 | 0.830 |
| 1 | 0 | 0 | 1 | 1 | 1 | 5 | 1 | 0.924 | 0.905 |
| 0 | 0 | 1 | 1 | 1 | 0 | 4 | 1 | 0.904 | 0.866 |
| 1 | 0 | 1 | 1 | 1 | 0 | 7 | 1 | 0.872 | 0.808 |
| 1 | 1 | 1 | 0 | 0 | 0 | 4 | 0 | 0.750 | 0.727 |
| 0 | 0 | 0 | 1 | 0 | 0 | 4 | 0 | 0.658 | 0.621 |
| 1 | 0 | 1 | 0 | 0 | 1 | 6 | 0 | 0.542 | 0.450 |
| 1 | 1 | 0 | 1 | 1 | 0 | 5 | 0 | 0.493 | 0.236 |
| 1 | 1 | 0 | 0 | 0 | 1 | 5 | 0 | 0.448 | 0.058 |
| 0 | 0 | 1 | 0 | 1 | 1 | 8 | 0 | 0.430 | 0.215 |
| 0 | 0 | 0 | 0 | 0 | 0 | 2 | 0 | 0.411 | 0.048 |
| 0 | 0 | 1 | 0 | 1 | 0 | 3 | 0 | 0.299 | 0.087 |
| 0 | 0 | 0 | 0 | 0 | 1 | 14 | 0 | 0.291 | 0.032 |
| 1 | 1 | 1 | 1 | 1 | 1 | 4 | 0 | 0.245 | 0.000 |
| 1 | 1 | 1 | 1 | 1 | 0 | 4 | 0 | 0.217 | 0.000 |
| 1 | 1 | 0 | 1 | 0 | 0 | 2 | 0 | 0.127 | 0.000 |
| 0 | 1 | 0 | 1 | 0 | 0 | 4 | 0 | 0.033 | 0.000 |
