# Supplementary material for: Fitbit-Based Interventions for Healthy Lifestyle Outcomes: Systematic Review and Meta-Analysis
Source: J Med Internet Res. 2020 Oct 12;22(10):e23954. doi: 10.2196/23954 (PMC7589007; doi:10.2196/23954)
Supplement: Multimedia Appendix 11 [file jmir_v22i10e23954_app11.docx]

Figure 1. Configuration chart for intervention components only – outcome set positively


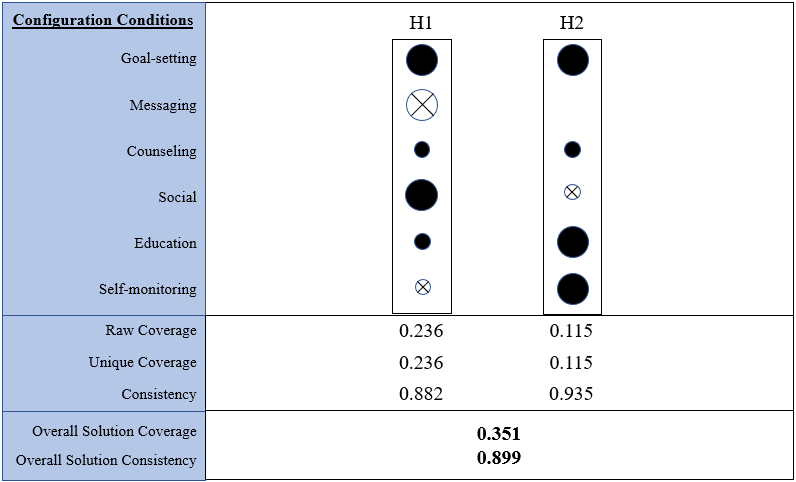


H1: Interventions that included goal setting, counseling, social, education, but without messaging and self-monitoring lead to better outcomes.

H2: Interventions that included goal setting, counseling, education, self-monitoring but without social lead to better outcomes.

Configuration chart for study and individual characteristics only could not be drawn because raw consistency and PRI consistency were too low.

Figure 2. Configuration chart for the main configuration – outcome set positively


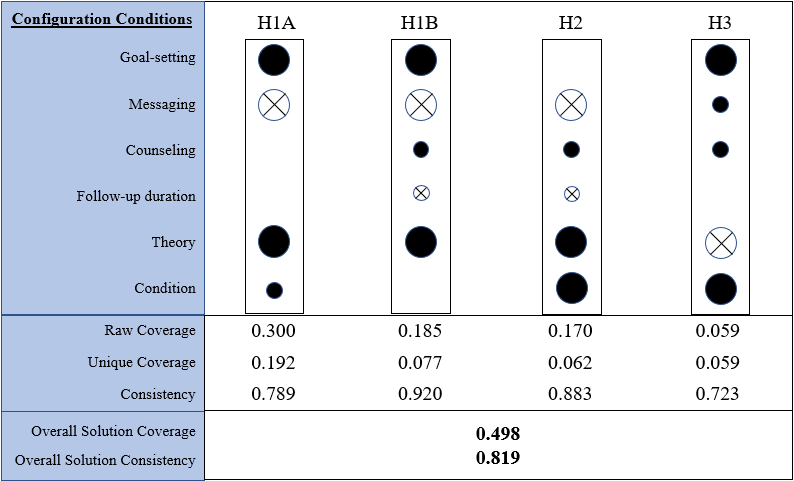


H1A: Theory-based interventions that included goal setting, but without messaging lead to better outcomes for individuals with pre-existing condition.

H1B: Theory-based interventions that included goal setting and counseling, but without messaging lead to better outcomes in short-term studies.

H2: Theory-based interventions that included counseling, but without messaging lead to better outcomes for individuals with pre-existing condition in short-term studies.

H3: Non-theory-based interventions that included goal setting, messaging and counseling lead to better outcomes for individuals with pre-existing condition.
