# Supplementary material for: Fitbit-Based Interventions for Healthy Lifestyle Outcomes: Systematic Review and Meta-Analysis
Source: J Med Internet Res. 2020 Oct 12;22(10):e23954. doi: 10.2196/23954 (PMC7589007; doi:10.2196/23954)
Supplement: Multimedia Appendix 12 [file jmir_v22i10e23954_app12.docx]

1. PA outcomes

Table 1. Truth table for the main configuration – outcome set positively

| **Messaging** | **Education** | **Counseling** | **Self-monitoring** | **Condition** | **Theory** | **Observations** | **Outcome** | **Raw consist.** | **PRI consist.** |
| --- | --- | --- | --- | --- | --- | --- | --- | --- | --- |
| 0 | 0 | 0 | 0 | 0 | 1 | 2 | 1 | 1.000 | 1.000 |
| 0 | 1 | 0 | 1 | 1 | 0 | 2 | 1 | 0.975 | 0.974 |
| 0 | 1 | 1 | 0 | 0 | 1 | 5 | 1 | 0.948 | 0.945 |
| 1 | 0 | 1 | 0 | 0 | 0 | 3 | 1 | 0.917 | 0.909 |
| 0 | 1 | 0 | 0 | 1 | 1 | 8 | 1 | 0.841 | 0.825 |
| 0 | 0 | 1 | 0 | 0 | 0 | 4 | 1 | 0.803 | 0.792 |
| 0 | 0 | 1 | 1 | 1 | 1 | 2 | 0 | 0.760 | 0.507 |
| 0 | 1 | 1 | 0 | 1 | 1 | 6 | 0 | 0.732 | 0.633 |
| 0 | 0 | 0 | 0 | 0 | 0 | 2 | 0 | 0.595 | 0.417 |
| 0 | 0 | 0 | 1 | 0 | 1 | 2 | 0 | 0.310 | 0.148 |
| 0 | 1 | 0 | 0 | 0 | 0 | 9 | 0 | 0.299 | 0.062 |
| 0 | 0 | 1 | 0 | 1 | 0 | 3 | 0 | 0.237 | 0.123 |
| 0 | 1 | 1 | 0 | 0 | 0 | 2 | 0 | 0.230 | 0.000 |
| 1 | 1 | 1 | 0 | 1 | 1 | 4 | 0 | 0.173 | 0.000 |
| 0 | 1 | 1 | 0 | 1 | 0 | 3 | 0 | 0.107 | 0.000 |
| 1 | 0 | 0 | 0 | 0 | 1 | 2 | 0 | 0.100 | 0.000 |
| 1 | 0 | 1 | 0 | 1 | 1 | 4 | 0 | 0.058 | 0.000 |
| 1 | 1 | 0 | 0 | 0 | 1 | 4 | 0 | 0.025 | 0.000 |

Table 2. Truth table for the configuration presented in the result section– outcome set positively

| **Goal-setting** | **Messaging** | **Counseling** | **Theory** | **Condition** | **Follow-up duration** | **Observations** | **Outcome** | **Raw consist.** | **PRI consist.** |
| --- | --- | --- | --- | --- | --- | --- | --- | --- | --- |
| 1 | 1 | 1 | 0 | 0 | 0 | 3 | 1 | 1.000 | 1.000 |
| 1 | 0 | 1 | 1 | 1 | 1 | 2 | 1 | 1.000 | 1.000 |
| 1 | 0 | 1 | 1 | 0 | 0 | 5 | 1 | 0.997 | 0.997 |
| 1 | 0 | 0 | 1 | 1 | 0 | 4 | 1 | 0.945 | 0.936 |
| 1 | 0 | 1 | 1 | 1 | 0 | 3 | 1 | 0.926 | 0.848 |
| 1 | 0 | 0 | 1 | 1 | 1 | 4 | 1 | 0.912 | 0.884 |
| 0 | 0 | 1 | 1 | 1 | 0 | 4 | 1 | 0.904 | 0.866 |
| 0 | 0 | 0 | 1 | 0 | 0 | 4 | 0 | 0.658 | 0.621 |
| 0 | 0 | 0 | 0 | 0 | 1 | 8 | 0 | 0.604 | 0.109 |
| 1 | 0 | 1 | 0 | 0 | 1 | 6 | 0 | 0.543 | 0.450 |
| 0 | 0 | 0 | 0 | 0 | 0 | 2 | 0 | 0.509 | 0.070 |
| 0 | 0 | 1 | 0 | 1 | 1 | 2 | 0 | 0.454 | 0.252 |
| 1 | 1 | 1 | 1 | 1 | 1 | 4 | 0 | 0.245 | 0.000 |
| 1 | 1 | 1 | 1 | 1 | 0 | 4 | 0 | 0.217 | 0.000 |
| 0 | 0 | 1 | 0 | 1 | 0 | 3 | 0 | 0.190 | 0.000 |
| 1 | 1 | 0 | 1 | 0 | 0 | 2 | 0 | 0.127 | 0.000 |
| 0 | 1 | 0 | 1 | 0 | 0 | 4 | 0 | 0.033 | 0.000 |

1. Weight outcomes

Table 3. Truth table for the main configuration – outcome set positively

| **Goal-setting** | **Education** | **Counseling** | **Social** | **Self-monitoring** | **Follow-up duration** | **Observations** | **Outcome** | **Raw consist.** | **PRI consist.** |
| --- | --- | --- | --- | --- | --- | --- | --- | --- | --- |
| 1 | 1 | 1 | 0 | 1 | 0 | 5 | 1 | 1.000 | 1.000 |
| 1 | 0 | 1 | 0 | 1 | 1 | 2 | 1 | 0.952 | 0.878 |
| 0 | 1 | 1 | 1 | 1 | 1 | 3 | 1 | 0.819 | 0.609 |
| 1 | 0 | 0 | 0 | 0 | 1 | 5 | 0 | 0.448 | 0.058 |
| 1 | 1 | 0 | 0 | 0 | 0 | 4 | 0 | 0.366 | 0.011 |
| 0 | 1 | 1 | 0 | 0 | 1 | 3 | 0 | 0.013 | 0.000 |
| 0 | 0 | 0 | 0 | 0 | 1 | 5 | 0 | 0.000 | 0.000 |

Table 4. Truth table for the configuration presented in the result section– outcome set positively

| **Goal-setting** | **Messaging** | **Counseling** | **Theory** | **Condition** | **Follow-up duration** | **Observations** | **Outcome** | **Raw consist.** | **PRI consist.** |
| --- | --- | --- | --- | --- | --- | --- | --- | --- | --- |
| 1 | 1 | 1 | 0 | 1 | 0 | 2 | 1 | 0.950 | 0.935 |
| 1 | 0 | 1 | 1 | 1 | 0 | 4 | 1 | 0.828 | 0.788 |
| 1 | 1 | 0 | 0 | 0 | 1 | 5 | 0 | 0.448 | 0.058 |
| 0 | 0 | 1 | 0 | 1 | 1 | 6 | 0 | 0.417 | 0.194 |
| 1 | 1 | 0 | 1 | 1 | 0 | 4 | 0 | 0.366 | 0.011 |
| 0 | 0 | 0 | 0 | 0 | 1 | 6 | 0 | 0.000 | 0.000 |
