# Supplementary material for: Fitbit-Based Interventions for Healthy Lifestyle Outcomes: Systematic Review and Meta-Analysis
Source: J Med Internet Res. 2020 Oct 12;22(10):e23954. doi: 10.2196/23954 (PMC7589007; doi:10.2196/23954)
Supplement: Multimedia Appendix 13 [file jmir_v22i10e23954_app13.docx]

1. PA outcomes

Figure 1. Configuration chart for the main configuration – outcome set positively


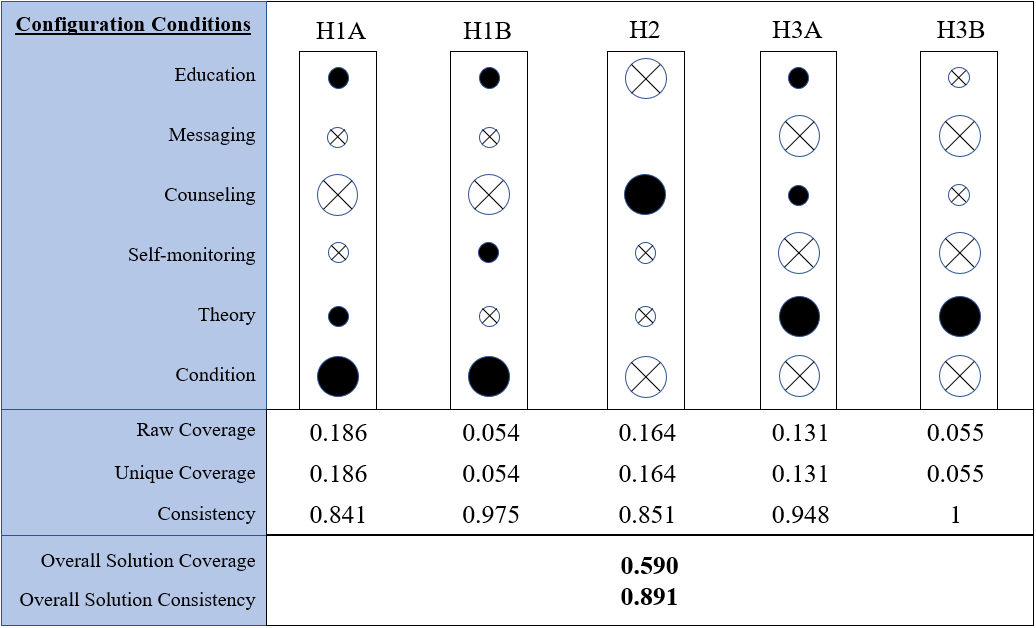


H1A: Theory-based interventions that included education, but without messaging, counseling and self-monitoring lead to better outcomes for individuals with pre-existing condition.

H1B: Non-theory-based interventions that included education and self-monitoring, but without messaging and counseling lead to better outcomes for individuals with pre-existing condition.

H2: Non-theory-based interventions that included counseling, but without education and self-monitoring lead to better outcomes for individuals with no pre-existing condition.

H3A: Theory-based interventions that included education and counseling, but without messaging and self-monitoring lead to better outcomes for individuals with no pre-existing condition.

H3B: Theory-based interventions that did not include education, messaging, counseling and self-monitoring lead to better outcomes for individuals with no pre-existing condition.

Figure 2. Configuration chart for the configuration presented in the result section– outcome set positively


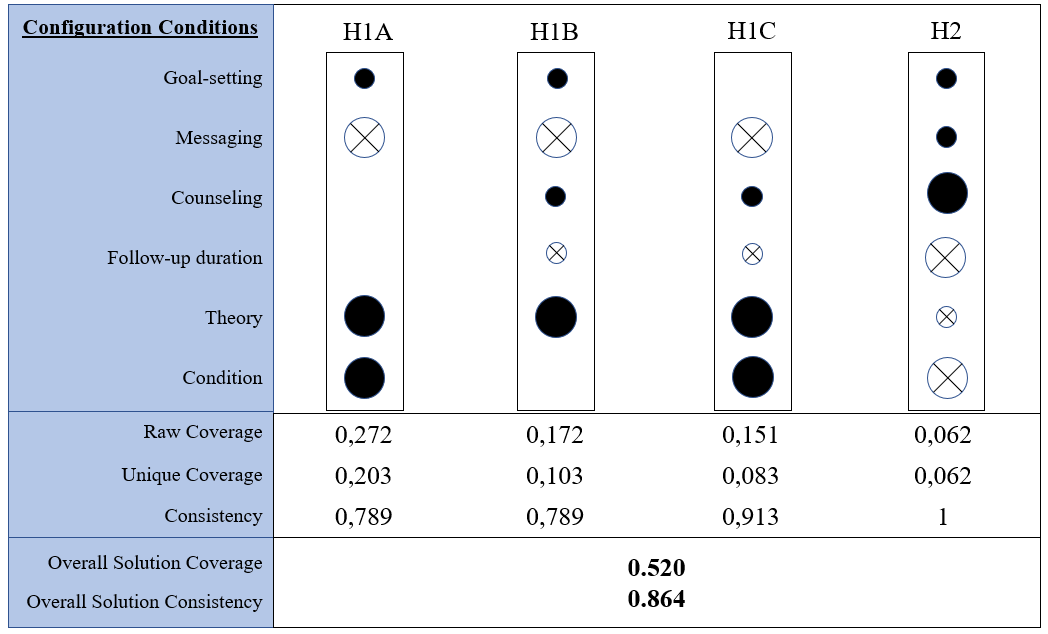


H1A: Theory-based interventions that included goal setting, but without messaging lead to better outcomes for individuals with pre-existing condition.

H1B: Theory-based interventions that included goal setting and counseling, but without messaging lead to better outcomes in short-term studies.

H1C: Theory-based interventions that included counseling, but without messaging lead to better outcomes for individuals with pre-existing condition in short-term studies.

H2: Non-theory-based interventions that included goal setting, messaging and counseling lead to better outcomes for individuals with no pre-existing condition in short-term studies.

1. Weight outcomes

Figure 3. Configuration chart for the main configuration – outcome set positively


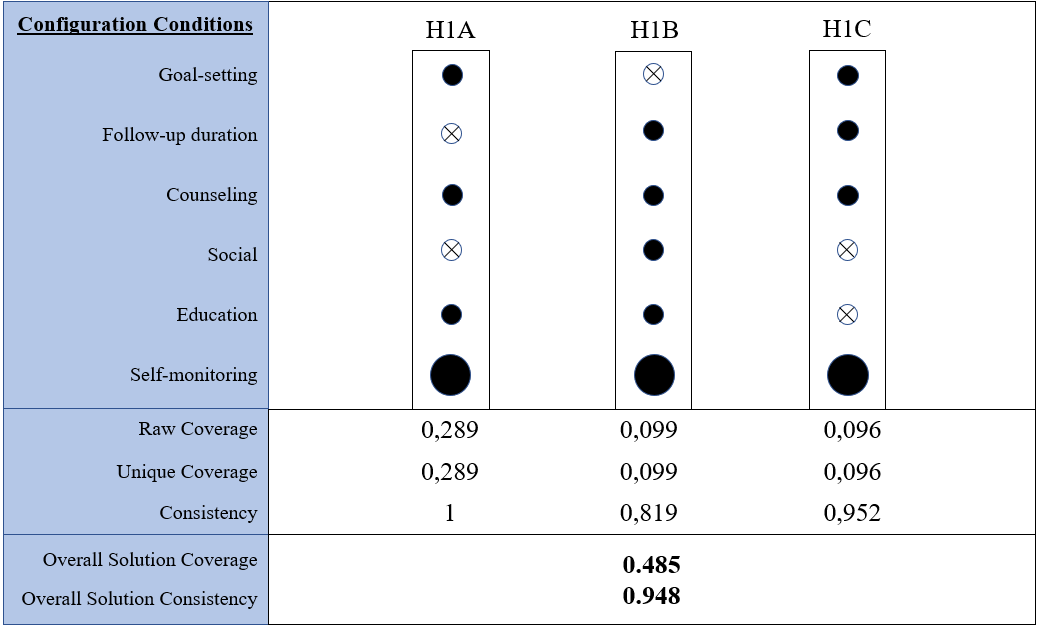


H1A: Interventions that included goal setting, counseling, education and self-monitoring, but without social lead to better outcomes in short-term studies.

H1B: Interventions that included counseling, social, education and self-monitoring, but without goal setting lead to better outcomes in long-term studies.

H1C: Interventions that included goal setting, counseling and self-monitoring, but without social and education lead to better outcomes in long-term studies.

Figure 4. Configuration chart for the configuration presented in the result section– outcome set positively


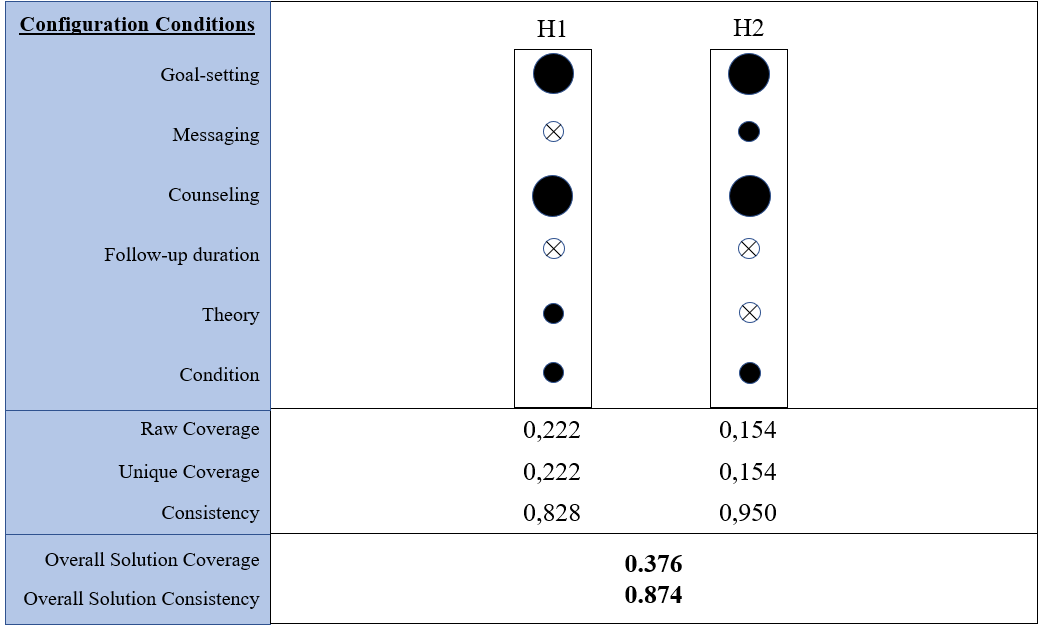


H1: Theory-based interventions that included goal setting and counseling, but without messaging lead to better outcomes for individuals with pre-existing condition in short-term studies.

H2: Non-theory-based interventions that included goal setting, messaging and counseling lead to better outcomes for individuals with pre-existing condition in short-term studies.
