# Supplementary material for: Fitbit-Based Interventions for Healthy Lifestyle Outcomes: Systematic Review and Meta-Analysis
Source: J Med Internet Res. 2020 Oct 12;22(10):e23954. doi: 10.2196/23954 (PMC7589007; doi:10.2196/23954)
Supplement: Multimedia Appendix 14 [file jmir_v22i10e23954_app14.docx]

| **Outcome** | **Goal-setting** | **Messaging** | **Education** | **Counseling** | **Social** | **Self-monitoring** | **Theory** | **Condition** | **Follow-up duration** | **Healthy lifestyle improvement** |
| --- | --- | --- | --- | --- | --- | --- | --- | --- | --- | --- |
| MVPA | 1 | 1 | 1 | 1 | 0 | 0 | 1 | 1 | 0.5001 | 0.00 |
| Weight | 0 | 0 | 0 | 0 | 0 | 0 | 0 | 0 | 0.83 | 0.00 |
| Weight | 0 | 0 | 0 | 0 | 0 | 0 | 0 | 0 | 0.83 | 0.00 |
| Weight | 0 | 0 | 0 | 0 | 0 | 0 | 0 | 0 | 0.83 | 0.00 |
| Weight | 0 | 0 | 0 | 0 | 0 | 0 | 0 | 0 | 0.83 | 0.00 |
| Weight | 0 | 0 | 0 | 0 | 0 | 0 | 0 | 0 | 0.83 | 0.00 |
| Weight | 1 | 1 | 0 | 0 | 0 | 0 | 0 | 0 | 0.83 | 0.00 |
| MVPA | 0 | 0 | 0 | 0 | 0 | 1 | 1 | 0 | 0.02 | 0.00 |
| Weight | 0 | 0 | 1 | 0 | 0 | 0 | 0 | 0 | 0.5001 | 0.00 |
| Sedentary behavior | 0 | 0 | 1 | 0 | 0 | 0 | 0 | 0 | 0.5001 | 0.00 |
| Weight | 1 | 1 | 0 | 1 | 0 | 0 | 0 | 0 | 0.25 | 0.00 |
| Steps | 1 | 0 | 0 | 1 | 0 | 0 | 0 | 1 | 0.08 | 0.00 |
| Weight | 1 | 1 | 1 | 0 | 0 | 0 | 1 | 1 | 0.27 | 0.00 |
| Sedentary behavior | 0 | 0 | 1 | 1 | 0 | 0 | 0 | 1 | 0.08 | 0.00 |
| MVPA | 0 | 0 | 1 | 1 | 0 | 0 | 0 | 1 | 0.08 | 0.00 |
| MVPA | 1 | 1 | 0 | 0 | 1 | 0 | 1 | 0 | 0.21 | 0.00 |
| Steps | 1 | 1 | 0 | 1 | 0 | 0 | 1 | 1 | 0.44 | 0.00 |
| MVPA | 1 | 1 | 0 | 1 | 0 | 0 | 1 | 1 | 0.44 | 0.00 |
| MVPA | 1 | 1 | 0 | 1 | 0 | 0 | 1 | 1 | 0.44 | 0.00 |
| Weight | 0 | 0 | 1 | 1 | 0 | 0 | 0 | 1 | 0.5001 | 0.00 |
| Weight | 0 | 0 | 1 | 1 | 0 | 0 | 0 | 1 | 0.5001 | 0.00 |
| Steps | 0 | 1 | 1 | 0 | 0 | 0 | 1 | 0 | 0.25 | 0.00 |
| MVPA | 0 | 1 | 1 | 0 | 0 | 0 | 1 | 0 | 0.25 | 0.00 |
| MVPA | 0 | 1 | 1 | 0 | 0 | 0 | 1 | 0 | 0.25 | 0.00 |
| Weight | 0 | 0 | 1 | 1 | 0 | 0 | 0 | 1 | 0.5001 | 0.02 |
| Sedentary behavior | 0 | 0 | 0 | 1 | 0 | 0 | 0 | 1 | 0.75 | 0.05 |
| Weight | 1 | 1 | 1 | 0 | 0 | 0 | 1 | 1 | 0.27 | 0.07 |
| MVPA | 1 | 1 | 1 | 1 | 0 | 0 | 1 | 1 | 0.5001 | 0.09 |
| Steps | 1 | 0 | 1 | 1 | 0 | 0 | 0 | 0 | 1.00 | 0.09 |
| MVPA | 0 | 1 | 1 | 0 | 0 | 0 | 1 | 0 | 0.25 | 0.10 |
| MVPA | 0 | 0 | 1 | 0 | 0 | 0 | 0 | 0 | 0.5001 | 0.15 |
| Sedentary behavior | 0 | 0 | 1 | 0 | 0 | 0 | 0 | 0 | 0.5001 | 0.17 |
| Weight | 1 | 0 | 1 | 1 | 0 | 0 | 1 | 1 | 0.25 | 0.18 |
| Sedentary behavior | 1 | 1 | 0 | 0 | 1 | 0 | 1 | 0 | 0.21 | 0.20 |
| Steps | 0 | 0 | 1 | 0 | 0 | 0 | 0 | 0 | 0.13 | 0.20 |
| MVPA | 1 | 0 | 0 | 1 | 0 | 0 | 0 | 0 | 0.5001 | 0.21 |
| Weight | 0 | 0 | 1 | 1 | 1 | 1 | 0 | 1 | 0.5001 | 0.23 |
| MVPA | 1 | 1 | 0 | 1 | 0 | 0 | 1 | 1 | 0.440 | 0.23 |
| Sedentary behavior | 0 | 0 | 1 | 0 | 0 | 0 | 0 | 0 | 0.5001 | 0.24 |
| Steps | 1 | 1 | 1 | 1 | 0 | 0 | 1 | 1 | 0.5001 | 0.25 |
| Sedentary behavior | 1 | 0 | 1 | 0 | 1 | 0 | 1 | 1 | 0.5001 | 0.29 |
| MVPA | 0 | 0 | 1 | 1 | 0 | 0 | 0 | 1 | 0.08 | 0.32 |
| Weight | 1 | 1 | 0 | 0 | 0 | 0 | 0 | 0 | 0.83 | 0.33 |
| MVPA | 1 | 1 | 1 | 1 | 0 | 0 | 1 | 1 | 0.5001 | 0.35 |
| MVPA | 0 | 0 | 1 | 0 | 0 | 0 | 0 | 0 | 0.5001 | 0.36 |
| Steps | 0 | 0 | 1 | 0 | 0 | 0 | 0 | 0 | 0.5001 | 0.36 |
| Steps | 1 | 0 | 1 | 1 | 0 | 0 | 0 | 0 | 1.00 | 0.37 |
| Weight | 1 | 1 | 0 | 1 | 0 | 0 | 0 | 1 | 0.5001 | 0.40 |
| Steps | 0 | 0 | 0 | 0 | 0 | 0 | 0 | 0 | 0.21 | 0.40 |
| Sedentary behavior | 1 | 0 | 1 | 0 | 1 | 0 | 1 | 1 | 0.5001 | 0.44 |
| Weight | 1 | 1 | 0 | 0 | 0 | 0 | 0 | 0 | 0.83 | 0.46 |
| Weight | 1 | 1 | 1 | 0 | 0 | 0 | 1 | 1 | 0.27 | 0.49 |
| Weight | 1 | 1 | 1 | 0 | 0 | 0 | 1 | 1 | 0.27 | 0.51 |
| Weight | 1 | 1 | 0 | 0 | 0 | 0 | 0 | 0 | 0.83 | 0.52 |
| Steps | 1 | 1 | 0 | 1 | 0 | 0 | 0 | 1 | 0.5001 | 0.55 |
| Weight | 1 | 1 | 0 | 0 | 0 | 0 | 0 | 0 | 0.83 | 0.55 |
| Weight | 1 | 0 | 0 | 1 | 0 | 1 | 1 | 1 | 0.5001 | 0.55 |
| MVPA | 0 | 0 | 1 | 0 | 0 | 0 | 0 | 0 | 0.5001 | 0.57 |
| MVPA | 1 | 0 | 0 | 1 | 0 | 1 | 1 | 1 | 1.00 | 0.59 |
| Sedentary behavior | 1 | 0 | 1 | 1 | 0 | 0 | 1 | 1 | 0.25 | 0.61 |
| MVPA | 0 | 0 | 0 | 0 | 0 | 1 | 1 | 0 | 0.02 | 0.62 |
| MVPA | 0 | 0 | 1 | 0 | 0 | 0 | 0 | 0 | 0.5001 | 0.64 |
| MVPA | 1 | 0 | 0 | 1 | 0 | 1 | 1 | 1 | 0.5001 | 0.64 |
| MVPA | 0 | 0 | 0 | 1 | 0 | 0 | 0 | 1 | 0.75 | 0.66 |
| MVPA | 1 | 0 | 1 | 1 | 0 | 1 | 1 | 1 | 0.31 | 0.67 |
| Weight | 1 | 0 | 0 | 1 | 0 | 1 | 0 | 1 | 0.75 | 0.69 |
| Weight | 0 | 0 | 1 | 1 | 1 | 1 | 0 | 1 | 0.5001 | 0.70 |
| Sedentary behavior | 0 | 0 | 1 | 1 | 0 | 0 | 1 | 1 | 0.17 | 0.70 |
| MVPA | 1 | 0 | 1 | 1 | 0 | 0 | 1 | 1 | 0.25 | 0.71 |
| Weight | 0 | 0 | 1 | 1 | 1 | 1 | 0 | 1 | 0.5001 | 0.72 |
| Steps | 0 | 0 | 1 | 1 | 0 | 0 | 1 | 1 | 0.17 | 0.72 |
| MVPA | 1 | 0 | 1 | 1 | 1 | 0 | 1 | 0 | 0.25 | 0.74 |
| Steps | 1 | 1 | 0 | 1 | 0 | 0 | 0 | 0 | 0.25 | 0.75 |
| MVPA | 0 | 0 | 1 | 1 | 0 | 0 | 1 | 1 | 0.17 | 0.75 |
| Weight | 1 | 1 | 1 | 1 | 0 | 1 | 0 | 1 | 0.25 | 0.76 |
| Steps | 1 | 0 | 0 | 0 | 1 | 0 | 0 | 0 | 0.25 | 0.79 |
| MVPA | 1 | 1 | 1 | 0 | 0 | 0 | 1 | 1 | 0.27 | 0.82 |
| MVPA | 0 | 0 | 1 | 1 | 0 | 0 | 1 | 1 | 0.17 | 0.90 |
| Weight | 1 | 1 | 1 | 1 | 0 | 1 | 0 | 1 | 0.25 | 0.94 |
| Steps | 0 | 0 | 1 | 0 | 0 | 1 | 0 | 1 | 0.44 | 0.95 |
| MVPA | 1 | 0 | 1 | 0 | 1 | 0 | 1 | 1 | 0.5001 | 1.00 |
| Steps | 1 | 0 | 1 | 0 | 1 | 0 | 1 | 1 | 0.5001 | 1.00 |
| Weight | 1 | 0 | 1 | 0 | 1 | 0 | 1 | 1 | 0.5001 | 1.00 |
| Steps | 1 | 0 | 1 | 0 | 1 | 0 | 1 | 1 | 0.25 | 1.00 |
| MVPA | 1 | 0 | 1 | 0 | 1 | 0 | 1 | 1 | 0.25 | 1.00 |
| Sedentary behavior | 1 | 0 | 1 | 0 | 1 | 0 | 1 | 1 | 0.25 | 1.00 |
| Weight | 1 | 0 | 1 | 1 | 0 | 1 | 1 | 1 | 0.31 | 1.00 |
| Weight | 1 | 0 | 1 | 1 | 0 | 1 | 1 | 1 | 0.31 | 1.00 |
| Weight | 1 | 0 | 1 | 1 | 0 | 1 | 1 | 1 | 0.31 | 1.00 |
| MVPA | 1 | 0 | 1 | 1 | 1 | 0 | 1 | 0 | 0.25 | 1.00 |
| MVPA | 1 | 0 | 1 | 1 | 1 | 0 | 1 | 0 | 0.25 | 1.00 |
| MVPA | 1 | 0 | 1 | 1 | 1 | 0 | 1 | 0 | 0.25 | 1.00 |
| Steps | 1 | 0 | 1 | 1 | 1 | 0 | 1 | 0 | 0.25 | 1.00 |
| MVPA | 1 | 0 | 0 | 1 | 0 | 0 | 0 | 0 | 0.5001 | 1.00 |
| Steps | 1 | 0 | 0 | 1 | 0 | 0 | 0 | 0 | 0.5001 | 1.00 |
| Steps | 1 | 0 | 0 | 1 | 0 | 0 | 0 | 0 | 0.5001 | 1.00 |
| MVPA | 1 | 1 | 0 | 1 | 0 | 0 | 0 | 0 | 0.25 | 1.00 |
| MVPA | 0 | 0 | 0 | 0 | 0 | 0 | 1 | 0 | 0.02 | 1.00 |
| MVPA | 0 | 0 | 0 | 0 | 0 | 0 | 1 | 0 | 0.02 | 1.00 |
| MVPA | 1 | 1 | 0 | 1 | 0 | 0 | 0 | 0 | 0.25 | 1.00 |
| Steps | 1 | 0 | 1 | 0 | 0 | 1 | 0 | 1 | 0.44 | 1.00 |
| Steps | 1 | 0 | 1 | 0 | 0 | 0 | 1 | 1 | 0.31 | 1.00 |
